# DNA-encoded chemistry technology yields expedient access to SARS-CoV-2 M^pro^ inhibitors

**DOI:** 10.1073/pnas.2111172118

**Published:** 2021-08-23

**Authors:** Srinivas Chamakuri, Shuo Lu, Melek Nihan Ucisik, Kurt M. Bohren, Ying-Chu Chen, Huang-Chi Du, John C. Faver, Ravikumar Jimmidi, Feng Li, Jian-Yuan Li, Pranavanand Nyshadham, Stephen S. Palmer, Jeroen Pollet, Xuan Qin, Shannon E. Ronca, Banumathi Sankaran, Kiran L. Sharma, Zhi Tan, Leroy Versteeg, Zhifeng Yu, Martin M. Matzuk, Timothy Palzkill, Damian W. Young

**Affiliations:** ^a^Center for Drug Discovery, Department of Pathology & Immunology, Baylor College of Medicine, Houston, TX 77030;; ^b^Department of Pharmacology and Chemical Biology, Baylor College of Medicine, Houston, TX 77030;; ^c^Department of Pediatrics, National School of Tropical Medicine, Baylor College of Medicine, Houston, TX 77030;; ^d^Center for Vaccine Development, Texas Children's Hospital, Houston, TX 77030;; ^e^Department of Molecular Biophysics and Integrated Bioimaging, Berkeley Center for Structural Biology, Lawrence Berkeley National Laboratory, Berkeley, CA 94720;; ^f^Verna and Marrs McLean Department of Biochemistry and Molecular Biology, Baylor College of Medicine, Houston, TX 77030

**Keywords:** antiviral, drug discovery, covalent inhibitors

## Abstract

Severe acute respiratory syndrome coronavirus 2 (SARS-CoV-2) has killed more than 4 million humans globally, but there is no bona fide Food and Drug Administration–approved drug-like molecule to impede the COVID-19 pandemic. The sluggish pace of traditional therapeutic discovery is poorly suited to producing targeted treatments against rapidly evolving viruses. Here, we used an affinity-based screen of 4 billion DNA-encoded molecules en masse to identify a potent class of virus-specific inhibitors of the SARS-CoV-2 main protease (M^pro^) without extensive and time-consuming medicinal chemistry. CDD-1714, the initial three-building-block screening hit (molecular weight [MW] = 542.5 g/mol), was a potent inhibitor (inhibition constant [*K*_i_] = 20 nM). CDD-1713, a smaller two-building-block analog (MW = 353.3 g/mol) of CDD-1714, is a reversible covalent inhibitor of M^pro^ (*K*_i_ = 45 nM) that binds in the protease pocket, has specificity over human proteases, and shows in vitro efficacy in a SARS-CoV-2 infectivity model. Subsequently, key regions of CDD-1713 that were necessary for inhibitory activity were identified and a potent (*K*_i_ = 37 nM), smaller (MW = 323.4 g/mol), and metabolically more stable analog (CDD-1976) was generated. Thus, screening of DNA-encoded chemical libraries can accelerate the discovery of efficacious drug-like inhibitors of emerging viral disease targets.

## Significance

SARS-CoV-2 has had a crippling impact on human life globally. Vaccine development has been used as a first-line strategy for COVID-19 prevention and mitigation; however, small-molecule drugs are still vitally needed to extend treatment options. Traditional screening methods for identifying biologically active small molecules are sluggish and often sample an insufficient number of compounds to identify suitable hits. Here, we applied a screening method known as DNA-encoded chemistry technology (DEC-Tec) to screen billions of compounds against a critical viral protein, M^pro^. In rapid fashion, we identified the compound CDD-1713 as a potent and selective M^pro^ inhibitor. This study illuminates DEC-Tec as a highly expeditious strategy toward generating small molecules against critical targets of infectious agents.

On March 11, 2020, the World Health Organization recognized the COVID-19 outbreak caused by severe acute respiratory syndrome coronavirus 2 (SARS-CoV-2) as a pandemic. Although vaccines are being approved for emergency use to fight COVID-19 (https://www.cdc.gov/vaccines/covid-19/index.html), there is an urgent need for effective new drug treatments to reduce the morbidity and mortality for patients who have already contracted COVID-19, not yet received the vaccine, are infected with SARS-CoV-2 variants that are more resistant to the vaccines, and for future coronavirus pandemics. The conventional process of drug discovery is based on automated high-throughput screening (HTS) or structure-based drug design. HTS requires a complex infrastructure, the development a miniaturized assay tailored to the individual target, and generally the need for extensive medicinal chemistry to optimize modestly potent hits that arise from the screen. The development of such screening methods is therefore sluggish and not well-suited to meet the present public health demands imposed by the COVID-19 pandemic. As an HTS alternative, structure-based approaches are advantageous when available since the rational synthesis of analogs can accelerate the generation of potent compounds. Prior to our work, other investigations have relied on structure-based methods and computational approaches to optimize moderately potent M^pro^ inhibitors (1, 2). However, the need for a high-resolution X-ray structure to initiate a drug discovery campaign can be extremely rate-limiting, if not altogether prohibitive. Finally, drug repurposing is often utilized as an efficient discovery strategy since it can bypass issues related to safety; however, the application of a compound to a target for which it was not optimized may never achieve maximal therapeutic benefit. A screening process which circumvents these challenges would significantly accelerate the pace for identification of clinical candidate compounds. DNA-encoded chemistry technology (DEC-Tec) is an increasingly attractive strategy to explore chemical space to identify small molecules and high-affinity binders for a multitude of protein targets (3–7). DEC-Tec involves the creation of libraries of drug-like molecules covalently attached to a unique DNA barcode that enables identification of binders for a target in a pool of millions to billions of compounds. DEC-Tec addresses these pitfalls related to HTS by screening of billions of DNA-tagged small molecules as a single mixture using an affinity selection assay (4, 8–13). This expanded number of drug-like small molecules allows for the identification of high-affinity ligands using a technologically practical format. For example, the modest protein requirement and low price of DNA sequencing both contribute to the inexpensive cost of screening small-molecule DNA-encoded chemical libraries. Additionally, the various steps involved in the selection process, which include the affinity selection to the protein, DNA sample preparation, sequencing, and data analysis, can each be performed in several days, providing for a highly expeditious screening process. Finally, the large number of compounds employed in DEC-Tec is leveraged to provide binders in the absence of preexisting structural information related to the target or its ligand-binding preferences. We believe that these features of DEC-Tec provided clear advantages for discovering novel small-molecule agents against SARS-CoV-2 targets.

With our collection of over 55 DEC-Tec libraries (4 billion unique molecules) including protease-biased libraries (6) and various libraries utilizing DNA-compatible reactions developed in-house (14–22), the Center for Drug Discovery at Baylor College of Medicine has the operational capacity to support emergent action at the onset of pandemics. The genome of SARS-CoV-2 comprises six major open reading frames including two polyproteins that undergo extensive proteolytic processing to create functional proteins that perform tasks essential for viral propagation (23). This processing is largely achieved by SARS-CoV-2 main protease (M^pro^ or 3CL^pro^) (24), a cysteine protease enzyme indispensable for the virus lifecycle and a key therapeutic target. Our hypothesis is that SARS-CoV-2 M^pro^ screening of billions of DNA-encoded molecules, generation of small-molecule-M^pro^ cocrystals, and minimal medicinal chemistry follow-up would generate drug-like inhibitors of M^pro^ for emergent use in patients infected with SARS-CoV-2, related variants, and related coronaviruses.

To screen our DEC-Tec collection, we first constructed the pSUMO-SARS-CoV-2-M^pro^ plasmid carrying the SUMO-M^pro^-His6 tag (*SI Appendix*, Fig. S1). The M^pro^ open reading frame sequence was flanked on the N terminus by its endogenous cleavage site (SAVLQ↓SGFRK) and on its C terminus by a PreScission cleavage site (SGVTFQ↓GP). The SUMO-M^pro^-His6 recombinant fusion protein was expressed from *Escherichia coli* BL21(DE3), and the M^pro^ enzyme with its authentic N terminus and the His6 tag was purified as described in *Methods* and *SI Appendix*, Fig. S2. Because our nickel magnetic screen capture test requires a His tag, the M^pro^-His6 protein was used for DEC-Tec library screening. For crystallography and enzymatic assays, the M^pro^-His6 protein was treated with PreScission enzyme to remove the C-terminal 6-His tag, and the M^pro^ was further purified by gel filtration chromatography. Using a fluorescent peptide, Dabcyl-KTSAVLQSGFRKM-E(Edans)-NH_2_, as a reporter substrate, we first confirmed that the purified recombinant M^pro^-His6 and the M^pro^ (“native”) lacking the His6 tag were very active and demonstrated nearly identical bioactivity in vitro.

**Fig. 1.**
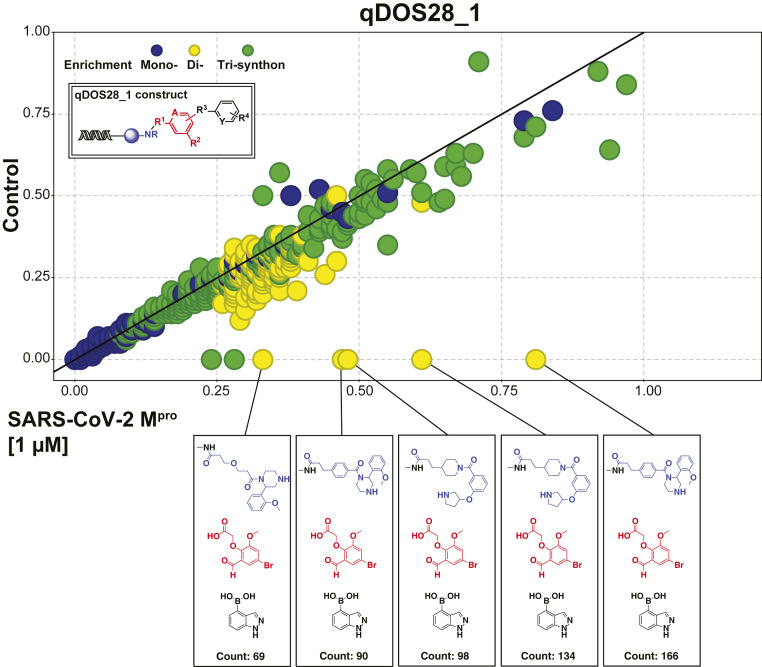
Enrichment profile of qDOS28_1 against SARS-CoV-2 M^pro^ at 1 μM. The selection data have shown the enrichment of the same BB2 (in red) and BB3 (in black) with various BB1 (in blue), where the BB1 features di-substituted amine. The methyl amide (in black) represents the DNA attachment point. The number of counts in the box represents the number of observed library members.

Forty unique DNA-encoded chemical libraries (DECLs) cumulatively containing 3.987 billion drug-like compounds were pooled together for the screen of M^pro^-His6 binding compounds. Each library was prequantitated by qPCR, and library pooling was conducted to have 1 million copies of each compound present in the pool. Our selection for M^pro^-His6 binders comprised a three-round affinity selection with an M^pro^-His6 protein concentration of 1 µM. An independent affinity selection was performed in parallel without protein to serve as a no-target control to identify any nonprotein specific enrichment. Our Illumina next-generation sequencing identified a chemical series consistently enriched with excellent structure–enrichment relationships (SER) from qDOS28_1, one of our DEC-Tec libraries (Fig. 1 and *SI Appendix*, Scheme S1). The process of modern HTS screening has the capability to interrogate the activity of ∼100,000 small molecules per day in a specialized well-based assay (25, 26). Here, DEC-Tec was used to evaluate 4 billion small molecules as a single mixture in 1 d against M^pro^. Thus DEC-Tec, through its breakneck rate of screening, offers a tremendous advantage to infectious disease targets of pathogens that pose both present and imminent public health threats.

**Fig. 2.**
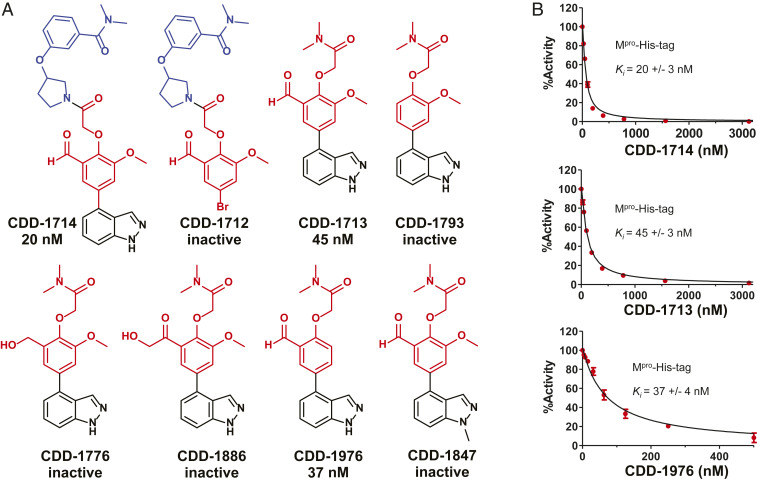
SARS-CoV-2 M^pro^ hits, analogs and examples of enzymatic inhibitory activity of these key molecules. (*A*) Small molecules synthesized off DNA are shown. Numbers indicate *K*_i_ values determined as described in *Methods*. Inactive = compounds that inhibited M^pro^ activity by less than 90% with 25 µM compound were considered inactive. (*B*) Inhibition *K*_i_ value determination against M^pro^-His6. Concentration-dependent inhibition curve of CDD-1714, CDD-1713, and CDD-1976.

The DNA sequences resulting from the M^pro^ selection were analyzed (27) to determine structural features that were enriched to prioritize compounds to be synthesized without the DNA barcode (off DNA). Compounds within DECLs are generally constructed from the union of three building blocks (trisynthons). Compounds enriched from the selection can be analyzed for those sharing one or two building blocks (monosynthons vs. disynthons) in common, leading to the identification of critical structure–enrichment relationships. The consideration of these SER leads to potent compounds in an efficient manner, supplanting the need for many rounds of laborious medicinal chemistry. Based on the M^pro^ selection analysis, the trisynthon CDD-1714 (“hit” molecule) and its smaller disynthons CDD-1712 and CDD-1713 (Fig. 2) were synthesized off DNA in two to four steps from commercially available materials (*SI Appendix*). To characterize the potency of selected compounds toward M^pro^, we utilized the above described fluorescent peptide reporter assay. For initial compound screening, 25 µM of compound was incubated with M^pro^ and only compounds which inhibited M^pro^ proteolytic activity >90% were considered as candidates. The inhibition constant (*K*_i_) values of these compounds were determined with concentrations ranging from 4 nM to 4,000 nM (*SI Appendix*, Fig. S3 and Table S1). Since M^pro^-His6 was used in library screening, we performed parallel enzyme inhibition assays using either M^pro^-His6 or M^pro^ to evaluate the potency of compounds toward M^pro^ proteolytic activity in the presence and absence of His tag. Using this protease inhibition assay, we found that CDD-1713 and CDD-1714 inhibited M^pro^ with *K*_i_ values of 45 nM and 20 nM, respectively (Fig. 2 and *SI Appendix*, Fig. S3). These results indicated that building block 1 (BB1), closest to the DNA attachment site, was less critical for binding and inhibition; CDD-1713 was chosen for optimization efforts based on its low molecular weight (353.3 g/mol) and good cLogP (2.01). CDD-1713 contains a reactive aldehyde functional group capable of forging covalent bonds with proteins, and thus we initially turned our attention toward examining the importance of the aldehyde moiety. Deleting the aldehyde (CDD-1793) or replacing with hydroxymethyl (CDD-1776) completely abolished the activity, while replacing the aldehyde with hydroxymethyl ketone (CDD-1886) drastically decreased M^pro^ inhibition by greater than 100-fold (Fig. 2). We therefore concluded that the aldehyde was required for activity and next proceeded to further probe its electrophilic nature. Generation of the more electron-deficient des-methoxy analog (CDD-1976) showed better inhibition compared to CDD-1713. Aldehydes have the propensity to react nonselectively; however, CDD-1712 did not show any activity, and additionally we synthesized CDD-1847 which incorporates an *N*-methylindazole and found that it completely lost activity. Given this remote substitution to the aldehyde, it is likely that the M^pro^ inhibitory activity of CDD-1713 and related analogs results from a specific binding interaction with M^pro^. While aldehydes may pose a liability in drugs, compounds that contain aldehydes have been evaluated in humans, demonstrating good selectivity and other drug-like properties (28–33). To confirm that the potent inhibitors bound tightly to M^pro^, we performed a protein thermal shift stability assay (thermofluor assay). CDD-1713, CDD-1714, and CDD-1976 cause a concentration-dependent stabilization of M^pro^, with CDD-1976 showing the most pronounced temperature shift at all three protein concentrations (*SI Appendix*, Fig. S4). Taken altogether, the DEC-Tec process involving affinity selection of 4 billion DNA-encoded compounds against M^pro^, analysis of SER, and synthesis of the exemplary compounds (including with different electrophiles) off DNA (*SI Appendix*, Fig. S5 and Table S1) yielded potent and selective inhibitors of M^pro^. We note that CDD-1713, which was inferred directly from the selection, was synthesized and validated in rapid fashion (less than 10 wk from start to finish; *SI Appendix*, Table S2), highlighting DEC-Tec’s ability to produce potent compounds without extensive synthetic optimization.

Using the M^pro^ sequence, BLAST search analysis of the reference proteins encoded by the human genome shows no significant similarity. To confirm that our M^pro^ inhibitors do not show any potential off-target inhibition of major proteases in humans, we tested their effects on four important proteases representing four classes of human protease enzymes. As might be expected from our BLAST search, none of our inhibitors block the enzymatic activity of cathepsin B (a cysteine protease like M^pro^), thrombin (a serine protease), renin (an aspartic protease), or matrix metallopeptidase-1 (MMP-1; *SI Appendix*, Fig. S6). Thus, our potent molecules are anticipated to have specific effects on inhibition of viral protein processing in vivo without altering human cellular activity.

**Fig. 3.**
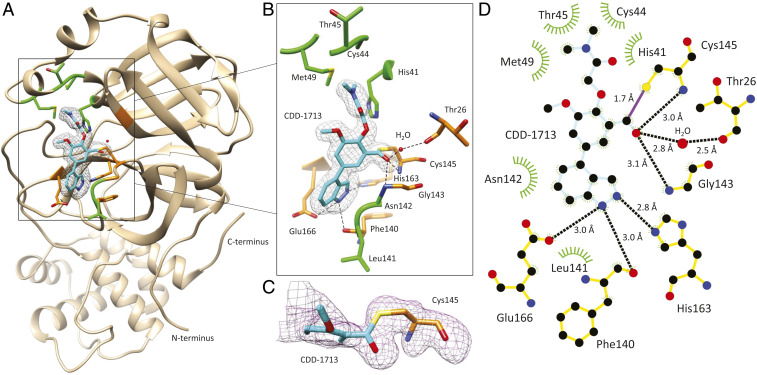
Crystal structure of M^pro^ in complex with CDD-1713. (*A*) Structure of M^pro^ (tan) with CDD-1713 (cyan). The F_o_ – F_c_ density map is shown for the inhibitor with contouring level at 3σ. The catalytic site is located within the square. (*B*) Magnified view of the catalytic center. Carbon atoms of the inhibitor are cyan, nitrogen atoms are blue, and oxygen atoms are red. The M^pro^ amino acid residues involved in CDD-1713 binding are shown as stick models and labeled. M^pro^ residues that form hydrogen bonds (dashed black lines) and van der Waals interactions with CDD-1713 are colored in orange and green, respectively. One water molecule involved in hydrogen bond was colored red. The site chain of Asn142 is not shown to avoid obstruction of the view of hydrogen bonds on the aldehyde group. (*C*) F_o_ − F_c_ omit maps showing electron density of the covalent bond formed between Cys145 and CDD-1713 contoured at 3s. Continuous electron density is present between C^13^ on the aldehyde group of CDD-1713 and thiol on the side chain of Cys145. (*D*) Two-dimensional diagram of the M^pro^ interaction with CDD-1713 generated by Ligplot^+^. Carbon atoms are shown in black, nitrogen atoms are blue, and oxygen atoms are red. Ligands are colored cyan and hydrogen bonds are represented as black dashed lines, hydrogen bond contacts are colored orange, and hydrophobic contacts are represented as green spoke arcs. Water molecule was presented as red sphere. The length of each hydrogen bond is labeled.

To understand the interactions of CDD-1713 with M^pro^ we determined the X-ray crystal structure of the enzyme in complex with the inhibitor. For this purpose, the purified enzyme was cocrystallized with CDD-1713. The structure was determined in space group C121 at 1.8-Å resolution with a single monomer in the asymmetric unit (Fig. 3 and *SI Appendix*, Table S3). The biological dimer is formed by the monomer and its symmetry-related monomer across the crystallographic two-fold axis, as seen previously in M^pro^ structures (1, 34).

Examination of the structure reveals CDD-1713 is positioned in the active site of M^pro^ with the electron density clearly showing a 1.7-Å covalent bond from the aldehyde of CDD-1713 to Sγ of the catalytic residue Cys145 (Fig. 3 *B* and *C*). The carbonyl oxygen of the aldehyde forms hydrogen bonds with the main chain nitrogens of Gly143 and Cys145 that form the oxyanion hole of the enzyme. Similar interactions have been reported for bicyclopropane-containing inhibitors of M^pro^ with an aldehyde warhead (1). The active site of M^pro^ contains four subsites (S1′, S1, S2, and S3) that accommodate the amino acids of the peptide substrate or peptidomimetic inhibitors (P1′, P1, P2, and P3) (35, 36). M^pro^ has a stringent requirement for a P1 glutamine occupying the S1 subsite (35). The indazole ring of CDD-1713 inserts into the S1 pocket (Fig. 3). The NH of the indazole group forms hydrogen bonds with the side-chain *O*ε of Glu166 and the main-chain *O* of Phe140, while the N of the indazole forms a hydrogen bond with the Nε2 of His163 (Fig. 3). In addition, the indazole group makes hydrophobic interactions with Phe140, Leu141, Asn142, and Glu166 (Fig. 3). The extensive interactions of the indazole with residues in the S1 pocket is of note in that the residues in the S1 pocket are largely conserved among coronavirus M^pro^ enzymes, suggesting CDD-1713 may exhibit broad M^pro^ specificity (37). The central phenyl ring of CDD-1713 makes hydrophobic interactions with Asn142 and positions the aldehyde group for interaction with Cys143. The *O*-alkyl chain on the central phenyl ring occupies a region between the S2 and S1′ subsites. The terminal methyl groups of the dimethylamide make hydrophobic interactions with His41, Cys44, Thr45, Ser46, and Met49 (Fig. 3*D*). Note that the dimethylamide is partially buried in the structure, suggesting the BB1 group in CDD-1714 and the DNA-attachment site may sterically clash with the Thr45-Met49 region. However, the rotatable bonds in the *O*-alkyl chain may allow positioning consistent with binding of the BB1 group and solvent exposure of the DNA-attachment point (Fig. 3*B*). Further, the methoxy group attached to the central phenyl ring is solvent-exposed and does not interact with M^pro^. The design of M^pro^ inhibitors has mostly centered on templates having multiple amide bonds to mimic the enzyme’s natural peptide substrates (1, 34, 38–41). CDD-1713 differs significantly from the amide-based inhibitors, allowing it to forge different interactions within the same M^pro^ active site (*SI Appendix*, Fig. S3*E*). Thus, the DEC-Tec approach supports the elucidation of novel pharmacophores to enzymes without specific design principles or prior knowledge of substrate preferences.

Using mouse and human liver microsomes, CDD-1713 was found to be metabolically labile in both mouse and human assays, while CDD-1976 is more stable in human liver microsomes, but not mouse (*SI Appendix*, Table S4). CDD-1713 and CDD-1976 displayed moderate cell permeability in an uptake assay of HepG2 cells (*SI Appendix*, Fig. S7*A*) and no obvious cytotoxicity in HepG2 cells was observed for both compounds at 100 μM (*SI Appendix*, Fig. S7*B*). Both CDD-1713 and CDD-1976 are relatively stable in human and mouse plasma; 80% of compounds remained in plasma after 2-h incubation (*SI Appendix*, Fig. S7*C*).

**Fig. 4.**
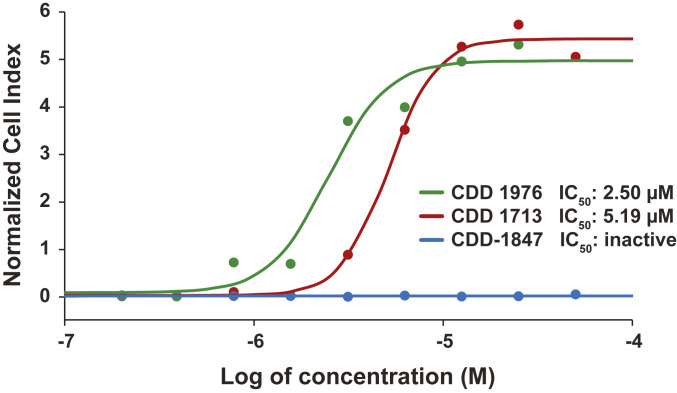
Normalized cell index plotted versus concentration (log) of M^pro^ drug compounds measured using the xCELLigence RTCA. Average data points from duplicate measurements. A sigmoidal dose–response curve was fitted to determine IC_50_ values for each M^pro^ drug compound (lines).

The viral inhibitory capacity of compounds CDD-1713 and CDD-1976 was demonstrated using a real-time cell analysis (RTCA) assay. To evaluate viral inhibition, different concentrations of the M^pro^ inhibitors were added to VERO E6 cells incubated with a live strain of the SARS-CoV-2 virus. Subsequently, the growth kinetics of the cells were followed over 75 h. In the positive control group, SARS-CoV-2 effectively caused cell death, which was observed as a decrease in the normalized cell index compared to the negative control group (no virus). CDD-1713 and CDD-1976 successfully reduced cell death in a dose-dependent manner, indicating that virus replication was stopped (Fig. 4). CDD-1976 was found to be most effective, with a calculated half-maximal inhibitory concentration (IC_50_) of 2.50 µM, followed by CDD-1713 with an IC_50_ of 5.19 µM. CDD-1847, a closely related analog that abolishes binding to M^pro^, was unable to prevent cell death by the SARS-CoV-2 virus.

In summary, using a DEC-Tec–based strategy we identified CDD-1713 and CDD-1976 as a class of potent and selective inhibitors of SARS-CoV-2 M^pro^ that block viral reproduction in a short span of time. X-ray crystallography was further deployed to elucidate the structural details of M^pro^ inhibition by CDD-1713, and this information should enable further development of drug-like M^pro^ inhibitors. These studies support DEC-Tec as an expedient and effective paradigm for generating therapeutics against critical targets within the SARS-CoV-2 genome.

## Methods

### Plasmid Construction.

The *E. coli* strain XL1-Blue (*recA1*, *endA1*, *gyrA96*, *thi-1*, *hsdR17*, *supE44*, *relA1*, *lac*, [F9 proAB lacIq lacZΔM15, Tn10 (tetr)]) (Stratagene, Inc.) was used as the host for the plasmid construction. The *E. coli* strain BL21(DE3) (*fhuA2 [lon] omp Tgal (λDE3) [dcm] ΔhsdS λ DE3=λ sBamHIo ΔEcoRI-B int::(lacI::PlacUV5::T7gene1) i21 Δnin5*) was used as the host for protein expression.

The gene encoding SARS-CoV-2 M^pro^ (ORF1ab polyprotein residues 3264 to 3569, GenBank code: MN908947.3) was codon-optimized for *E. coli* expression and synthesized by GenScript Biotech. The synthesized gene was amplified by PCR using the forward primer 5′ - GGT​GGC​TCA​TAT​GTC​GGC​AGT​GCT​GCA​ATC​CGG​C TTTCGCAAAATGGC - 3′ and reverse primer 5′- GCC​ACC​TGG​ATC​CTT​AAT​GAT​GAT GAT​GAT​GAT​GGG​GAC​CCT​GG AAGGTTACACCAGAG - 3′ to introduce the N-terminal M^pro^ cleavage site (SAVLQ↓SGFRK; arrow indicates the cleavage site) and the C-terminal PreScission cleavage site (SGVTFQ↓GP) followed by a 6xHis-tag as previously described (24). The PCR fragment was then inserted into the pSumo plasmid using NdeI and BamHI restriction sites to generate an N-terminal Sumo-M^pro^ fusion construct pSUMO-SARS-CoV-2-M^pro^. The DNA sequences of the plasmids with the corresponding gene insertion were confirmed by Sanger sequencing (Eurofins).

### Protein Expression and Purification.

SARS-CoV-2 M^pro^ with a C-terminal 6xHis-tag was expressed and purified from the *E. coli* BL21(DE3) strain. *E. coli* cells were cultured in lysogeny broth medium at 37 °C to an optical density at 600 nm absorbance of 0.8 and protein overexpression was induced by with 0.4 mM isopropyl β-d-1-thiogalactopyranoside for 20 h at 20 °C. The N-terminal Sumo-fusion was auto-cleaved while active M^pro^ was expressed, generating authentic N termini of M^pro^. The next day cells were harvested by low-speed centrifugation and resuspended in binding buffer (25 mM Tris⋅HCl pH = 8.0, 150 mM NaCl, 5 mM β-mercaptoethanol, 20 mM imidazole, and Xpert protease inhibitor mixture [GenDEPOT]). Sonication was used to disrupt cells and cell debris were removed by centrifugation at 10,000 × *g* for 10 min at 4 °C. The supernatant was mixed with binding buffer preequilibrated Ni^2+^ Sepharose 6 Fast Flow resin (GE Healthcare) for 30 min at 4 °C and then loaded onto the column. After washing, protein was eluted by adding buffer A (25 mM Tris⋅HCl, pH 8.0, 150 mM NaCl, 5 mM β-mercaptoethanol, and Xpert protease inhibitor mixture) supplemented with 40, 60, 80, and 100 mM imidazole, respectively. The purity of M^pro^ in each fraction was visualized by sodium dodecyl sulfate polyacrylamide gel electrophoresis (SDS/PAGE) followed by Coomassie Brilliant Blue (CBB) staining. Protein fractions containing >95% pure M^pro^ were pooled and buffer-exchanged with buffer B (20 mM Tris⋅HCl, pH 7.3, 150 mM NaCl, 1 mM ethylenediaminetetraacetic acid [EDTA], and 1 mM dithiothreitol [DTT]) using a Amicon Ultra-15 Centrifugal Filter Unit (MilliporeSigma). Purified M^pro^ was then mixed with PreScission protease (GenScript Biotech) at a ratio of 1 mg protein to 10 units protease based on manufacturer recommendations and incubated for 20 h at 4 °C, resulting in M^pro^ with authentic N and C termini. GST Bind Resin (Novagen) and Ni^2+^ Sepharose 6 Fast Flow resin were applied sequentially to remove GST-tagged PreScission protease and remaining M^pro^-His. To further eliminate protein contamination from the PreScission protease, uncleaved M^pro^-His and 6xHis-tag, M^pro^ was concentrated and loaded on to a Superdex 75 increase 10/300GL size-exclusion column (GE Healthcare) preequilibrated with buffer B. The elution profile of M^pro^ protein was visualized by SDS/PAGE followed by CBB staining and pure M^pro^ protein was combined for further use.

### DEC-Tec Affinity Selection with M^pro^.

To identify M^pro^ binding compounds we screened our DEC-Tec library pool in two tubes: 1) absence of M^pro^ protein (bead binding no-target control, NTC) and 2) presence of His-M^pro^ at 1 µM. Before the screen was initiated, our DEC-Tec libraries were quantitated using qPCR and pooled together such that 1 million copies of each compound were present. Three rounds of DEC-Tec selection were been performed to improve ligand enrichment. The DNA barcode from the last round of selection was PCR-amplified and sequenced to identify the linked drug-like binders. In brief, DEC-Tec screen and sequencing were performed as follows: 1) His-M^pro^ at 1 µM was incubated with DEC-Tec libraries in 50 mM Tris⋅HCl buffer, pH 8.0, containing 150 mM NaCl, 10 mM imidazole, 1 mM TCEP, 1 mM CHAPS, and 0.1 mg/mL sheared salmon sperm DNA for 45 min at room temperature (RT) with continuous shaking; 2) His-M^pro^ along with binding molecules were immobilized by HisPur Ni-NTA magnetic beads through brief vortex; 3) beads were washed one time with the aforementioned selection buffer without the addition of sheared salmon sperm DNA by brief and vigorous vortex; 4) binding DEC-Tec molecules were dissociated form His-M^pro^ by heating beads at 80 °C for 10 min with continuous shaking; 5) the resulting eluent containing protein-binding molecules was further incubated with fresh His-M^pro^ to initiate another round of selection following the same protocol described above; 6) after the last round of selection, the eluted encoding oligonucleotides were amplified using Platinum Taq DNA Polymerase High Fidelity using primers that incorporate complementary sequences to the library headpiece or tailpiece along with the Illumina READ 1 or READ 2 sequences required for clustering and Illumina sequencing; 7) the amplified DNA were cleaned by Agencourt AMPure XP beads and quantitated by Agilent high-sensitivity DNA kit using a Bioanalyzer; 8) DNA was then denatured and sequenced along with a 3% PhiX spike-in in a single-read, 105-cycle sequencing on an Illumina NextSEq. 500 instrument; and 9) the FASTQ format sequencing data were generated through Illumina BaseSpace and decoded and analyzed.

### Enzyme Inhibition Assay and *K*_i_ Values Determination.

To evaluate the potency of synthesized compounds against M^pro^ we first measured the proteolytic activity of 50 nM M^pro^-His and M^pro^ in the presence and absence of 25 µM compound using the fluorescent peptide Dabcyl-KTSAVLQSGFRKM-E(Edans)-NH_2_ (GenScript Biotech) as the reporter substrate at a concentration of 15 µM. Compounds were incubated with M^pro^ for 20 min at RT in reaction buffer composed of 20 mM Tris⋅HCl, pH 7.3, 100 mM NaCl, 1 mM EDTA, 1 mM DTT, and 0.02% Tween-20. Hydrolysis of the fluorescent peptide was monitored at an emission wavelength of 460 nm with excitation wavelength at 360 nm, using a TECAN M200 plate reader. Compounds that inhibited M^pro^ activity by less than 90% were considered inactive (*SI Appendix*, Table S1).

To determine the *K*_i_ values of active compounds, 25 nM M^pro^ was mixed with increasing concentrations of compounds (from 4 nM to 4,000 nM with twofold dilutions) and hydrolysis of 15 µM fluorescent peptide was monitored. Initial hydrolysis rates of fluorescent peptide were plotted as a function of compound concentrations and *K*_i_ values were obtained by fitting the data into the Morrison equation (42) with SE from triplicates. The Michealis–Menten constant of enzyme (*K*_m_) value used for *K*_i_ calculations is 17 µM (43).

The Morrison equation used was

$$Y=Vo*\;(1-(\left(\left(\left(E_{t}+X+(1+\left(S/K_{\text{m}}\right)\right)\right)\right)\;-\;{\left(\left(\left(E_{t}+X+(K_{\text{i}}*(1+\left(S/K_{\text{m}}\right)\right)\right)\right)}^{2}-\;{4*E_{t}*X)}^{0}.5))/\left(2*E_{t}))\right)$$

where *Y* is enzyme activity, *X* is concentration of inhibitor, *E*_*t*_ is enzyme concentration, and *S* is substrate concentration.

### Thermal Shift Assay.

The dye SYPRO Orange (Thermo Fisher Scientific) was used to perform the protein thermal shift assay. The assay was set up on a 384-well Roche plate where the SARS-CoV-2 main protease at a concentration of 1.5 μM was incubated with the test compound at various concentrations and SYPRO Orange dye at 5× in a 10 μL reaction. The melting curve experiment and data analysis were run on a Roche Lightcycler 480 real-time PCR instrument.

### Human Protease Assays.

Cathepsin B assay: A kit from BPS Bioscience (79590) was used with adjustments to the manufacturer’s protocol as follows. The activated enzyme was diluted 250-fold just before use, and the reaction was run at RT in black 96 half-area plates in a total reaction volume of 50 μL containing 0.01% Tween-20. Instead of measuring endpoints at 60 min the increase in fluorescence (excitation [Ex] 360 nm, emission [Em] 460 nm) per second (RFU/s) was obtained from linear progress curves over 10 min. Fractional activities were calculated from reactions with no inhibitors. E-64 was used as a positive control.

Thrombin assay: A kit from Sigma-Aldrich (MAK243) was used with adjustments to the manufacturer’s protocol as follows. Thrombin was reconstituted according to protocol and then diluted 10-fold with assay buffer just before use. The unknown AMC-peptide substrate was further diluted threefold with the provided buffer. The reaction was run at RT in kinetic mode for 10 min in white 96 half-area plates in a total reaction volume of 100 μL containing 0.01% Tween-20. The increase in fluorescence (Ex 350 nm, Em 450 nm) per second (RFU/s) was obtained from linear progress curves. Fractional activities were calculated from reactions with no inhibitors. CDD-1472, a potent thrombin inhibitor identified by our group using DEC-Tec (6), was used as a positive control.

Renin assay: A kit from BPS Bioscience (80211) was used with adjustments to the manufacturer’s protocol as follows. The reaction was run at RT in black 96 half-area plates in a total reaction volume of 50 μL containing 0.01% Tween-20. Instead of measuring endpoints at 30 min the increase in fluorescence (Ex 490 nm, Em 520 nm) per second (RFU/s) was obtained from linear progress curves over 10 min. Fractional activities were calculated from reactions with no inhibitors. Aliskiren was used as a positive control.

MMP-1 assay: A kit from Biovision (K794-100) was used with adjustments to the manufacturer’s protocol as follows. The enzyme was reconstituted according to protocol and then diluted fivefold with assay buffer just before use. The unknown fluorogenic substrate was further diluted 10-fold with the provided assay buffer. The reaction was run at 37 °C in black 96 half-area plates in a total reaction volume of 50 μL containing 0.01% Tween-20. The increase in fluorescence (Ex 490 nm, Em 520 nm) per second (RFU/s) was obtained from linear progress curves over 10 min. Fractional activities were calculated from reactions with no inhibitors. GM 6001 was used as a positive control (44).

### Crystallography and Data Collection.

To obtain the structure of M^pro^ in complex with CDD-1713, M^pro^ and CDD-1713 were mixed at a 1:2 molar ratio and incubated at 4 °C overnight to facilitate complex formation. Crystal screening was performed using commercially available crystal screening suites PEGs, PEGII, PACT, and JCSGI from Qiagen in 96-well format. Hanging drops were set up by an in-house TTP LabTech Mosquito instrument and crystals were obtained through the vapor diffusion method. Crystals were obtained in a condition of 20% (wt/vol) PEG3350 and 0.2 M sodium acetate at RT and 25% glycerol was used as the cryoprotectant.

X-ray diffraction data were collected at the Berkeley Center for Structural Biology using the Advanced Light Source synchrotron beam line. Reflection data were indexed, integrated, and scaled using the iMosflm and the CCP4i Suite (45). Molecular replacement was performed using the M^pro^ structure (Protein Data Bank ID code 7K3T) as the search model. Structures were further refined several rounds using PHENIX.refine and Crystallography Object-Oriented Toolkit (Coot) (46). The data collection and refinement statistics are listed in *SI Appendix*, Table S3. The UCSF Chimera program was used to generate Fig. 2. The electron densities of CDD-1713 and the covalent bond between residue M^pro^ Cys145 and CDD-1713 in the crystal structure were further examined by creating a polder mFo-Fc OMIT map using the PHENIX software.

### Metabolic Stability Assay in Liver Microsomes.

Compounds CDD-1713 and CDD-1976 (2.0 μM) were incubated in mouse or human liver microsomes (0.5 mg protein/mL) at 37 °C. The samples were collected at specific time points, 0, 30, and 60 min, in duplicate. The reactions were terminated by adding an equivalent volume of ice-cold methanol and vortexed. The reaction mixtures were centrifuged at rcf 15,000 for 15 min. Three microliters L of the supernatant was analyzed by UHPLC-Q Exactive Orbitrap MS (Thermo Fisher Scientific) equipped with 50-mm × 4.6-mm column (XDB C-18; Agilent Technologies). The column temperature was maintained at 40 °C. The flow rate was at 0.3 mL/min with a 30% mobile phase (acetonitrile containing 0.1% formic acid). Q Exactive MS was operated in positive mode with electrospray ionization. Ultrapure nitrogen was applied as the sheath (45 arbitrary units), auxiliary (10 arbitrary units), sweep (1.0 arbitrary unit), and the collision gas. The capillary gas temperature was set at 275 °C and the capillary voltage was set at 3.7 kV. Mass Spectrometry (MS) data were acquired from 80 to 1,200 Da in profile mode. JQ1 and alprazolam were used as the short and long half-life control, respectively.

### Cell Uptake Assay and Cytotoxicity Assay in HepG2.

The HepG2 cells maintained in DMEM (containing 1 g/L glucose, 10% fetal bovine serum [FBS], and 1% penicillin/streptomycin) were seeded in a 12-well plate (a final density of 5 × 10^5^ per well) for cell uptake assay and 96-well plate for cytotoxicity assay (a final density of 5 × 10^4^ per well). The plate was incubated at 37 °C for 24 h.

### Cell Uptake.

The cells in 12-well plate were treated with CDD-1713 or CDD-1976 (final concentration: 10 μM). The plate was incubated at 37 °C for another 2 h. The medium in the plate was decanted and the cells were rinsed with 1 mL of 1× DPBS three times, then 0.5 mL of 0.25% trypsin was added to each well and incubated for 3.5 min (at 37 °C). One milliliter of complete medium was added to each well to quench the reaction. After spinning at 350 × *g* for 5 min, the cell pellet was reconstituted in MeOH/H_2_O (vol/vol 1/1) and CDD-1713 or CDD-1976 was extracted from 100 μL of the resulting mixture with 100 μL of ice-cold methanol. The mixtures were centrifuged at rcf 15,000 for 15 min. Three microliters of the supernatant was analyzed by UHPLC-Q Exactive Orbitrap MS (Thermo Fisher Scientific) equipped with a 50-mm × 4.6-mm column (XDB C-18; Agilent Technologies). The column temperature was set at 40 °C. Ultrapure nitrogen was applied as the sheath (45 arbitrary units), auxiliary (10 arbitrary units), sweep (1.0 arbitrary unit), and the collision gas. The capillary temperature was 275 °C, and the auxiliary gas temperature was 380 °C. The spray voltage was 3.75 kV. MS data were acquired from 80 to 1,200 Da in profile mode. The mobile phase system was (A) water (containing 0.1% formic acid) and (B) acetonitrile (containing 0.1% formic acid), with a flow rate of 0.3 mL/min. The gradient elution program was as follows: 0 to 0.25 min, 40% B; 0.25 to 1.5 min, 40 to 98% B; 1.5 to 3.5 min, 98% B; 3.5 to 3.8 min, 98 to 40% B; 3.8 to 5 min, 40% B. Doxorubicin and dacarbazine were used as positive and negative controls, respectively. The experiments were performed in triplicate.

### Cytotoxicity Assay.

The cells in 96-well plate were treated by CDD-1713 or CDD-1976 at the final concentrations of 0, 10, 20, 50, or 100 μM (*n* = 3 replicates per concentration level per drug). After 24-h incubation at 37 °C, the medium in the plate was decanted and 100 μL of Dulbecco’s modified Eagle’s medium (DMEM) (without phenol red) was added into each well. The cell variability was measured using XTT method. The absorbance at 475 nm was read with a Tecan M1000 pro machine, with a reference wavelength of 660 nm. The reading was normalized by vehicle with final 0.5% dimethyl sulfoxide (DMSO) for each drug.

### Plasma Stability Assay.

Compound CDD-1713 and CDD-1976 were incubated in human and mouse plasma, respectively, at a concentration of 10 µM in duplicate (*n* = 2) at 37 °C. At time points of 0, 30, 60, and 120 min, 15 µL of sample were taken out and the reactions terminated by adding 75 µL of ice-cold methanol. The reaction mixtures were then vortexed for 30 s and centrifuged at rcf 15,000 for 15 min. Following centrifugation, 3 μL of the supernatant was analyzed using UHPLC-Q Exactive Orbitrap MS. The percentage of test compound remaining at the individual time points relative to the 0-min sample was then determined.

### RTCA Assay for Evaluating In Vitro Inhibition of SARS-CoV-2 Replication by M^pro^ Inhibitors.

Inhibition of SARS-CoV-2 replication in VERO E6 and HEK hACE2 cells was measured using an xCELLigence RTCA HT Analyzer (Agilent Technologies), tracking the virus-induced cytopathic effect (CPE) on the cellular growth kinetics. Applied methods were similar to those recently described elsewhere (47). After a background reading of the 96-well E-plate using 50 μL only of media, 1 × 10^4^ cells were seeded in each well, using 100 μL of culture media (VERO E6 cells: DMEM + 1× penicillin/streptomycin + 10% FBS or HEK-hACE2 cells: DMEM + 1× penicillin/streptomycin + 10% FBS + 10 mM Hepes). The E-plate was incubated for 30 min at RT to minimize edging effects (48). After the cells settled, the E-plate was transferred to an xCELLigence instrument for real-time analysis of cell proliferation for 24 h. Drug candidates dissolved in DMSO were twofold serially diluted in cell culture media in a 96-deep-well plate. Drug samples were prepared in duplicate and DMSO was included as buffer control. An equal volume of cell culture media containing SARS-CoV-2 (USA_WA1/2020 isolate) was added to the drugs. Final concentrations of small drug tested ranged from 0.2 μM to 50 μM, and wells containing virus only or cell culture media only were added as controls. The plate was incubated for 1 h at 37 °C in 5% CO_2_. After the incubation, the E-plate was removed from the xCELLigence instrument and the cell culture media in the E-plate was replaced by 250 μL of the drugs candidates/virus mixture from the deep-well plate. The E-plate was then transferred back to the xCeLLigence instrument and real-time analysis of the CPE was continued for an additional 75 h.

Data were analyzed using the RTCA software (Agilent Technologies). Duplicate wells were averaged, and the cell index was normalized to the last measured time point before the addition of the drugs/virus mixture Using Prism9 software (GraphPad) the normalized cell index data were plotted versus time, and the IC_50_ of each drug candidate was calculated.

## Supplementary Material

Supplementary File

## Data Availability

All study data are included in the article and/or *SI Appendix*. The M^pro^ structure in complex with CDD-1713 has been deposited to the Protein Data Bank with PDB ID 7LTN.
