# Supplementary material for: DNA-encoded chemistry technology yields expedient access to SARS-CoV-2 Mpro inhibitors
Source: Proc Natl Acad Sci U S A. 2021 Aug 23;118(36):e2111172118. doi: 10.1073/pnas.2111172118 (PMC8433497; doi:10.1073/pnas.2111172118)
Supplement: Supplementary File [file pnas.2111172118.sapp.pdf]

Supplementary Information:

DNA-Encoded Chemistry Technology Yields Expedient Access to SARS-CoV-2 M^{pro} Inhibitors

Srinivas Chamakuri[1,#], Shuo Lu[2,#], Melek Nihan Ucisik[1,#], Kurt M. Bohren[1], Ying-Chu Chen[1], Huang-Chi Du[1], John C. Faver[1], Ravikumar Jimmidi[1], Feng Li[1,2], Jian-Yuan Li[1], Pranavanand Nyshadham[1], Stephen S. Palmer[1], Jeroen Pollet[4,5], Xuan Qin[1], Shannon E. Ronca[4], Banumathi Sankaran[6], Kiran L. Sharma[1], Zhi Tan[1], Leroy Versteeg[4,5], Zhifeng Yu[1], Martin M. Matzuk[1,2]*, Timothy Palzkill[2,3]*, Damian W. Young[1,2,3]*.

[1]Center for Drug Discovery, Department of Pathology & Immunology, Baylor College of Medicine, Houston, Texas 77030, United States of America

[2]Department of Pharmacology and Chemical Biology, Baylor College of Medicine, Houston, Texas 77030, United States of America

[3]Verna and Marrs McLean Department of Biochemistry and Molecular Biology, Baylor College of Medicine, Houston, Texas 77030, United States of America

[4]Department of Pediatrics, National School of Tropical Medicine, Baylor College of Medicine, Houston, Texas 77030, United States of America

[5]Center for Vaccine Development, Texas Children's Hospital, 1102 Bates Street, Houston, Texas 77030, United States of America

[6]Department of Molecular Biophysics and Integrated Bioimaging, Berkeley Center for Structural Biology, Lawrence Berkeley National Laboratory, Berkeley, CA 94720, USA

[#], equal first author

*, communicating authors, mmatzuk@bcm.edu or timothyp@bcm.edu or damian.young@bcm.edu

Table of Contents

Content	Page
Supplementary figures	2-10
DECL general methods, experimental procedures	11-17
Chemistry general methods, experimental procedures and NMR data	17-30
^{1}H, ^{13}C NMR spectra of all new compounds and HRMS data	31-86

Supplementary Figures:

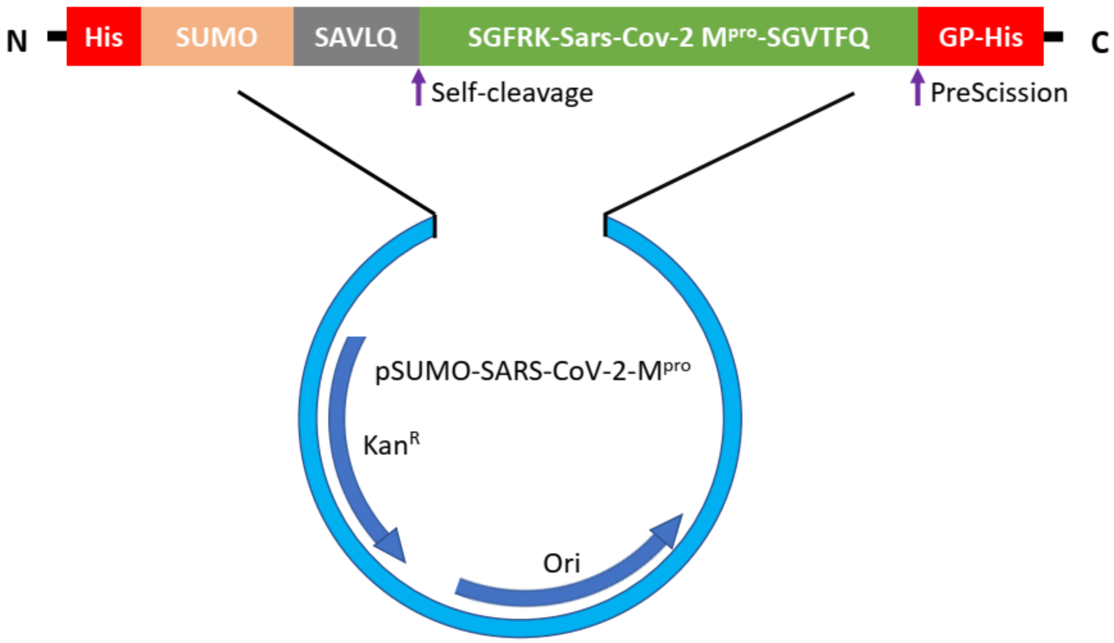

Figure S1. Schematic representation of pSUMO-SARS-CoV-2- M^{pro} construction. 6×His-tag SUMO was fused to the N-terminus of M^{pro} gene through M^{pro} cleavage-site (SAVLQ↓SGFRK; arrow indicates the cleavage site). PreScission cleavage site (SGVTFQ↓GP) followed by a 6×His-tag was introduced to the C-terminus of M^{pro}.

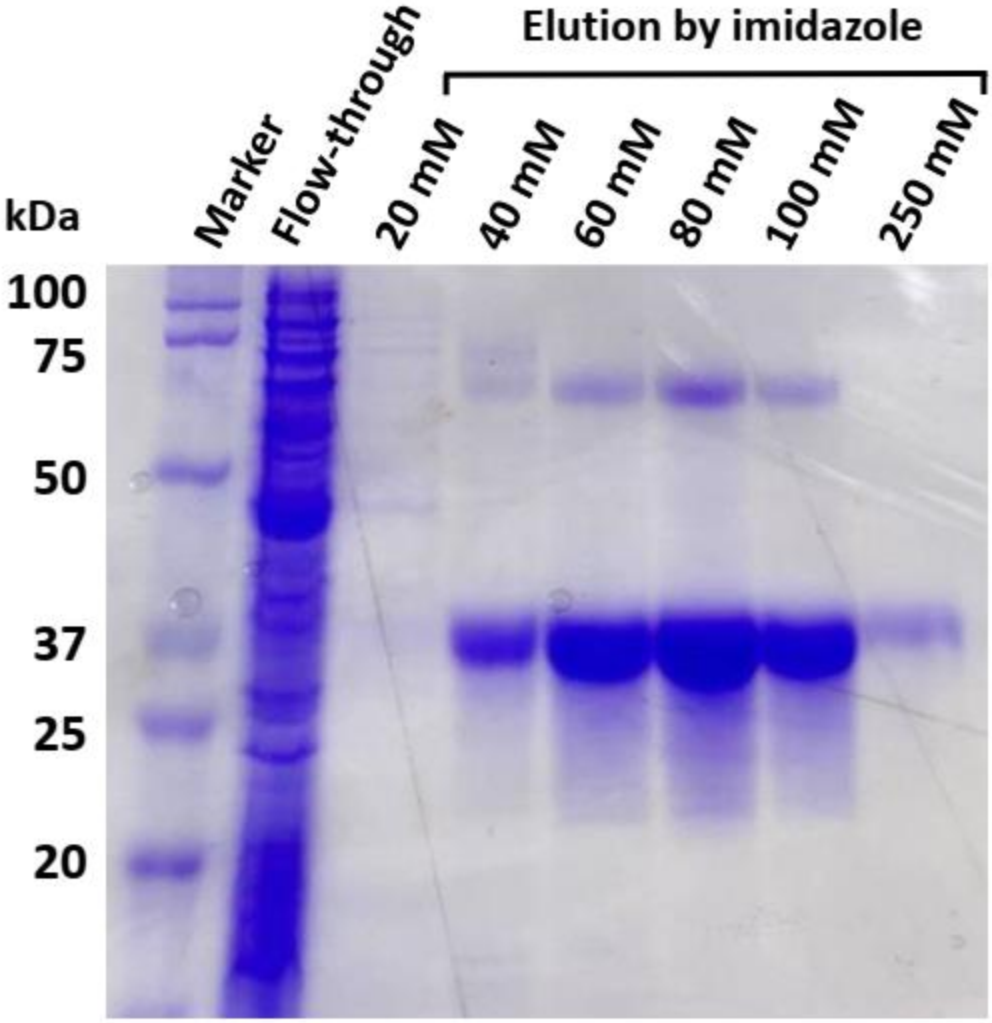

Figure S2. M^{pro} –His6 purification profile. Cell lysate was mixed with binding buffer pre-equilibrated Ni2+ Sepharose 6 Fast Flow resin and M^{pro}-His6 was eluted with increasing concentrations of imidazole (20 mM-250 mM) (see Materials and Methods). The elution profile of M^{pro} -His6 protein was visualized by SDS-PAGE followed by CBB staining. Two bands of M^{pro} -His6 on the gel are approximately 35 kDa (monomer) and 70 kDa (dimer).

A.

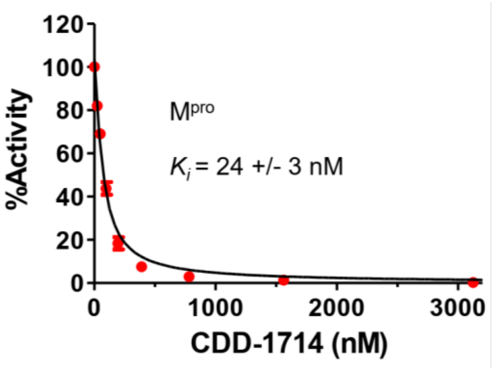

B.

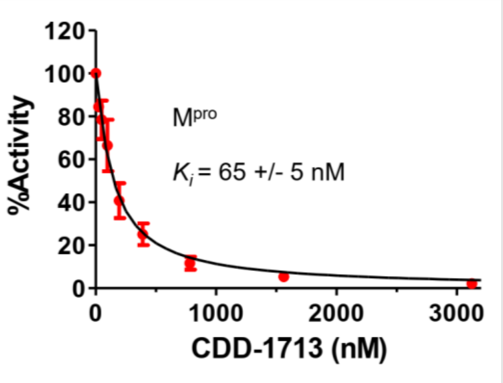

C.

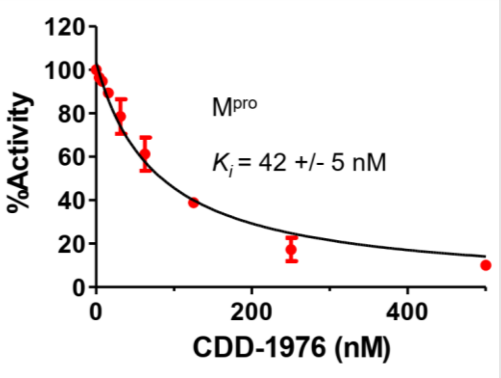

Figure S3. Inhibition Ki value determination against M^{pro}. **A**. Concentration-dependent inhibition curve of CDD-1714. 25 nM of M^{pro} cefotaxime was mixed with increasing concentrations of CDD-1714. The remaining activities (red dots) of M^{pro} towards fluorescent peptide were plotted as a function of compound concentrations and Ki values was calculated by fitting the data into Morrison equation with standard error from triplicates. **B**. Concentration-dependent inhibition curve of CDD-1713. **C**. Concentration-dependent inhibition curve of CDD-1976.

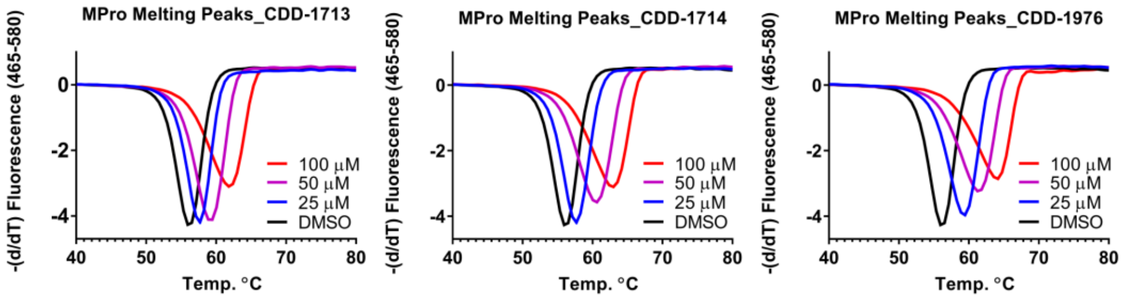

MPro melting temperature shift, ΔTm °C

Compound	25 µM	50 µM	100 µM
CDD-1713	1.45 ± 0.05	2.95 ± 0.14	5.29 ± 0.16
CDD-1714	1.48 ± 0.00	3.99 ± 0.26	6.15 ± 0.30
CDD-1976	3.03 ± 0.04	5.03 ± 0.04	7.46 ± 0.07

Figure S4. CDD-1713, CDD-1714, and CDD-1976 stabilized the SARS-CoV-2 main protease (M^{pro}) in the protein thermal shift stability assay. Data analysis and protein melting temperature (Tm) calculation were run on a Roche Lightcycler 480 real-time PCR instrument (n = 2). The plot was generated using a GraphPad Prism software.

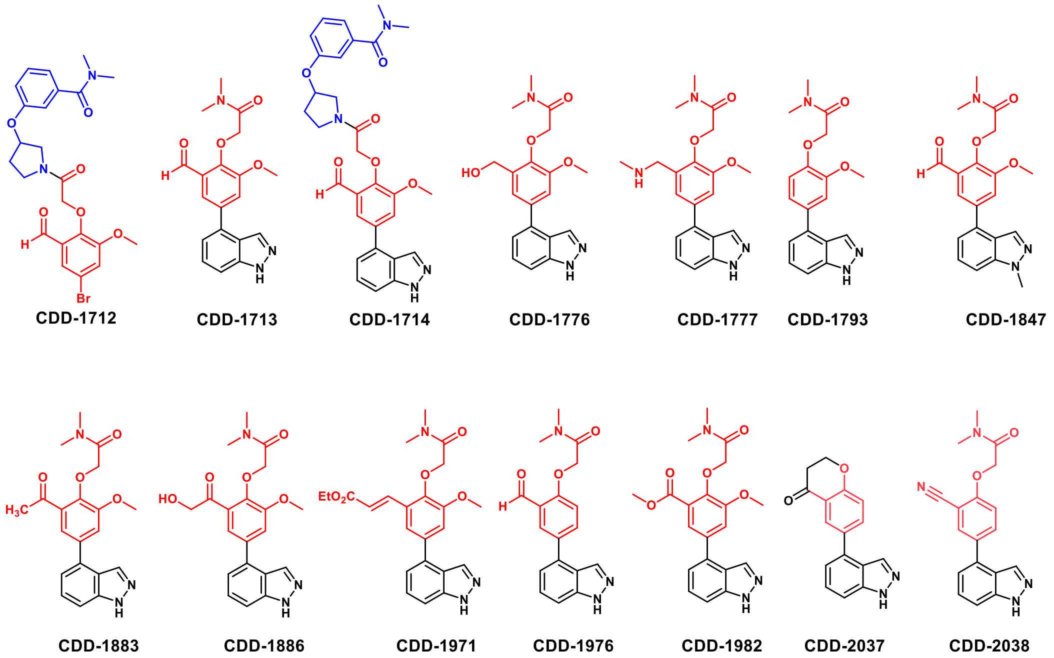

Figure S5. Synthesized CDD-1713 analogs for M^{pro}.

Compound	M^{pro}-His<br>K_i, nM	M^{pro}<br>K_i, nM
**CDD-1712**	Inactive[a]	Inactive
**CDD-1713**	45±3	65±5
**CDD-1714**	20±3	24±3
**CDD-1776**	Inactive	Inactive
**CDD-1777**	2200±140	2400±230
**CDD-1793**	Inactive	Inactive
**CDD-1847**	Inactive	Inactive
**CDD-1883**	Inactive	Inactive
**CDD-1886**	Inactive	Inactive
**CDD-1971**	Inactive	Inactive
**CDD-1976**	37±4	42±5
**CDD-1982**	Inactive	Inactive
**CDD-2037**	Inactive	Inactive
**CDD-2038**	Inactive	Inactive

Table S1. Enzymatic activity data, [a]Less than 50% inhibition observed with 25 µM compound added for initial screening; Inactive= Compounds that inhibited M^{pro} activity by less than 90% with 25 µM compound were considered inactive

**Timeline of DEL process**	
Protein expression and purification	1-2 weeks
DEL Selection against M^{pro} and Data analysis	1-2 weeks
Off DNA synthesis and validation	2-3 weeks
Antiviral testing	2 weeks

Table S2. Approximate time line of entire process from protein production to antiviral testing

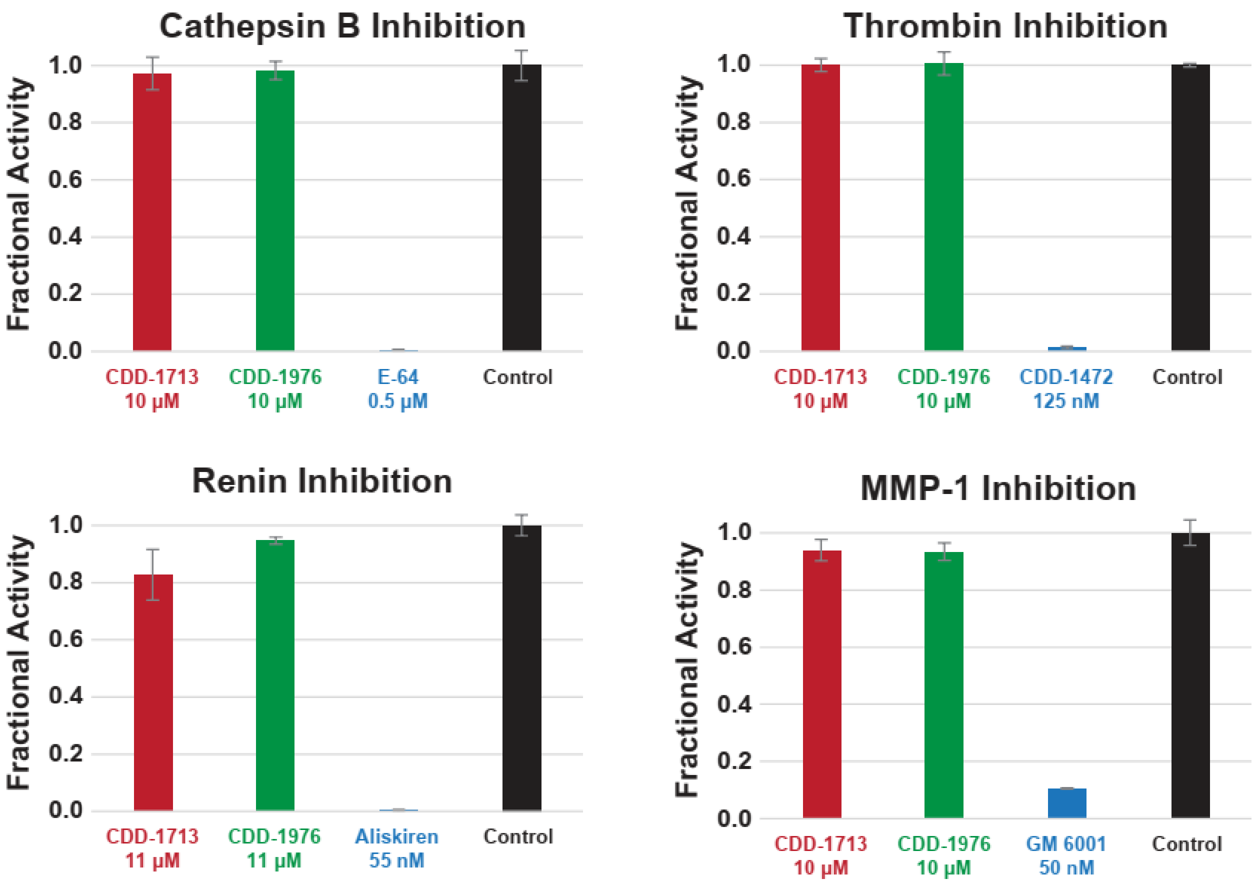

Figure S6. Potential off-target inhibition of major proteases, i.e. cathepsin B (a cysteine protease like M^{pro}), thrombin (a serine protease), renin (an aspartic protease), and matrix metallopeptidase 1 (MMP-1), was tested with all active compounds shown here with CDD-1713 and CDD-1976 and control inhibitors as indicated. The best inhibition was observed by CDD-1713 of renin with an estimated Kiapp of 53 uM, calculated as described in methods section.

Data collection	Mpro-CDD-1713
space group	C 1 2 1
a, *b*, *c* (Å)	114.74 53.71 44.49
α,β,γ (°)	90.00 101.16 90.00
resolution range (Å)	33.87 - 1.79 (1.85 - 1.79)
% completeness	99.4 (97.4)
R_{merge} (%)	13
I/σ (I) at 1.78Å)	1.23 (at 1.78Å)
Wilson B-factor (Å^2)	26.1
molecules per asymmetric unit	1
no. of unique reflections	25098(2482)
R_{work}/R_{free} (%)	19.8/24.5
no. of protein atoms	2373
average B-factor (Å^2)	30
protein	29.5
solvent	35.7
ligand	30.3
bond length (Å)	0.008
bond angles (deg)	1.09
PDB codes	7LTN

Table S3. Crystal structure data refinement statistics.

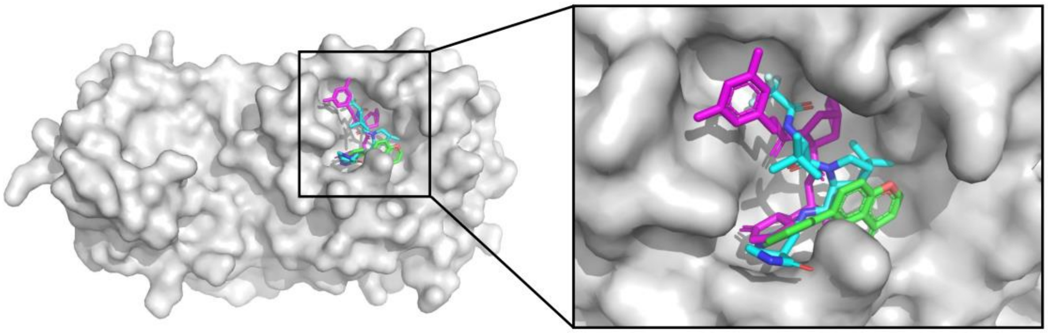

Figure 3E. Superposition of M^{pro}/CDD-1713 and M^{pro}/MI-23 crystal structures along with the docking pose of PF-07321332 shown on the left and a zoom-in view on the right. Surface: M^{pro} protein; Green sticks: CDD-1713; Magenta stick: MI-23; Cyan stick: PF-07321332

	Assay (half-life)	JQ1	Alprazolam	CDD-1713	CDD-1976
**1**	MLM $t_{1/2}$ (min.)	11.8	296	8.4	10.2
**2**	HLM $t_{1/2}$ (min.)	9.7	832	15.3	41.4

Table S4. Metabolic Stability of CDD-1713 and CDD-1976 in HLM and MLM. Final concentrations; Liver microsomes: 0.5 mg protein/ml, Compound concentration: 2.0 uM, NADPH Concentration: 1.0 mM; **JQ1:** short half-life Control, **Alprazolam:** long half-life control. In duplicate at 0, 30 and 60 min.

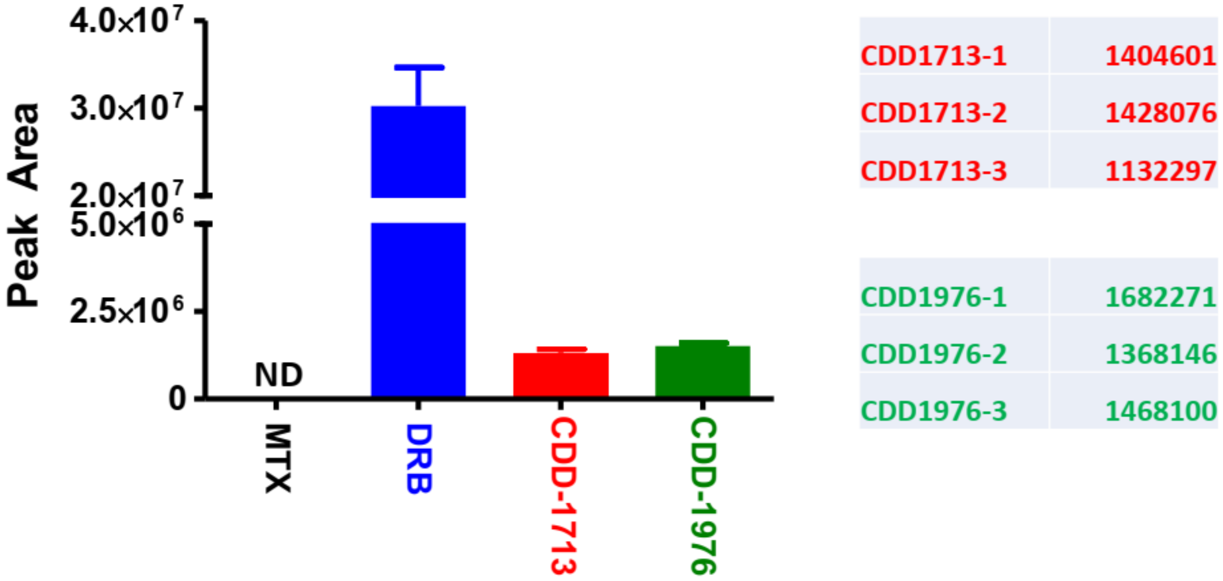

Figure S7A. HepG2 cell uptake of CDD-1713 and CDD-1976; The HepG2 cell uptake capacities of CDD-1713 and CDD-1976 were expressed as the intracellular concentrations (peak areas) of these two compounds. Methotrexate (MTX) and doxorubicin (DRB) were used as the negative and positive controls, respectively. The HepG2 cells were incubated with the compounds and controls (final concentration 10 μM) for 2 hours at 37 °C, harvested and homogenized. The intracellular concentrations were measured with UHPLC-Q Exactive Orbitrap MS. ND stands for "not detected".

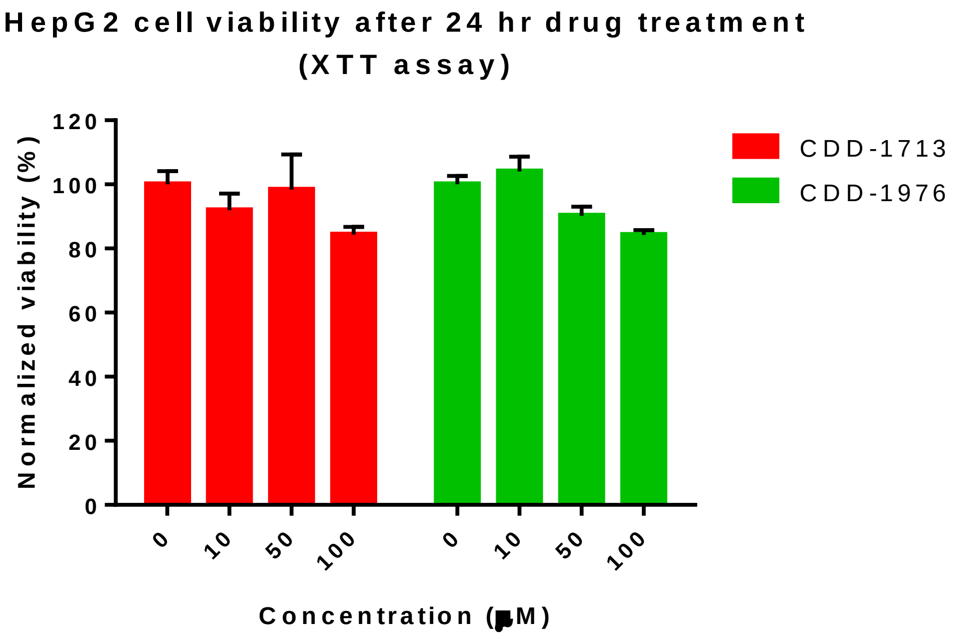

(IC$_{50}$: >100 µM)

Figure S7B. HepG2 cell viability after incubation with CDD-1713 and CDD-1976 for 24 hours; The HepG2 cells were incubated with CDD-1713 and CDD-1976 (0-100 µM) for 24 hours at 37 °C. Cell viability was measured with XTT assay. XTT readings were normalized by the control group (DMSO) to give the normalized viabilities. The IC$_{50}$ values were expressed as ">100 µM" for CDD-1713 and CDD-1976, as the cell viabilities were larger than 80% in the 100 µM group for both compounds.

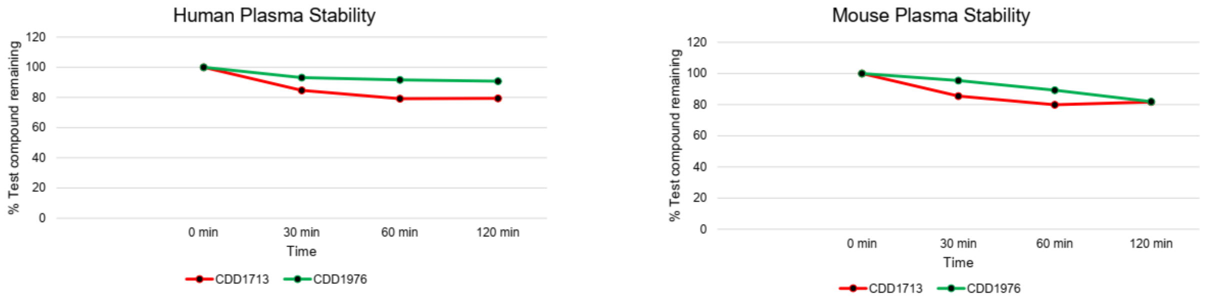

Figure S7C. Plasma stability of CDD-1713 and CDD-1976 in human and mouse plasma. CDD-1713 and CDD-1976 were incubated in human and mouse plasma respectively at a concentration of 10 µM in duplicate (n=2) at 37 °C. The reactions were terminated at time points of 0, 30, 60 and 120min by adding ice-cold methanol. Following centrifugation, the supernatant was analyzed using UHPLC-Q Exactive Orbitrap MS. The percentage of test compound remaining at the individual time points relative to the 0 min sample was determined.

General Information of DECL

The general materials, procedures and equipment utilized in this study referenced the related DECL work in our group reported previously[1,2,3,4,5,6,7,8] or other DECL publications.[9,10,11]

Materials and equipment used for the DNA-encoded chemical libraries. The starting unit dsDNA oligonucleotide with modified phosphates with PEG_4 linker and terminal amine (DEC-Tec Starting Unit/DTSU, **S1**) and encoding 5'-phosphorylated oligonucleotides were purchased from LGC Biosearch Technologies. A "spike-in" with 10-mer DNA oligonucleotide featuring a cholesterol tag and terminal amine was purchased from LGC Biosearch Technologies to charge with pooled library to assess chemical reaction progress. T4 DNA ligase in high concentration was obtained from Qiagen Enzymatics. DNAse/RNAse-free ultrapure water from Invitrogen, HPLC-grade acetonitrile from Fisher and high-purity absolute ethanol from Koptec were used to prepare buffer solutions. LC/MS-grade water from Fisher, Optima LC/MS-grade methanol from Fisher, hexafluoroisopropanol (99+% purity) from Sigma-Aldrich and HPLC-grade triethylamine from Fisher were used to prepare LC/MS running solvent. All listed buffer solutions were prepared in-house, including HEPES 10X ligation buffer (300 mM 2-[4-(2-hydroxyethyl)piperazin-1-yl]ethanesulfonic acid, 100 mM adenosine triphosphate, 100 mM dithiothreitol, 10 mM $MgCl_2$, aq. NaCl (5 M), aq. NaOH, and basic borate buffer (250 mM sodium borate/boric acid, pH 10). Chemical building blocks and reagents were purchased from various vendor sources and used without further purification. Building blocks were purchased from a variety of manufacturer and generally prepared in acetonitrile (MeCN), dimethyl sulfoxide (DMSO) or mixed aqueous acetonitrile. The stock solution of building blocks were stored in 2D barcoded tubes from Phenix with septa-caps from Phenix at -80 °C and aliquots were taken for each use. Solutions were transferred using Fisherbrand pipette tips. Polypropylene PCR tubes from Genemate, tubes from Eppendorf, 96-well PCR plates from ThermoFisher and 96-well deep-well plates from USA Scientific were used to perform chemical reactions or DECL production. Large volume of chemical reactions or ethanol precipitations were performed in polypropylene 15-mL, 50-mL centrifuge tubes or 250 mL screw-cap bottles from various manufacturers. Heated reactions were performed on Mastercycler nexus gradient from Eppendorf, benchtop heating blocks from ThermoFisher, or TS-DW deep well plate themoshaker from Grant, or laboratory ovens from Fisher. Solutions were centrifuged in 5424R centrifuge from Eppendorf, or Lynx 4000 centrifuges from ThermoFisher. Optical density measurements were made using a Biophotometer from Eppendorf. A Vanquish UHPLC system was integrated with LTQ XL ion trap mass spectrometer (ThermoFisher Scientific) for LC/MS analysis of DNA oligonucleotides. DNA ligation was assessed by gel electrophoresis

analysis and visualized with Molecular Imager Gel Doc XR system from BIO-RAD after staining in an ethidium bromide solution.

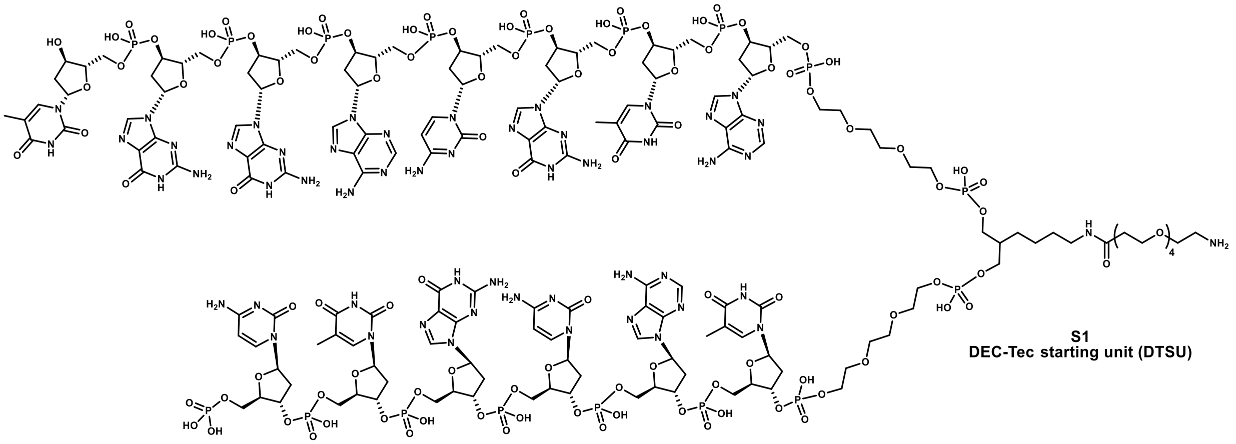

Structure of DTSU **S1** (5'-Phos-CTGCAT-Spacer 9-Amino C7 plus AOP-Spacer 9-ATGCAGGT 3').

General procedure for the analysis of DNA oligonucleotides. DNA sample or reaction mixture were diluted to 10 μM final concentration and injected in amounts of 5–10 μL on a Vanquish/LTQ system.

LC/MS Parameters for Thermo Vanquish UHPLC with LTQ Ion Trap MS Instrument

(i) LC settings

Column: Thermo DNAPac RP (2.1 x 50 mm, 4 μm)

Solvent A: 15mM triethylamine (TEA)/100mM hexafluoroisopropanol (HFIP) in water

Solvent B: 15mM TEA/100mM HFIP in 50% methanol

Solvent C: Methanol

Flow rate: 0.65 mL/min

Run time: 2 mins (gradient)

Column temperature: 100 °C (post column cooler at 40 °C)

Eluent: 15 mM TEA/100 mM HFIP in a water/methanol solvent system

(ii) MS settings

Source: ESI in negative mode

Spray voltage: 4100 V

Source heater temperature: 390 °C

Sheath Gas: 28 (instrument units)
Auxiliary Gas: 8 (instrument units)
Sweep Gas: 2 (instrument units)
Capillary temperature: 350 °C
Capillary voltage: -33.0 V
Tube lens: -92.0 V
MS Scan: 500 – 2000 *m/z*

DNA samples were analyzed on a Thermo Vanquish UHPLC system coupled to an electrospray LTQ ion trap mass spectrometer. An oligonucleotide column (Thermo DNAPac RP, 2.1 x 50 mm, 4 μm) was used with ion-pairing mobile phase for all the separations. Full scan negative-ion mode over the m/z range of 500–2000 was acquired for mass spectra. Data analysis was performed by processing the raw data with the automated biomolecule deconvolution with Promass and reporting software using ZNova novel algorithm to produce artifact-free mass spectra.

General procedures

General procedure for DNA ligation (Preparation of headpiece S2 from DTSU S1)

To DTSU (100 nmol, 100 μL, 1.0 equiv) was added primer_foward (5'-ACACTTGCTGGT-3', 105 nmol, 105 μL, 1.05 equiv), primer_reverse (5'-CAGCAAGTGTGA-3', 105 nmol, 105 μL, 1.05 equiv), and nuclease-free water (62.9 μL), followed by the addition of 10X HEPES buffer (41.7 μL) and T4 DNA ligase (2.1 μL) to make the final concentration of DNA 0.24 mM. Incubate the reaction mixture at room temperature overnight. The ligation progress was monitored with LC/MS analysis and gel electrophoresis. Gel electrophoresis was executed with a 12-well 10% TBE acrylamide gel from Invitrogen in 1X TBE buffer prepared in-house. The DNA loading sample was prepared by adding 10 μL of the diluted DNA sample and 2 μL of 6X DNA loading dye to a final concentration of 12 ng/μL. Gels were run at 120 V for 45 min and stained with 0.5 ng/mL ethidium bromide in 1X TBE buffer for 40 min before visualizing. After completion of the ligation, the reaction mixture was purified by ethanol precipitation to yield the headpiece **S2** for further use in chemical validation and library production. The ligation provides a final DNA sequence 5' d TAT GAT ACT AAA GTA AGT CAC ACA CAA TTG GAG CAG TCC TGA GTG AAT ACC TGC AT - Spacer 9-Amino C7- Spacer 9-ATG CAG GTA TTC ACT GAG GAC TGC TCC AAT TGT GTG TGA CTT ACT TTA GTA TCA TAT C 3'.

General procedure for ethanol precipitation and DNA reconstitution. Mixtures from chemical reactions or ligation were added 4% *v/v* of 5 M NaCl solution and 3 times of the reaction volume of absolute ethanol to crash out the DNA material. The mixture was pipet mixed thoroughly before storing at −20 °C overnight. The slurry was then centrifuged at 4000 x G for an hour, removed the supernatant and added another portion of pre-chilled 75% ethanol solution to wash the pellet. The pellet was centrifuged at 4000 x G for another hour before decanting the supernatant. The DNA pellet was air dried and nuclease-free water was added to reconstitute the DNA material. In general, ethanol precipitation was carries out after each chemical reactions or ligations and multiple 75% ethanol wash can be applied while needed.

General procedures for chemical reactions used in library production

Acylation: To a solution of on-DNA amine (51.4 μL, 0.7 mM in water) in 72 μL of pH 9.5 borate buffer (250 mM in water, 500 equivalents) was added 18 μL of carboxylic acid solution (200 mM in MeCN, 100 equivalents) and 18 μL of 4-(4,6-dimethoxy-1,3,5-triazin-2-yl)-4-methylmorpholinium chloride (DMTMM, 200 mM in water, 100 equivalents). The reaction was incubated at room temperature for 18 h and then quenched by ethanol precipitation.

Reductive Alkylation: An aldehyde building block (18 μL, 200 mM in MeCN, 100 equivalents) was added to a solution of DNA-conjugated amine (27.7 μL, 1.3 mM in water, 1 equivalent) in 36 μL of pH 5.8 MES buffer (2-(*N*-morpholino)ethanesulfonic acid, 500 mM in water, 500 equivalents). The mixture was added a solution of $NaCNBH_3$ (18 μL, 200 mM in water, 200 equivalents) and 36 μL MeCN to give final 40% *v/v* MeCN. The reaction mixture was heated at 40 °C for 16 hours, followed by being quenched by ethanol precipitation.

Reverse acylation: To a solution of on-DNA carboxylic acid (40.5 μL, 0.9 mM in water) in 72 μL of pH 5.8 MES buffer (250 mM in water, 500 equivalents) was added 18 μL of amine solution (200 mM in MeCN, 100 equivalents), 18 μL of 4-(4,6-dimethoxy-1,3,5-triazin-2-yl)-4-methylmorpholinium chloride (DMTMM, 200 mM in water, 100 equivalents) and 36 μL MeCN to give final 40% *v/v* MeCN. The reaction was incubated at room temperature for 18 h and then quenched by ethanol precipitation.

Nitro Reduction (hypodiboric acid): To a solution of nitro-containing DNA conjugate (50 μL, 0.7 mM in water) was added 22 μL of NaOH solution (818 mM in water, 500 equivalents), and ethanol (54 μL), followed by the addition of 54 μL of $B_2(OH)_4$ (100 mM in water, 150 equivalents). The reaction mixture was incubated at room temperature for 2 h prior to ethanol precipitation. Second

ethanol precipitation was performed after reconstitution the DNA pellet with water to remove residual building blocks. The solution of $B_2(OH)_4$ in neutral water was prepared freshly from vortexing or brief sonication before use.

Nucleophilic Aromatic Substitution (heating): To a solution of on-DNA amine (29.1 μL, 1.0 mM in water) in 58.2 μL of pH 9.5 borate buffer (250 mM in water, 500 equivalents) was added 5 μL of aryl halide (200 mM in MeCN, 100 equivalents) and 32 μL MeCN to give final 32% *v/v* MeCN. The reaction was heated at 40 °C for 18 h before being quenched by EtOH precipitation.

Nucleophilic Aromatic Substitution (DABCO): To a solution of on-DNA amine (29.1 μL, 1.0 mM in water) in 5.8 μL of sodium hydroxide solution (5000 mM in water, 1000 equivalents), 64 μL of water and 1.5 μL of MeCN was added 43.7 μL of aryl halide (200 mM in MeCN, 300 equivalents), followed by 1.5 μL of DABCO (1,4-diazabicyclo[2,2,2]octane, 100 mM in MeCN, 5 equivalents) to give final 32% *v/v* MeCN. The reaction was incubated at room temperature for 18 h and then quenched by ethanol precipitation.

Suzuki coupling: To the halogenated DNA conjugate (6.8 μL, 0.7 mM in water) was added 5 μL of CsOH (400 mM in water, 400 equivalents) and 2.5 μL of boronic acid (200 mM in 1,4-dioxane:water 1:1, 100 equiv), followed by adding 2 μL of freshly prepared sSPhos-Pd-G2 (5 mM in DMA, 2 equivalents). The reaction mixture was heated at 80 °C for 20 min and was cooled to room temperature. Sodium cysteine was added (2.5 μL, 200 mM in water, 100 equivalents) and heated at 80 °C for 20 min. The reaction mixture was cooled to room temperature prior to ethanol precipitation.

Sonogashira coupling: To the halogenated DNA conjugate (6.8 μL, 0.7 mM in water) was added 5 μL of CsOH (1000 mM in water, 1000 equivalents) and 2.5 μL of terminal alkyne (200 mM in DMA, 100 equiv), followed by adding 4 μL of freshly prepared sSPhos-Pd-G2 (5 mM in DMA, 4 equivalents). The reaction mixture was heated at 80 °C for 20 min and was cooled to room temperature. Sodium cysteine was added (2.5 μL, 200 mM in water, 100 equivalents) and heated at 80 °C for 20 min. The reaction mixture was cooled to room temperature prior to ethanol precipitation.

Synthesis of a DNA-Encoded Chemical Library (DECL)

Architecture of the main library build and Building block diversity analysis. Similar strategy for the two aspects were adopted from previous reported literature.

Synthetic sequence of the library build.

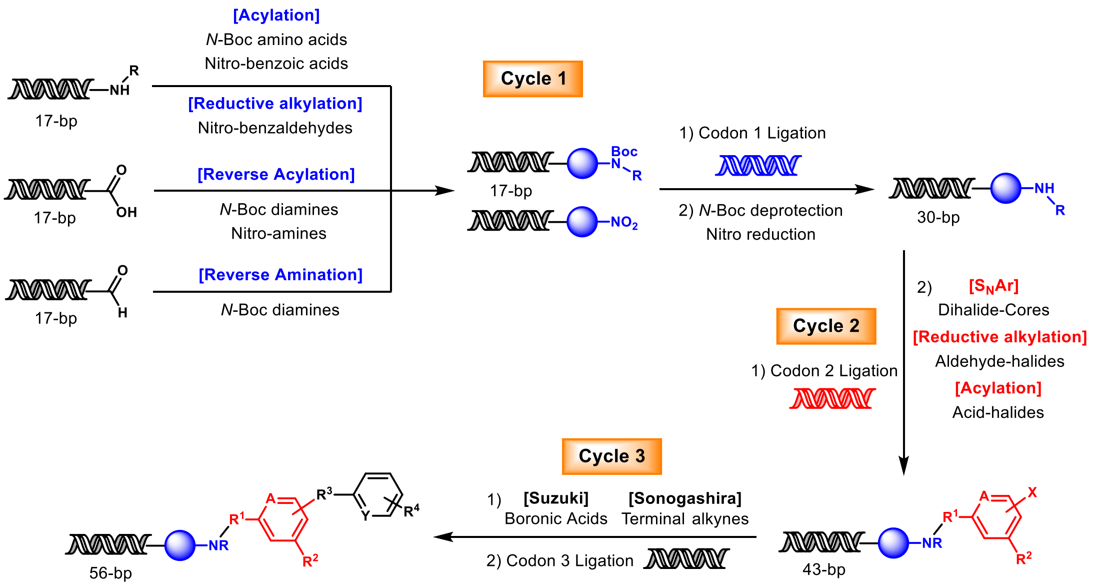

Scheme S1. Synthetic scheme of qDOS28_1. Cycle 1 is shown in blue, cycle 2 is shown in red and cycle 3 is shown in black. This library composed of 925 combinations in cycle 1, 585 building block in cycle 2 and 685 building blocks in cycle 3, which resulted in a library size of 370.7 million.

Procedure for Cycle 1. Various linker-functionalized **S2** (36 nmol/well) were plated individually into wells in 96-well plates. *N*-Boc amino acids (237) and nitro benzoic acids (44) were used for acylation with two different amine-terminating DNA substrates. Nitro benzaldehydes (42) were attached through reductive alkylation onto two different amine-terminating DNA substrates. *N*-Boc diamines (269) and nitro amines (46) were attached by reverse acylation to two different carboxylic acid terminated DNA substrates. *N*-Boc secondary diamines (177) were attached *via* reductive amination to one aldehyde terminated DNA substrate. Blanks were included as controls of building block-free and/or reagent-free conditions. Each well was carefully analyzed by LC-MS and 925 wells were proceeded to further step after removing those with low reaction conversions. After ethanol precipitation, each well was encoded with enzymatic ligation to unique DNA oligonucleotides (codon 1, 13-mer duplexed). Finally, *N*-Boc deprotection or nitro reduction was applied to the corresponding wells before pooling all wells. After pooling, additional ethanol precipitation was conducted to yield approximately 35.7 μmol of cycle 1 library pool.

Procedure for Cycle 2. After splitting the cycle 1 pool into 1178 wells (29.1 nmol/well), each well underwent codon 2 ligation with the general procedure. A variety of carboxylate aryl halides (380) were used for acylation with DEPBT and DMTMM methods; a variety of aryl dihalides (114) were attached by nucleophilic aromatic substitution using both heating and DABCO-promoted

methods; a variety of aldehyde aryl halides (95) was used for reductive amination using both $NaCNBH_3$ and $NaBH_4$ methods. The conditions of these methods are described in previous sections and all methods were encoded separately. In addition, blanks were included with the same reaction conditions in the absence of building blocks or reagents. After pooling and additional ethanol precipitation, approximately 31.5 μmol of the cycle 2 pool was recovered.

Procedure for Cycle 3. After splitting a portion of the cycle 2 pool into 691 wells (5 nmol/well), a series of boronic acids and pinacol esters (497) and terminal alkynes (188) were attached by Suzuki coupling and Sonogashira coupling. In addition, several blanks were included without building block and/or reagents to incorporate possible side-products. After ethanol precipitation, unique DNA oligonucleotides were used to encode each wells (codon 3). Each well was carefully analyzed by LC-MS to ensure the codon 3 ligation for further deconding process. After pooling and additional ethanol precipitation, approximately 3.4 μmol of the cycle 3 pool was recovered (71% recovery yield after 3 cycles of chemical transformations and ligations). Residual solids after Suzuki coupling and Sonogashira coupling need to be removed by centrifugation for further ligation to be proceeded successfully.

Preparation of amplifiable DECL samples for further selection experiments. After completion of the main library builds, the entire library material was ligated with a duplexed pair of 12-mer DNA oligonucleotides (library ID) to encode the overall library construct. After ethanol precipitation, the DECL material underwent sequential ligation with DNA oligonucleotides, containing a region to encode selection experiment, a degenerate region as molecular identifier during amplification, and a reverse primer region for post-selection PCR amplification (the purposes/design of these components are discussed in our previous publication).

General Methods for off DNA Synthesis:

All starting materials and reagents were purchased from commercial sources and used without further purification. Solvents were purchased as either anhydrous grade products in sealed containers or reagent grade and used as received. All reactions were carried out in dry glassware under a nitrogen atmosphere using standard disposable or gastight syringes, disposable or stainless steel needles, and septa. Stirring was achieved with magnetic stir bars. Flash column chromatography was performed with SiO_2 (230-400 mesh) or by using an automated chromatography instrument with an appropriately sized column. Thin layer chromatography was performed on silica gel $60F_{254}$ plates (E. Merck). Non-UV active compounds were visualized on TLC using one of the following stains: $KMnO_4$, ninhydrin, *p*-anisaldehyde. ^1H and ^{13}C NMR spectra were recorded on an instrument operating at either 600MHz, and 151MHz respectively. LCMS data were collected using an HPLC instrument coupled to a low resolution mass spectrometer with single quadrupole ionization operating in either positive or negative ion mode. The analytical

method utilized a C_{18} column (2.1 × 50 mm, 1.8 μm) eluting with a linear gradient of 95%/5% water/CH_3CN (modified with 0.05% formic acid; T = 0 min flow = 0.35 mL/min) to 95%/5% CH_3CN/water (T = 3.5 min flow = 0.5 mL/min) then 95%/5% CH_3CN/water to T = 5min (0.5 mL/min). Peak detection was done at 254 nm and 230 nm for UV active compounds. High-resolution mass spectrometry (HRMS) spectra were obtained on a Thermo Scientific Q Exactive hybrid quadrupole-Orbitrap mass spectrometer equipped with a HESI source and using lock masses for correction. Samples were introduced into the HRMS via reversed phase HPLC on an Accucore Vanquish C18+ column (2.1 × 100 mm, 1.5 μm) eluting with a linear gradient of 95%/5% water/acetonitrile (modified with 0.1% formic acid) to 10%/90% water/acetonitrile over 8 min.

Experimental procedures and NMR data:

General procedure-1 (amidation); into a round bottom flask equipped with magnetic stir bar and septum under nitrogen, the acid compound (**1 equiv.**) was dissolved in DMF, amine (**1.1 equiv.**) was added followed by DIPEA (**1.5 equiv.**) and HATU (**1.1 equiv.**) at room temperature. The reaction was allowed to stir for 16h, after which time TLC and LCMS indicated complete consumption of starting material. The reaction was worked up by diluting with ethyl acetate and washed with Sat. aq $NaHCO_3$ and brine. The organic phase was collected and dried over anhydrous Na_2SO_4. Filtered and the solvent was removed under reduced pressure to give the crude product, which was used in next reaction without further purification.

General procedure-2 (-Boc- removal);[12] into a round bottom flask equipped with magnetic stir bar and septum, the Boc-compound (**1 equiv.**) was dissolved in dichloromethane and TFA (10% TFA in DCM, V/V, 10 mL/mmol) was added and allowed to stir at room temperature under nitrogen. After 2h the starting material was consumed according to TLC and LCMS. The volatiles were evaporated under reduced pressure and then redissolved in toluene and evaporated to remove excess TFA. The crude product as TFA salt was used in next reaction without further purification.

General procedure-3 (O-alkylation);[13] into a microwave vial equipped with magnetic stir bar and septum under nitrogen, the phenol compound (**1 equiv.**) was dissolved in acetone and 2-bromo-N,N-dimethylacetamide (**1.1 equiv.**) was added followed by K_2CO_3 (**1.5 equiv.**) and tetrabutylammonium iodide (TBAI; **0.1 equiv.**). The reaction vial was sealed and allowed to heat at 65 °C for 2h, after which time TLC and LCMS indicated complete consumption of starting material. The reaction was worked up by diluting with ethyl acetate and washed with water and brine. The organic phase was collected and dried over anhydrous Na_2SO_4. Filtered and the solvent was removed under reduced pressure to give the crude residue. Purification by silica gel chromatography (ethyl acetate/ hexanes) provided the pure product.

General procedure-4 (hydroxy ketone synthesis);[14] into a round bottom flask equipped with magnetic stir bar and septum, the methyl ketone (**1 equiv.**) was dissolved in methanol at 0 °C and added powdered potassium hydroxide (**6 equiv.**) followed by Iodobenzene diacetate (**1.1 equiv.**). The mixture was warmed to room temperature, stirred for 3 h, and then evaporated to dryness under reduced pressure. The reaction was worked up by diluting with ethyl acetate and washed with water. The organic phase was collected and dried over anhydrous Na_2SO_4. Filtered and thesolvent was removed under reduced pressure to give the crude residue. The residue was dissolved in a mixture of methanol and aqueous hydrochloric acid (2M) and stirred overnight at rt, after that time evaporated the reaction mixture to dryness under reduced pressure The reaction

was worked up by diluting with ethyl acetate and washed with water. The organic phase was collected and dried over anhydrous Na_2SO_4. Filtered and the solvent was removed under reduced pressure to give the crude residue. Purification by silica gel chromatography (ethyl acetate/ hexanes) provided the pure product.

General procedure-5 (Suzuki reaction);[15] the arylbromide (**1 equiv.**), boronic acid (**1.3 equiv.**), K_2CO_3 (**1.5 equiv.**) and Pd(dppf)Cl_2·DCM complex (**0.1 equiv.**) were placed in a vial equipped with a stir bar. The vial was sealed with a septum screw-cap, and then it was evacuated and filled with nitrogen (three cycles). 1, 4-dioxane and water (5:1 ratio) was added, and the resulting homogeneous reaction mixture was stirred vigorously at 110 °C for 1h. After which time TLC and LCMS indicated complete consumption of starting material. The reaction was worked up by diluting with ethyl acetate and washed with water and brine. The organic phase was collected and dried over anhydrous Na_2SO_4. Filtered and the solvent was removed under reduced pressure to give the crude residue. Purification by silica gel chromatography (ethyl acetate/ hexanes) provided the pure product.

General procedure-6 (aldehyde reduction); into a round bottom flask equipped with magnetic stir bar and septum under nitrogen, the aldehyde compound (**1 equiv.**) was dissolved in dry THF, solid $NaBH_4$ (**1.5 equiv.**) was added at 0 °C. After addition the reaction was allowed to stir at rt for 3 h, after which time TLC and LCMS indicated complete consumption of starting material. The reaction was worked up by diluting with EtOAc and washed with water and brine. The organic phase was collected and dried over anhydrous Na_2SO_4. Filtered and the solvent was removed under reduced pressure to give the crude residue. Purification by silica gel chromatography (ethyl acetate/ hexanes) provided the pure product.

General procedure-7 (reductive amination);[12] into a round bottom flask equipped with magnetic stir bar and septum under nitrogen, the aldehyde compound (**1 equiv.**) was dissolved in DCM, treated with methylamine (2M solution in THF, **1.5 equiv.**) followed by AcOH (**0.2 equiv.**) at rt. The solution was stirred at rt for 20 minutes and then treated with sodium triacetoxyborohydride (STAB, **1.5 equiv.**). The reaction was stirred at rt for 16 h. LCMS confirmed complete consumption of starting material. The reaction was worked up by diluting with DCM and washed with aq. sodium bicarbonate. The organic phase was collected and dried over anhydrous Na_2SO_4. Filtered and the solvent was removed under reduced pressure to give the crude residue. Purification by silica gel chromatography (ethyl acetate/ hexanes) provided the pure product.

General procedure-8 (Wittig);[16] into a round bottom flask equipped with magnetic stir bar and septum under nitrogen, the aldehyde compound (**1 equiv.**) was dissolved in dry DCM, (ethoxycarbonylmethylene)triphenylphosphorane (**1.1 equiv.**) was added at rt. The reaction was allowed to stir for 16 h, after which time TLC and LCMS indicated complete consumption of starting material. The reaction was worked up by diluting with DCM and washed with water and brine. The organic phase was collected and dried over anhydrous Na_2SO_4. Filtered and the solvent was removed under reduced pressure to give the crude residue. Purification by silica gel chromatography (ethyl acetate/ hexanes) provided the pure product.

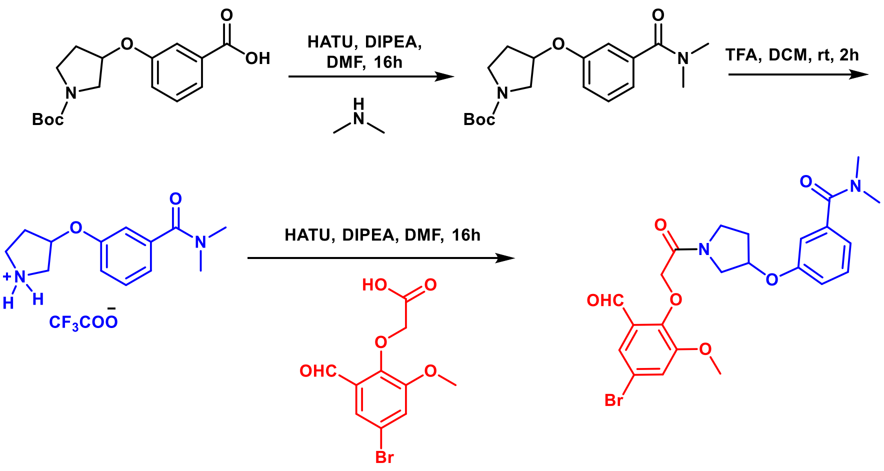

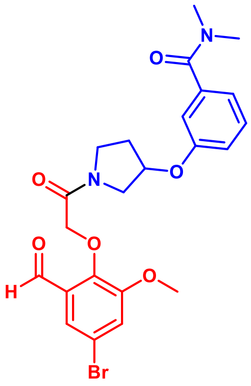

CDD-1712

Synthesized by following general procedure-1 & 2;

3-((1-(2-(4-bromo-2-formyl-6-methoxyphenoxy)acetyl)pyrrolidin-3-yl)oxy)-N,N-dimethylbenzamide: Molecular Formula: $C_{23}H_{25}BrN_2O_6$; **^{1}H NMR (600 MHz, CDCl$_3$)** δ 10.54 (s, 1H), 10.53 (s, 1H), 7.55 (t, *J* = 2.4 Hz, 2H), 7.34-7.29 (m, 2H), 7.21 (dd, *J* = 10.2, 2.4 Hz, 2H), 7.00 (dd, *J* = 17.9, 7.6 Hz, 2H), 6.93 – 6.88 (m, 2H), 5.05-5.04 (m, 1H), 4.97 – 4.96 (m, 1H), 4.86 (d, *J* = 4.2 Hz, 2H), 4.82-4.75 (m, 2H), 3.90 (s, 2H), 3.86 (s, 2H), 3.81 – 3.77 (m,1H), 3.74 – 3.60 (m, 4H), 3.17-3.03 (m, 1H), 3.11 (d, *J* = 3.9 Hz, 3H), 2.98-2.97 (m, 3H), 2.38-2.34 (3, 1H), 2.26-2.18 (m, 1H), 2.12-2.07 (m, 1H), 1.43 – 1.39 (m, 6H), 1.25 (s, 11H). **^{13}C NMR (151 MHz, CDCl$_3$)** δ 189.3, 189.2, 171.1, 171.0, 166.8, 166.7, 156.8, 152.9, 152.9, 149.4, 149.4, 137.9, 130.9, 129.9, 129.7, 122.0, 122.0, 120.7, 120.0, 119.8, 117.2, 117.1, 116.7, 114.0, 113.8, 74.3, 70.8, 70.5, 56.5, 56.5, 56.5, 55.5, 51.6, 51.0, 44.1, 43.5, 43.5, 39.6, 35.4, 31.9, 18.6, 17.2, 14.1, 12.5. HRMS (HESI-TOF) m/z calcd for $(M + H)^+$ 505.0974 and 507.0954, found 505.0968 and 507.0946.

Synthesized by following general procedure-3;

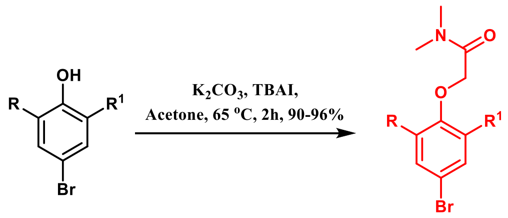

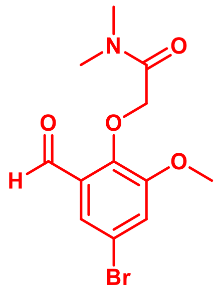

2-(4-bromo-2-formyl-6-methoxyphenoxy)-N,N-dimethylacetamide: Molecular Formula: $C_{12}H_{14}BrNO_4$; **^{1}H NMR (600 MHz, CDCl$_3$)** δ 10.52 (s, 1H), 7.55 (d, J = 2.2 Hz, 1H), 7.20 (d, J = 2.3 Hz, 1H), 4.89 (s, 2H), 3.89 (s, 3H), 3.00 (s, 3H), 2.95 (s, 3H). **^{13}C NMR (151 MHz, CDCl$_3$)** δ 189.3, 167.7, 152.9, 149.5, 131.0, 122.1, 120.7, 117.1, 70.3, 56.5, 36.0, 35.5.

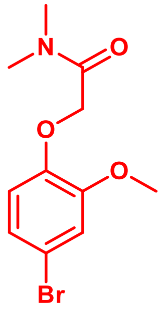

2-(4-bromo-2-methoxyphenoxy)-N,N-dimethylacetamide: Molecular Formula : $C_{11}H_{14}BrNO_3$: **^{1}H NMR (600 MHz, CDCl$_3$)** δ 6.99 – 6.98 (m, 2H), 6.81 – 6.80 (m, 1H), 4.72 (s, 2H), 3.85 (s, 3H), 3.08 (s, 3H), 2.96 (s, 3H). **^{13}C NMR (151 MHz, CDCl3)** δ 167.6, 150.4, 146.8, 123.5, 115.7, 115.4, 114.2, 68.7, 56.1, 36.5, 35.7.

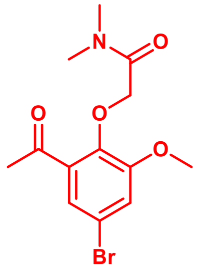

2-(2-acetyl-4-bromo-6-methoxyphenoxy)-N,N-dimethylacetamide: Molecular Formula: $C_{13}H_{16}BrNO_4$; **^{1}H NMR (600 MHz, CDCl$_3$)** δ 7.34 (dd, *J* = 2.3, 1.2 Hz, 1H), 7.13 (d, *J* = 2.6 Hz, 1H), 4.75 (s, 2H), 3.87 (s, 3H), 3.01 (s, 3H), 2.98 (s, 3H), 2.65 (s, 3H). **^{13}C NMR (151 MHz, CDCl3)** δ 198.9, 167.4, 153.3, 145.6, 135.3, 123.7, 118.8, 116.9, 70.9, 56.4, 36.2, 35.5, 31.4.

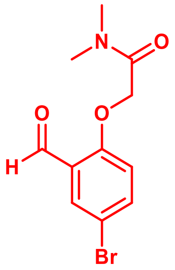

2-(4-bromo-2-formylphenoxy)-N,N-dimethylacetamide: Molecular Formula: $C_{11}H_{12}BrNO_3$; **^{1}H NMR (600 MHz, $CDCl_3$)** δ 10.45 (s, 1H), 7.92 (d, *J* = 2.6 Hz, 1H), 7.59-7.57 (m, 1H), 6.88 (d, *J* = 8.9 Hz, 1H), 4.84 (s, 2H), 3.08 (s, 3H), 2.98 (s, 3H). **^{13}C NMR (151 MHz, $CDCl_3$)** δ 188.0, 166.6, 159.3, 138.2, 131.2, 126.5, 115.1, 114.4, 67.4, 36.3, 35.7.

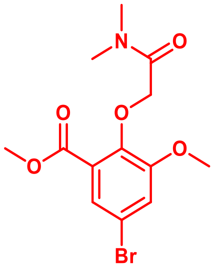

Methyl 5-bromo-2-(2-(dimethylamino)-2-oxoethoxy)-3-methoxybenzoate: Molecular Formula: $C_{13}H_{16}BrNO_5$; **^{1}H NMR (600 MHz, $CDCl_3$)** δ 7.45 (dd, *J* = 2.4, 1.0 Hz, 1H), 7.14 (d, *J* = 2.4 Hz, 1H), 4.67 (s, 2H), 3.87 (s, 3H), 3.85 (s, 3H), 3.08 (s, 3H), 2.98 (s, 3H). **^{13}C NMR (151 MHz, $CDCl_3$)** δ 167.6, 165.1, 154.0, 146.5, 127.6, 124.9, 119.1, 116.8, 72.0, 56.4, 52.5, 36.5, 35.5.

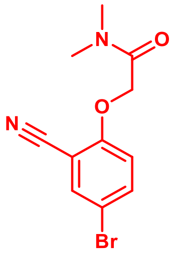

2-(4-bromo-2-cyanophenoxy)-N,N-dimethylacetamide: Molecular Formula: $C_{11}H_{11}BrN_2O_2$; **^{1}H NMR (600 MHz, $CDCl_3$)** δ 7.66 (dd, *J* = 2.6, 1.2 Hz, 1H), 7.59 (ddd, *J* = 9.1, 2.6, 1.2 Hz, 1H), 6.94 (dd, *J* = 9.0, 1.3 Hz, 1H), 4.84 (s, 2H), 3.11 (s, 2H), 2.96 (s, 1H). **^{13}C NMR (151 MHz, CDCl3)** δ 166.3, 158.8, 137.3, 135.9, 114.7, 114.7, 113.3, 104.0, 68.2, 36.7, 35.7.

Synthesized by following general procedure- 3 & 4;

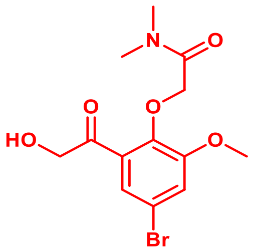

2-(4-bromo-2-(2-hydroxyacetyl)-6-methoxyphenoxy)-N,N-dimethylacetamide: Molecular Formula: $C_{13}H_{16}BrNO_5$; **^{1}H NMR (600 MHz, CDCl$_3$)** δ 7.55 (d, *J* = 2.3 Hz, 1H), 7.17 (d, *J* = 2.4 Hz, 1H), 4.89 (s, 2H), 4.81 (s, 2H), 3.88 (s, 3H), 2.98 (s, 3H), 2.96 (s, 3H). **^{13}C NMR (151 MHz, CDCl3)** δ 199.9, 167.5, 152.7, 146.6, 130.7, 124.2, 119.9, 116.7, 69.9, 69.6, 56.5, 36.0, 35.5.

Synthesized by following general procedure-5;

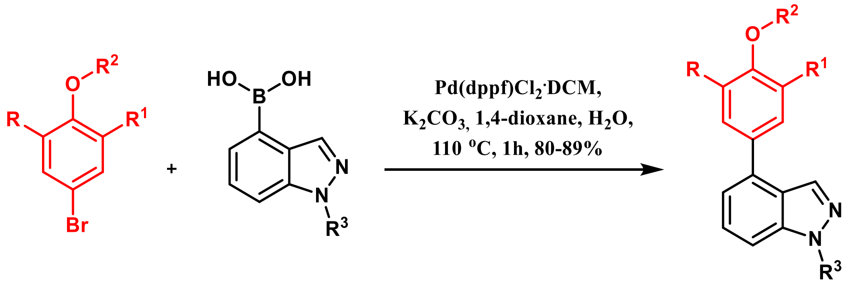

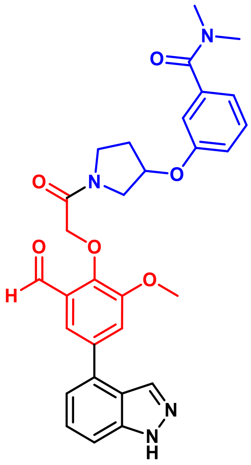

CDD-1714

3-((1-(2-(2-formyl-4-(1H-indazol-4-yl)-6-methoxyphenoxy)acetyl)pyrrolidin-3-yl)oxy)-N,N-dimethylbenzamide: Molecular Formula: $C_{30}H_{30}N_4O_6$; **^{1}H NMR (600 MHz, CDCl$_3$)** δ 10.70 (s, 1H), 10.67 (s, 1H), 8.18 (d, *J* = 3.6 Hz, 1H), 7.76 (dd, *J* = 6.2, 2.1 Hz, 1H), 7.49 (d, *J* = 8.4 Hz, 1H), 7.47 – 7.41 (m, 1H), 7.34-7.29 (m, 1H), 7.23 (dd, *J* = 7.0, 4.1 Hz, 1H), 7.03 – 6.99 (m, 1H), 6.96 – 6.89 (m, 2H), 5.06-5.04 (m, 1H), 4.97-4.96 (m, 2H), 4.88 (q, *J* = 14.4 Hz, 1H), 3.97 – 3.92 (m, 4H), 3.86 – 3.79 (m, 1H), 3.74 – 3.67 (m, 2H), 3.12-3.11 (m, 3H), 2.99-2.98 (m, 3H), 2.39 – 2.35 (m, 1H), 2.28 – 2.18 (m, 1H), 2.13-2.07 (m, 1H), 2.02 – 2.00 (m, 1H). **^{13}C NMR (151 MHz,**

$CDCl_3$) δ 190.5, 171.1, 167.1, 156.8, 150.0, 140.6, 137.9, 134.1, 133.8, 130.2, 129.7, 127.0, 120.4, 119.8, 119.1, 117.8, 117.2, 116.8, 114.0, 109.2, 71.0, 56.4, 51.8, 51.2, 44.2, 43.6, 39.6, 35.4, 31.9, 14.2. HRMS (HESI-TOF) m/z calcd for $(M + H)^+$ 543.2244, found 543.2241.

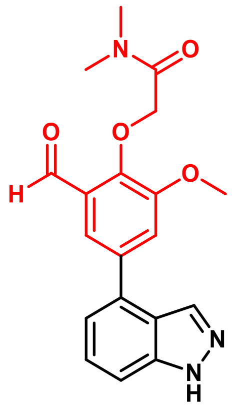

CDD-1713

2-(2-formyl-4-(1H-indazol-4-yl)-6-methoxyphenoxy)-N,N-dimethylacetamide: Molecular Formula: $C_{19}H_{19}N_3O_4$; **^{1}H NMR (600 MHz, $CDCl_3$)** δ 10.68 (s, 1H), 8.20 (s, 1H), 7.76 (d, *J* = 2.1 Hz, 1H), 7.51 (d, *J* = 8.4 Hz, 1H), 7.49 – 7.41 (m, 2H), 7.24 (d, *J* = 7.0 Hz, 1H), 5.00 (s, 2H), 3.97 (s, 3H), 3.08 (s, 3H), 3.01 (s, 3H). **^{13}C NMR (151 MHz, $CDCl_3$)** δ 190.5, 168.0, 152.4, 150.1, 136.1, 130.3, 127.1, 120.5, 119.2, 117.9, 109.2, 70.9, 56.4, 36.2, 35.6, 29.7. HRMS (HESI-TOF) m/z calcd for $(M + H)^+$ 354.1454, found 354.1443.

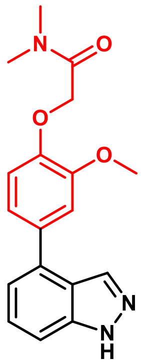

CDD-1793

2-(4-(1H-indazol-4-yl)-2-methoxyphenoxy)-N,N-dimethylacetamide: Molecular Formula : $C_{18}H_{19}N_3O_3$: **1H NMR (600 MHz, $CDCl_3$)** δ 8.23 (s, 1H), 7.51 – 7.40 (m, 2H), 7.25 – 7.18 (m, 3H), 7.07 (d, *J* = 8.1 Hz, 1H), 4.85 (s, 2H), 3.94 (s, 3H), 3.16 (s, 3H), 3.02 (s, 3H). **^{13}C NMR (151 MHz, $CDCl_3$)** δ 168.0, 149.7, 147.3, 134.0, 127.0, 120.9, 120.0, 114.4, 112.3, 108.6, 68.6, 56.1, 36.7, 35.9. HRMS (HESI-TOF) m/z calcd for $(M + H)^+$ 326.1505, found 326.1487.

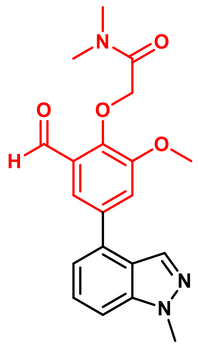

CDD-1847

2-(2-formyl-6-methoxy-4-(1-methyl-1H-indazol-4-yl)phenoxy)-N,N-dimethylacetamide: Molecular Formula : $C_{20}H_{21}N_3O_4$; **^{1}H NMR (600 MHz, CDCl$_3$)** δ 10.67 (s, 1H), 8.09 (d, *J* = 0.9 Hz, 1H), 7.78 (d, *J* = 2.1 Hz, 1H), 7.48 – 7.43 (m, 2H), 7.40 (d, *J* = 8.4 Hz, 1H), 7.23 (d, *J* = 7.0 Hz, 1H), 4.99 (s, 2H), 4.13 (s, 3H), 3.97 (s, 3H), 3.07 (s, 3H), 3.00 (s, 3H). **^{13}C NMR (151 MHz, CDCl$_3$)** δ 190.5, 168.0, 152.4, 150.0, 140.4, 136.1, 134.0, 132.0, 130.3, 126.5, 122.3, 120.0, 119.2, 117.9, 108.4, 70.8, 56.4, 36.2, 35.8, 35.6. HRMS (HESI-TOF) m/z calcd for $(M + H)^+$ 368.1610, found 368.1604.

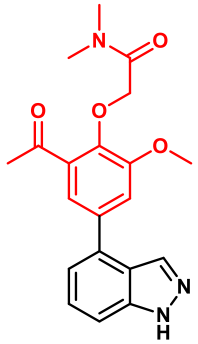

CDD-1883

2-(2-acetyl-4-(1H-indazol-4-yl)-6-methoxyphenoxy)-N,N-dimethylacetamide: Molecular Formula: $C_{20}H_{21}N_3O_4$; **^{1}H NMR (600 MHz, CDCl$_3$)** δ 8.20 (s, 1H), 7.54 (d, *J* = 2.1 Hz, 1H), 7.51 (d, *J* = 8.4 Hz, 1H), 7.45 (dd, *J* = 8.4, 7.0 Hz, 1H), 7.37 (d, *J* = 2.1 Hz, 1H), 7.24 (d, *J* = 7.0 Hz, 1H), 4.87 (s, 2H), 3.95 (s, 3H), 3.09 (s, 3H), 3.03 (s, 3H), 2.74 (s, 3H). **^{13}C NMR (151 MHz, CDCl$_3$)** δ 200.3, 167.8, 152.8, 146.1, 136.1, 134.6, 120.9, 120.4, 115.7, 71.3, 56.4, 36.4, 35.6, 31.6. HRMS (HESI-TOF) m/z calcd for $(M + H)^+$ 368.1610, found 368.1606.

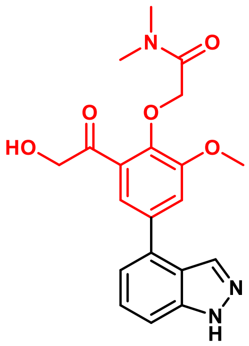

CDD-1886

2-(2-(2-hydroxyacetyl)-4-(1H-indazol-4-yl)-6-methoxyphenoxy)-N,N-dimethylacetamide: Molecular Formula: $C_{20}H_{21}N_3O_5$; **^{1}H NMR (600 MHz, CDCl$_3$)** δ 8.20 (s, 1H), 7.77 (d, *J* = 2.1 Hz, 1H), 7.51 (d, *J* = 8.3 Hz, 1H), 7.47 (dd, *J* = 8.4, 7.0 Hz, 1H), 7.42 (d, *J* = 2.1 Hz, 1H), 7.24 (s, 1H), 4.99 (s, 2H), 4.91 (s, 2H), 3.96 (s, 3H), 3.05 (s, 3H), 3.00 (s, 3H). **^{13}C NMR (151 MHz, CDCl$_3$)** δ 200.8, 167.7, 134.2, 129.9, 127.1, 121.6, 121.4, 120.5, 116.9, 109.1, 70.3, 69.7, 56.4, 36.1, 35.6. HRMS (HESI-TOF) m/z calcd for $(M + H)^+$ 384.1559, found 384.1553.

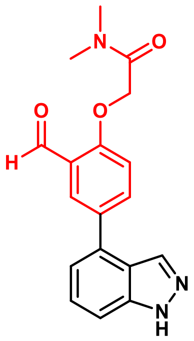

CDD-1976

2-(2-formyl-4-(1H-indazol-4-yl)phenoxy)-N,N-dimethylacetamide: Molecular Formula: $C_{18}H_{17}N_3O_3$; **^{1}H NMR (600 MHz, MeOD)** δ 10.58 (s, 1H), 8.11 (s, 2H), 7.94 (d, *J* = 8.8 Hz, 1H), 7.53 – 7.43 (m, 2H), 7.24-7.20 (m, 2H), 5.10 (s, 2H), 3.13 (s, 3H), 3.00 (s, 3H). **^{13}C NMR (151 MHz, CDCl$_3$)** δ 193.7, 172.2, 164.3, 144.9, 139.3, 137.0, 136.4, 131.1, 130.7, 125.0, 123.5, 117.7, 113.0, 69.9, 38.9, 38.5. HRMS (HESI-TOF) m/z calcd for $(M + H)^+$ 324.1348, found 324.1346.

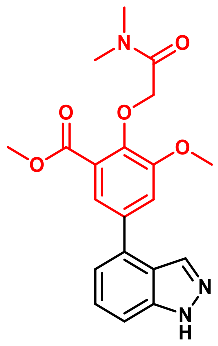

CDD-1982

Methyl 2-(2-(dimethylamino)-2-oxoethoxy)-5-(1H-indazol-4-yl)-3-methoxybenzoate: Molecular Formula: $C_{20}H_{21}N_3O_5$; **^{1}H NMR (600 MHz, CDCl$_3$)** δ 8.23 (s, 1H), 7.66 (s, 1H), 7.53 (d, *J* = 8.3 Hz, 1H), 7.44 (t, *J* = 7.7 Hz, 1H), 7.36 (d, *J* = 2.2 Hz, 1H), 7.22 (d, *J* = 7.0 Hz, 1H), 4.82 (s, 2H), 3.91 (d, *J* = 8.0 Hz, 6H), 3.17 (s, 3H), 3.05 (s, 3H). **^{13}C NMR (151 MHz, CDCl$_3$)** δ 168.1, 166.3, 153.5, 146.8, 136.3, 133.9, 127.0, 126.7, 122.2, 120.4, 115.9, 109.5, 72.3, 56.4, 52.4, 36.7, 35.7. HRMS (HESI-TOF) m/z calcd for $(M + H)^+$ 384.1559, found 384.1559.

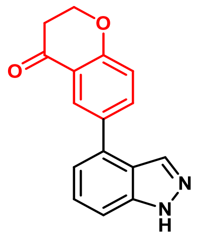

CDD-2037

6-(1H-indazol-4-yl)chroman-4-one: Molecular Formula: $C_{16}H_{12}N_2O_2$; **^{1}H NMR (600 MHz, CDCl$_3$)** δ 8.27 (s, 1H), 8.24 (d, *J* = 2.4 Hz, 1H), 7.84-7.83 (m, 1H), 7.58 (d, *J* = 8.5 Hz, 1H), 7.53 (t, *J* = 7.8 Hz, 1H), 7.28 (d, *J* = 7.2 Hz, 1H), 7.16 (d, *J* = 8.5 Hz, 1H), 4.63 (t, *J* = 6.5 Hz, 2H), 2.90 (t, *J* = 6.5 Hz, 2H). **^{13}C NMR (151 MHz, CDCl$_3$)** δ 191.6, 161.6, 135.9, 133.0, 127.7, 126.7, 121.6, 120.7, 118.6, 67.2, 37.8, 29.7. HRMS (HESI-TOF) m/z calcd for $(M + H)^+$ 265.0977, found 265.0967.

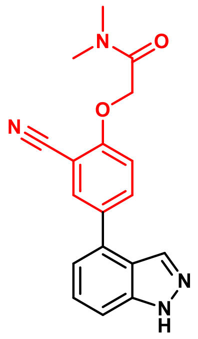

CDD-2038

2-(2-cyano-4-(1H-indazol-4-yl)phenoxy)-N,N-dimethylacetamide: Molecular Formula: $C_{18}H_{16}N_4O_2$; **^{1}H NMR (600 MHz, $CDCl_3$)** δ 8.13 (s, 1H), 7.84 (d, *J* = 2.1 Hz, 1H), 7.79 (dd, *J* = 8.7, 2.4 Hz, 1H), 7.54 (d, *J* = 8.4 Hz, 1H), 7.45 (t, *J* = 7.7 Hz, 1H), 7.18 (d, *J* = 8.7 Hz, 1H), 7.14 (d, *J* = 7.1 Hz, 1H), 4.95 (s, 2H), 3.20 (s, 3H), 3.03 (s, 3H). **^{13}C NMR (151 MHz, $CDCl_3$)** δ 166.8, 159.1, 140.7, 134.3, 133.7, 133.6, 133.3, 132.4, 127.3, 121.4, 120.4, 116.0, 113.4, 109.6, 102.7, 68.3, 36.9, 35.9. HRMS (HESI-TOF) m/z calcd for $(M + H)^+$ 321.1352, found 321.1338.

Synthesized by following general procedure-6;

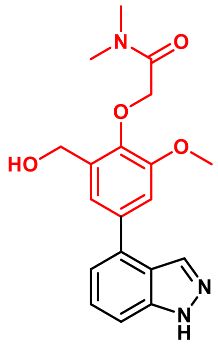

CDD-1776

2-(2-(hydroxymethyl)-4-(1H-indazol-4-yl)-6-methoxyphenoxy)-N,N-dimethylacetamide; Molecular Formula: $C_{19}H_{21}N_3O_4$; **1H NMR (600 MHz, $CDCl_3$)** δ 8.21 (s, 1H), 7.48 – 7.42 (m, 2H), 7.22 – 7.20(m, 3H), 5.02 (s, 2H), 4.78 (s, 2H), 3.94 (s, 3H), 3.02 (d, *J* = 2.7 Hz, 6H). **^{13}C NMR (151 MHz, $CDCl_3$)** δ 169.9, 151.4, 146.3, 140.6, 135.5, 135.4, 134.9, 134.5, 127.1, 122.4, 121.8, 120.2, 112.7, 108.6, 69.4, 61.9, 56.2, 35.9, 35.8. HRMS (HESI-TOF) m/z calcd for $(M + H)^+$ 356.1610, found 356.1602.

Synthesized by following general procedure-7;

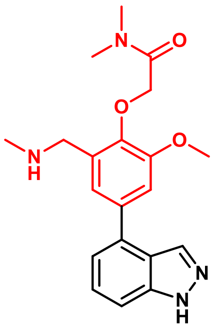

CDD-1777

2-(4-(1H-indazol-4-yl)-2-methoxy-6-((methylamino)methyl)phenoxy)-N,N-dimethylacetamide: Molecular Formula : $C_{20}H_{24}N_4O_3$; **^{1}H NMR (800 MHz, CDCl$_3$)** δ 8.22 (s, 1H), 7.55 (d, *J* = 8.3 Hz, 1H), 7.44 (dd, *J* = 8.3, 7.0 Hz, 1H), 7.31 (d, *J* = 5.4 Hz, 2H), 7.21 (d, *J* = 7.1 Hz, 1H), 5.24 (s, 2H), 4.29 (s, 2H), 3.96 (s, 3H), 3.03 (s, 3H), 3.01 (s, 3H), 2.78 (s, 3H). **^{13}C NMR (201 MHz, CDCl$_3$)** δ 170.4, 150.9, 145.4, 135.8, 133.6, 127.0, 124.4, 124.3, 120.3, 114.6, 109.4, 68.6, 56.2, 49.0, 36.0, 35.8, 32.3. HRMS (HESI-TOF) m/z calcd for $(M + H)^+$ 369.1927, found 369.1912.

Synthesized by following general procedure-8;

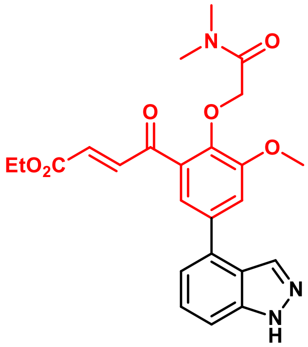

CDD-1971

ethyl (E)-4-(2-(2-(dimethylamino)-2-oxoethoxy)-5-(1H-indazol-4-yl)-3-methoxyphenyl)-4-oxobut-2-enoate: Molecular Formula: $C_{24}H_{25}N_3O_6$; **^{1}H NMR (600 MHz, CDCl$_3$)** δ 8.13 (d, *J* = 16.1 Hz, 1H), 7.51 – 7.43 (m, 4H), 7.24 (s, 3H), 6.53 (d, *J* = 16.1 Hz, 1H), 4.81 (s, 2H), 4.27 (q, *J* = 7.1 Hz, 2H), 3.94-3.93 (s, 3H), 3.22 (s, 3H), 3.03 (s, 3H), 1.34 (t, *J* = 7.1 Hz, 3H). **^{13}C NMR (151 MHz, CDCl$_3$)** δ 167.0, 152.6, 139.2, 129.0, 120.2, 114.2, 72.0, 60.5, 56.2, 36.8, 35.6, 29.7, 14.4. HRMS (HESI-TOF) m/z calcd for $(M + H)^+$ 452.1822, found 424.1853.

References:

1 Du, H. C., Matzuk, M. M. & Chen, Y. C. Synthesis of 5-substituted tetrazoles via DNA-conjugated nitrile. *Organic & biomolecular chemistry* **18**, 9221-9226, doi:10.1039/d0ob02021d (2020).

2 Faver, J. C. *et al.* Quantitative Comparison of Enrichment from DNA-Encoded Chemical Library Selections. *ACS combinatorial science* **21**, 75-82, doi:10.1021/acscombsci.8b00116 (2019).

3 Chen, Y. C. *et al.* C-N Coupling of DNA-Conjugated (Hetero)aryl Bromides and Chlorides for DNA-Encoded Chemical Library Synthesis. *Bioconjugate chemistry* **31**, 770-780, doi:10.1021/acs.bioconjchem.9b00863 (2020).

4 Li, J. Y. & Huang, H. Development of DNA-Compatible Suzuki-Miyaura Reaction in Aqueous Media. *Bioconjugate chemistry* **29**, 3841-3846, doi:10.1021/acs.bioconjchem.8b00676 (2018).

5 Du, H. C. & Huang, H. DNA-Compatible Nitro Reduction and Synthesis of Benzimidazoles. *Bioconjugate chemistry* **28**, 2575-2580, doi:10.1021/acs.bioconjchem.7b00416 (2017).

6 Du, H. C. *et al.* A Mild, DNA-Compatible Nitro Reduction Using B(2)(OH)(4). *Organic letters* **21**, 2194-2199, doi:10.1021/acs.orglett.9b00497 (2019).

7 Li, J. Y. *et al.* Palladium-Catalyzed Hydroxycarbonylation of (Hetero)aryl Halides for DNA-Encoded Chemical Library Synthesis. *Bioconjugate chemistry* **30**, 2209-2215, doi:10.1021/acs.bioconjchem.9b00447 (2019).

8 Du, H. C., Bangs, M. C., Simmons, N. & Matzuk, M. M. Multistep Synthesis of 1,2,4-Oxadiazoles via DNA-Conjugated Aryl Nitrile Substrates. *Bioconjugate chemistry* **30**, 1304-1308, doi:10.1021/acs.bioconjchem.9b00188 (2019).

9 Clark, M. A. *et al.* Design, synthesis and selection of DNA-encoded small-molecule libraries. *Nature chemical biology* **5**, 647-654, doi:10.1038/nchembio.211 (2009).

10 Satz, A. L. *et al.* DNA Compatible Multistep Synthesis and Applications to DNA Encoded Libraries. *Bioconjugate chemistry* **26**, 1623-1632, doi:10.1021/acs.bioconjchem.5b00239 (2015).

11 Grygorenko, O. O. *et al.* Focused enumeration and assessing the structural diversity of scaffold libraries: conformationally restricted bicyclic secondary diamines. *Molecular diversity* **16**, 477-487, doi:10.1007/s11030-012-9381-2 (2012).

12 Chamakuri, S., Shah, M. M., Yang, D. C. H., Santini, C. & Young, D. W. Practical and scalable synthesis of orthogonally protected-2-substituted chiral piperazines. *Organic & biomolecular chemistry* **18**, 8844-8849, doi:10.1039/d0ob01713b (2020).

13 Chamakuri, S., Jogula, S. & Arya, P. Regio- and Stereocontrolled Dieckmann Approach to Treprostinil-Inspired, Polycyclic Scaffold For Building Macrocyclic Diversity. *ACS combinatorial science* **17**, 437-441, doi:10.1021/acscombsci.5b00076 (2015).

14 Baś, S., Woźniak, Ł., Cygan, J. & Mlynarski, J. Asymmetric syn-Aldol Reaction of α-Hydroxy Ketones with Tertiary Amine Catalysts. *European Journal of Organic Chemistry* **2013**, 6917-6923 (2013).

15 Migliorini, A., Oliviero, C., Gasperi, T. & Loreto, M. A. The Suzuki reaction applied to the synthesis of novel pyrrolyl and thiophenyl indazoles. *Molecules (Basel, Switzerland)* **17**, 4508-4521, doi:10.3390/molecules17044508 (2012).

16 Reddy Guduru, S. K. *et al.* Synthesis of Enantiomerically Pure 3-Substituted Piperazine-2-acetic Acid Esters as Intermediates for Library Production. *The Journal of organic chemistry* **83**, 11777-11793, doi:10.1021/acs.joc.8b01708 (2018).

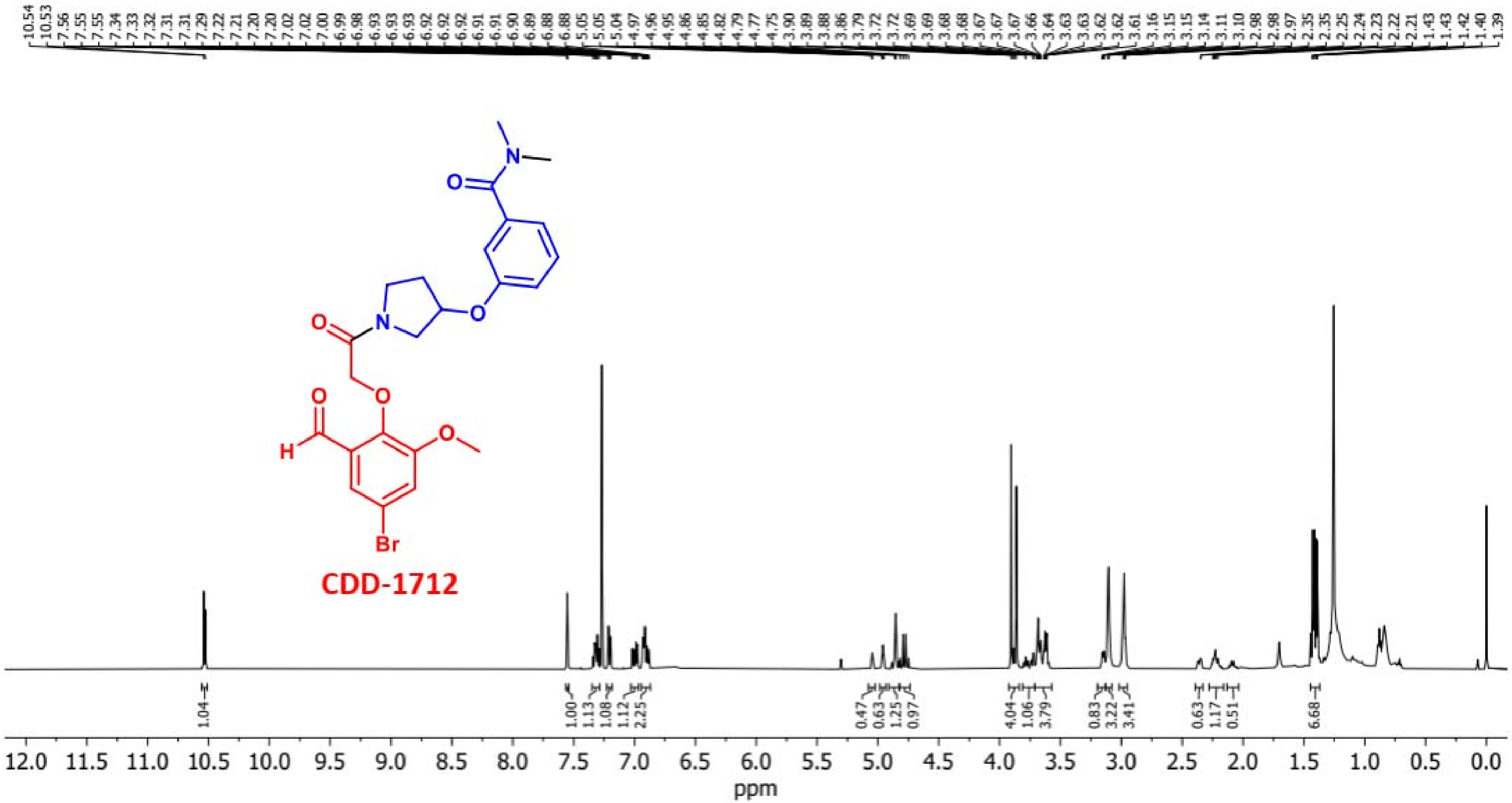
CDD-1712
ppm
12.0 11.5 11.0 10.5 10.0 9.5 9.0 8.5 8.0 7.5 7.0 6.5 6.0 5.5 5.0 4.5 4.0 3.5 3.0 2.5 2.0 1.5 1.0 0.5 0.0

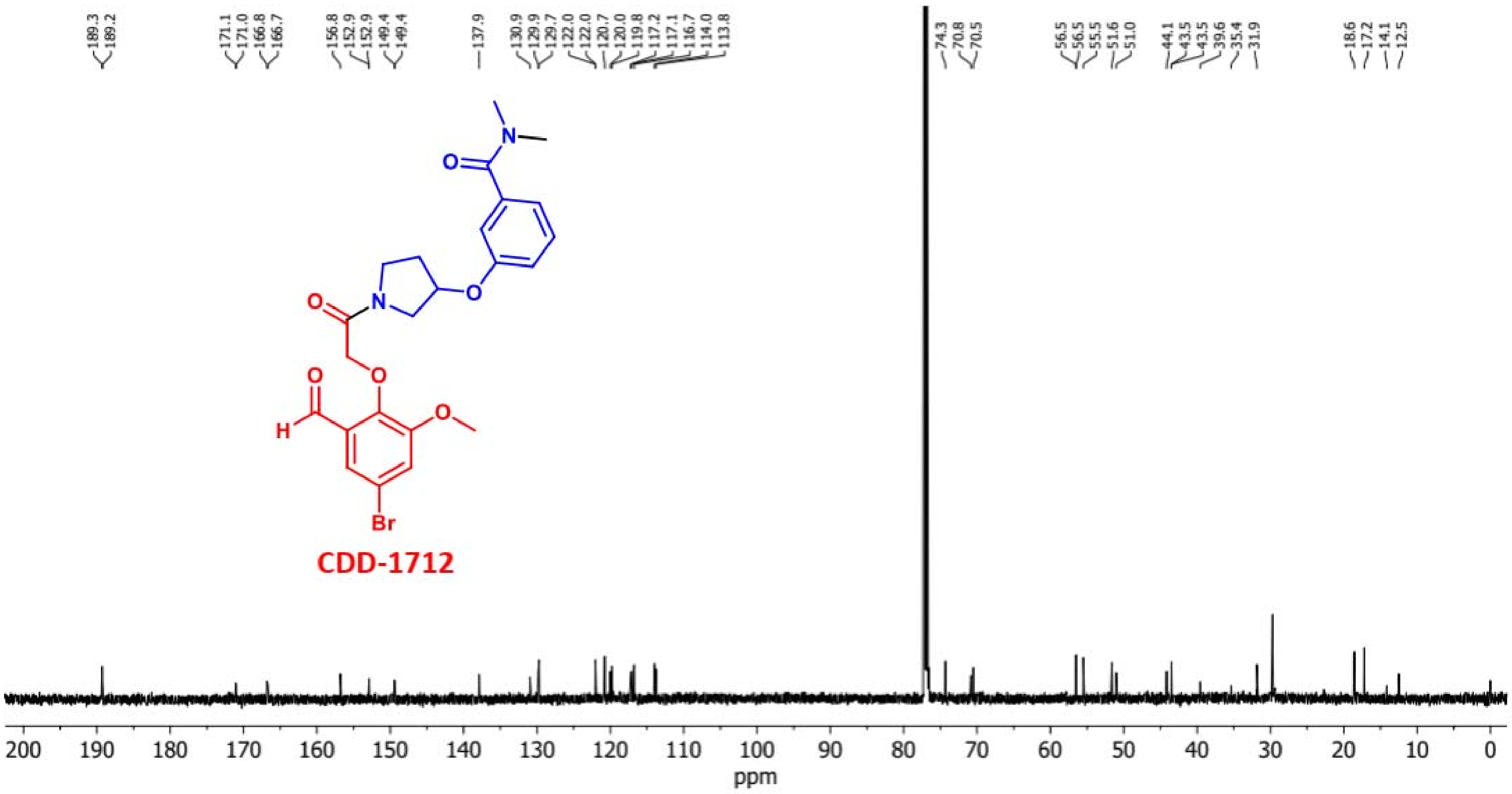

189.3
189.2
171.1
171.0
166.8
166.7
156.8
152.9
152.9
149.4
149.4
137.9
130.9
129.9
129.7
122.0
122.0
120.7
120.0
119.8
117.2
117.1
116.7
114.0
113.8
74.3
70.8
70.5
56.5
56.5
55.5
51.6
51.0
44.1
43.5
43.5
39.6
35.4
31.9
18.6
17.2
14.1
12.5
CDD-1712
200 190 180 170 160 150 140 130 120 110 100 90 80 70 60 50 40 30 20 10 0
ppm

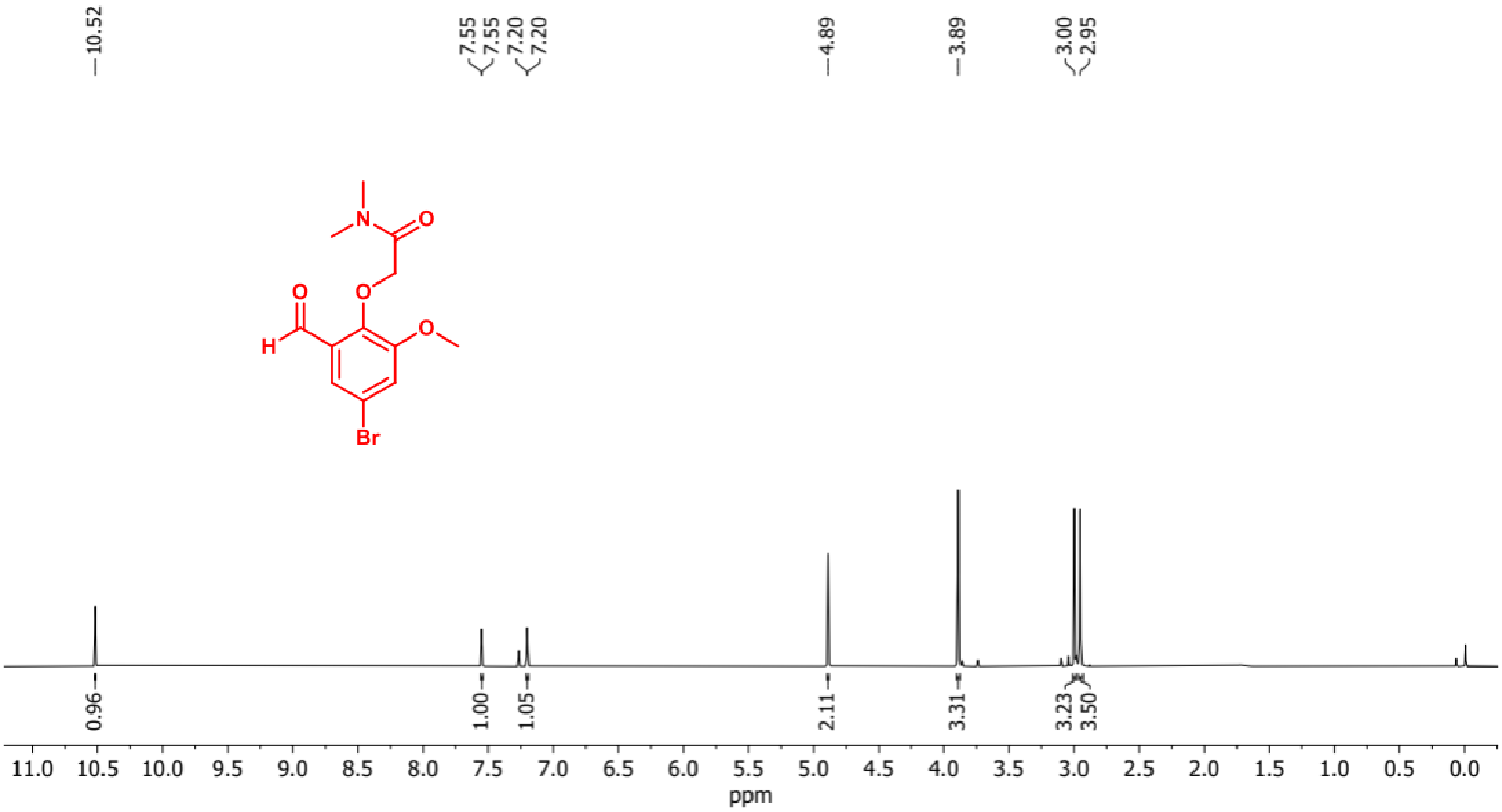

10.52
7.55
7.55
7.20
7.20
4.89
3.89
3.00
2.95
O
N
O
O
H
O
Br
0.96
1.00
1.05
2.11
3.31
3.23
3.50
11.0
10.5
10.0
9.5
9.0
8.5
8.0
7.5
7.0
6.5
6.0
5.5
5.0
4.5
4.0
3.5
3.0
2.5
2.0
1.5
1.0
0.5
0.0
ppm

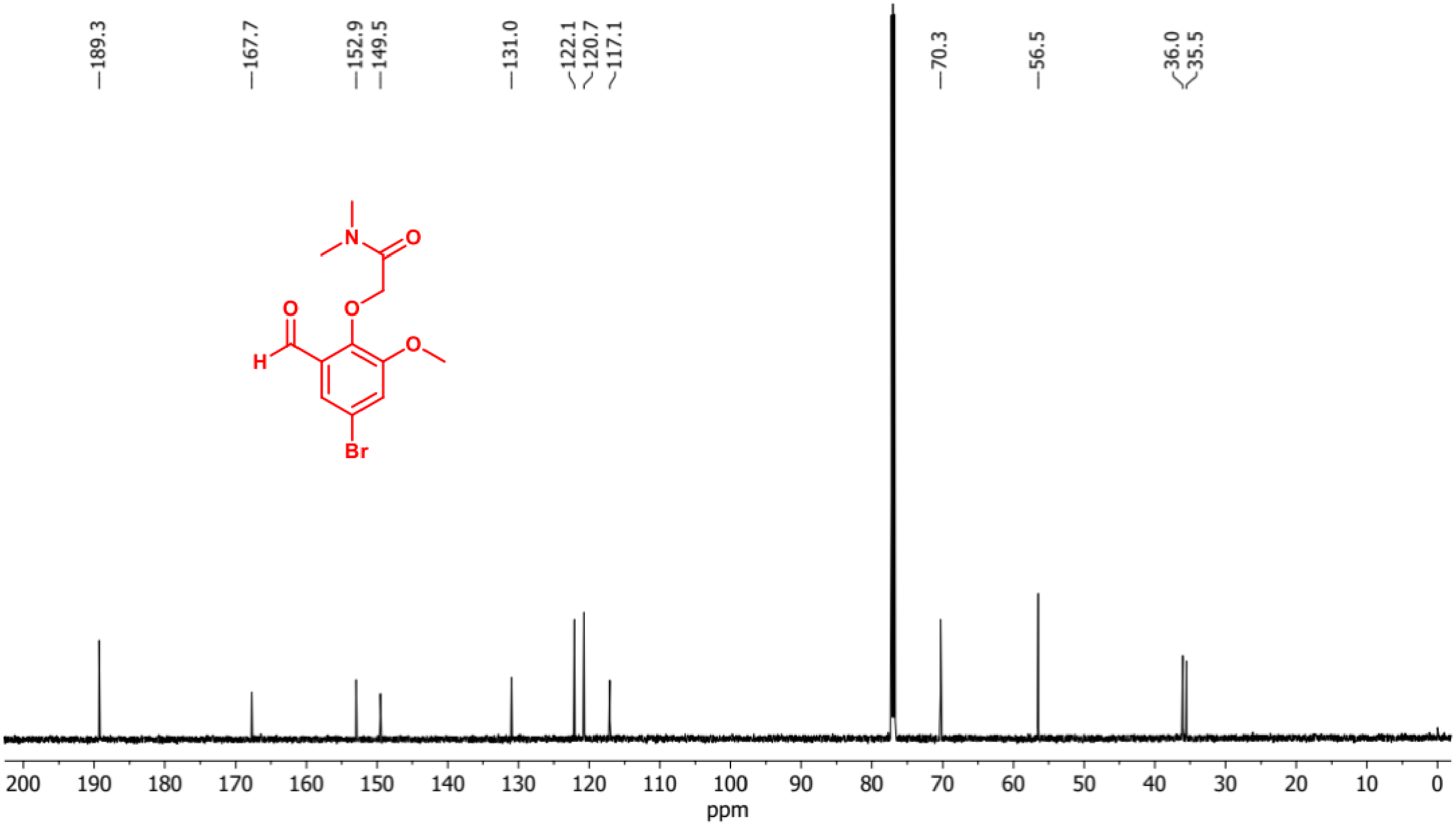

189.3
167.7
152.9
149.5
131.0
122.1
120.7
117.1
70.3
56.5
36.0
35.5
N
O
O
O
H
O
Br
200 190 180 170 160 150 140 130 120 110 100 90 80 70 60 50 40 30 20 10 0
ppm

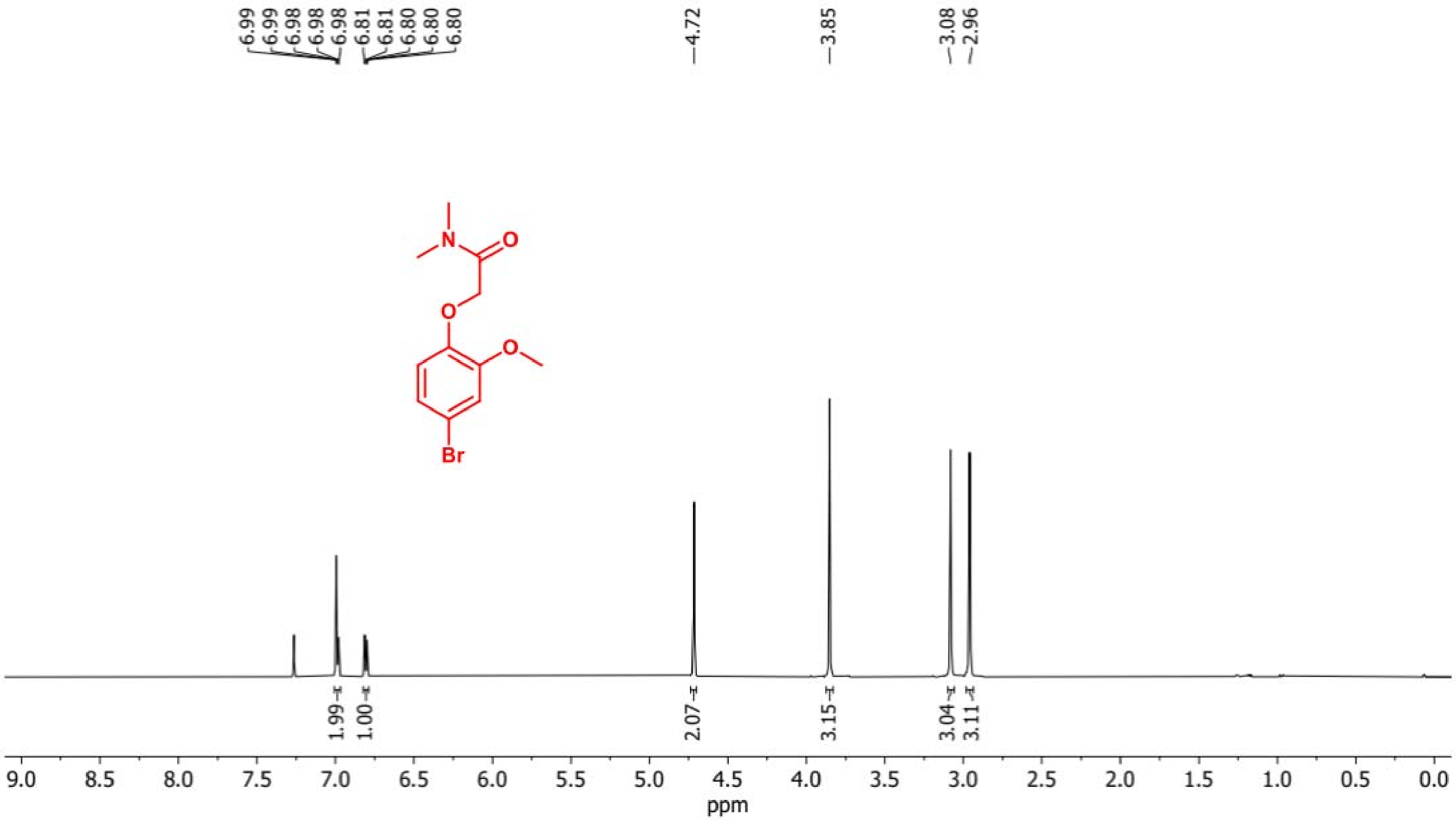
6.99
6.99
6.98
6.98
6.98
6.81
6.81
6.80
6.80
6.80
4.72
3.85
3.08
2.96
O
N
O
O
Br
1.99
1.00
2.07
3.15
3.04
3.11
9.0
8.5
8.0
7.5
7.0
6.5
6.0
5.5
5.0
4.5
4.0
3.5
3.0
2.5
2.0
1.5
1.0
0.5
0.0
ppm

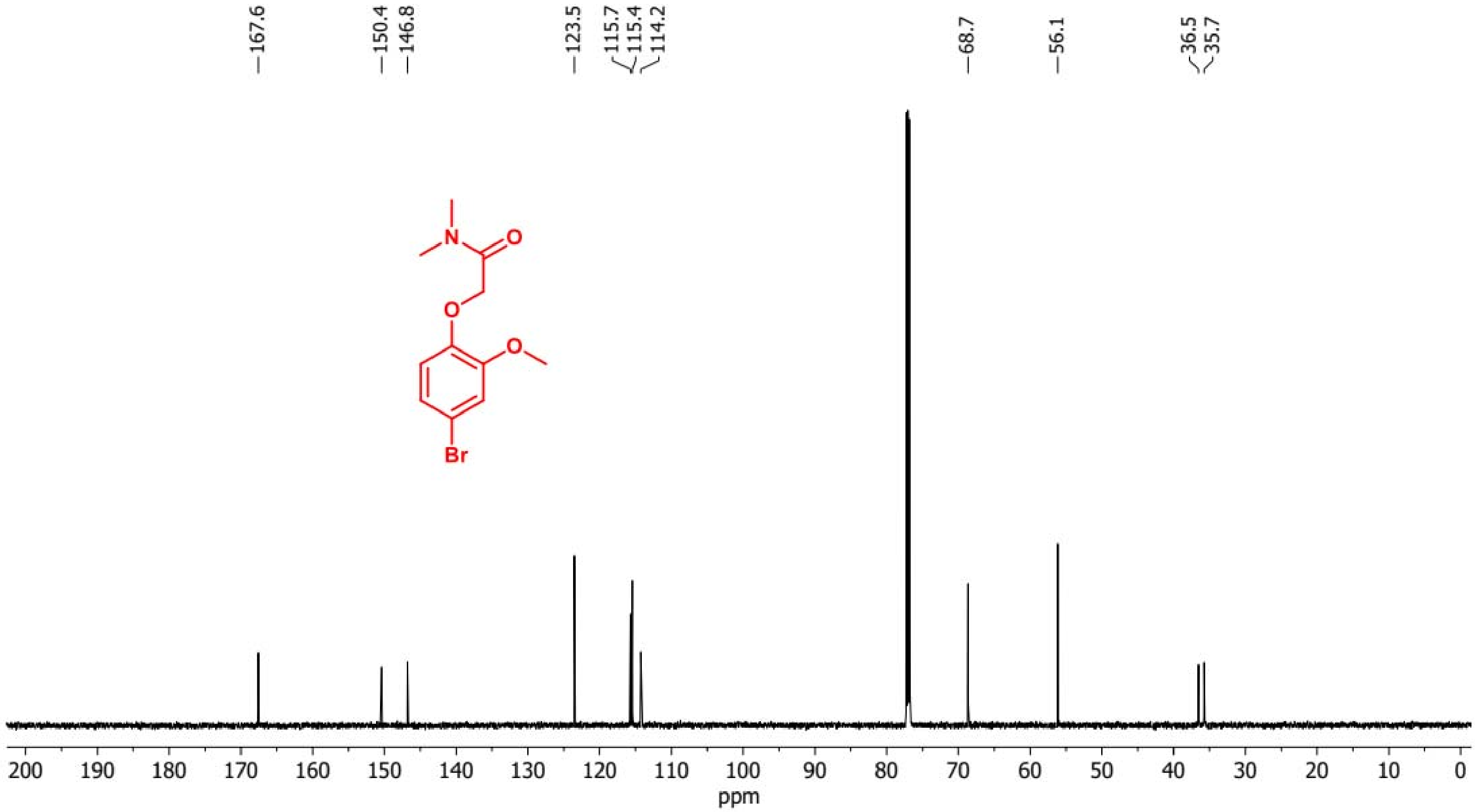

167.6
150.4
146.8
123.5
115.7
115.4
114.2
68.7
56.1
36.5
35.7
N
O
O
O
Br
200
190
180
170
160
150
140
130
120
110
100
90
80
70
60
50
40
30
20
10
0
ppm

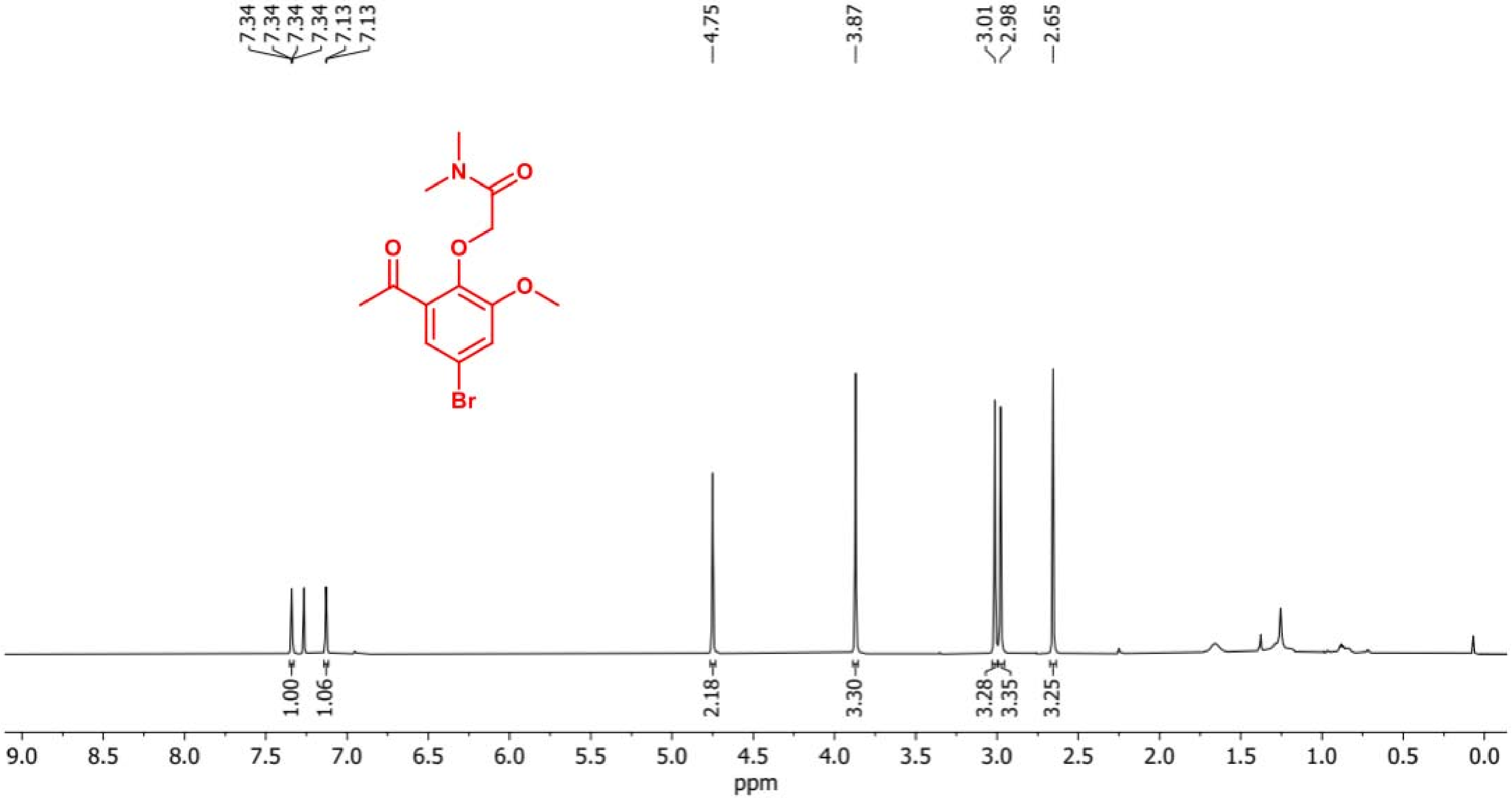
7.34
7.34
7.34
7.34
7.13
7.13
4.75
3.87
3.01
2.98
2.65
N
O
O
O
O
Br
1.00
1.06
2.18
3.30
3.28
3.35
3.25
9.0
8.5
8.0
7.5
7.0
6.5
6.0
5.5
5.0
4.5
4.0
3.5
3.0
2.5
2.0
1.5
1.0
0.5
0.0
ppm

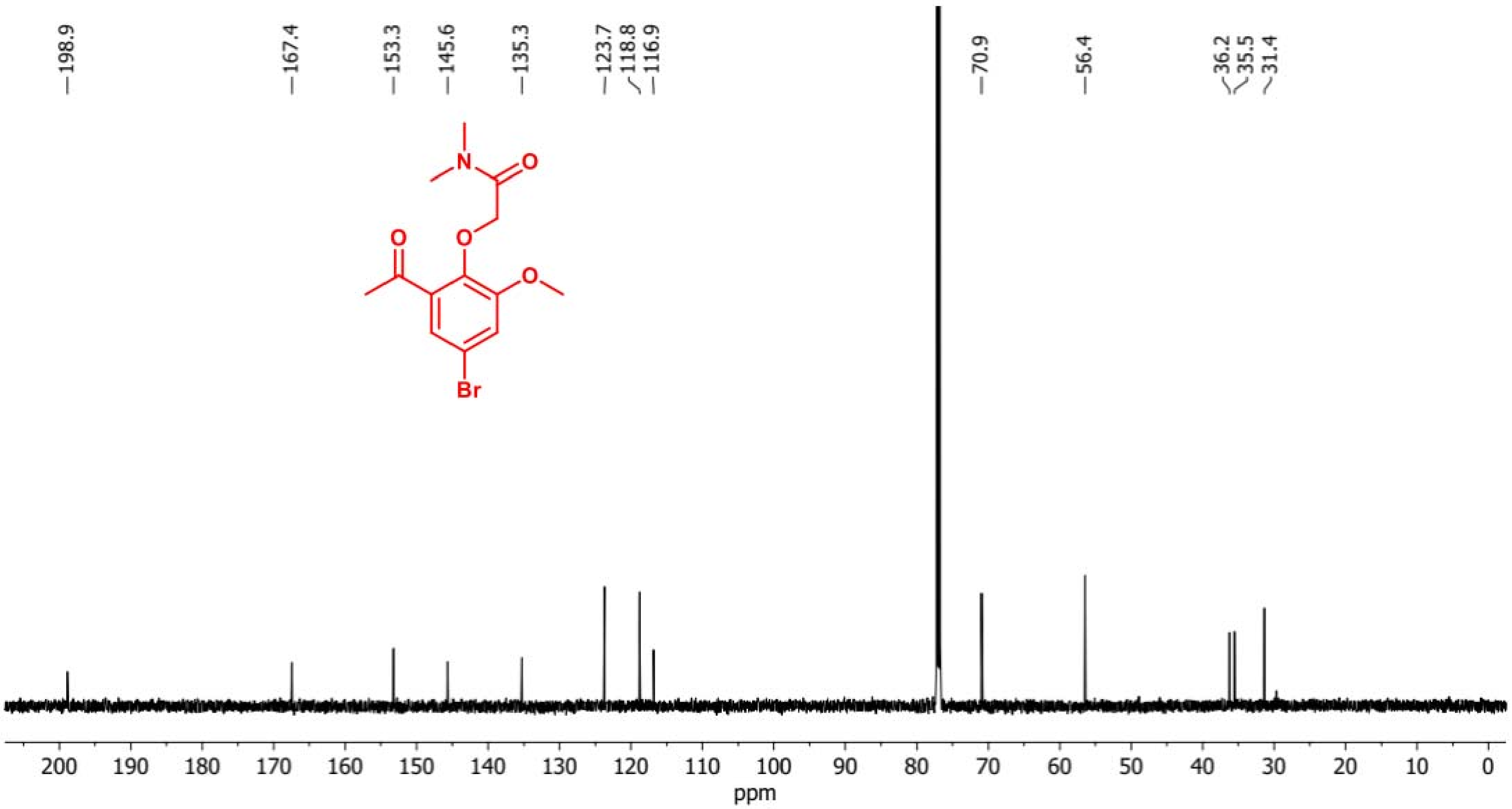
198.9
167.4
153.3
145.6
135.3
123.7
118.8
116.9
70.9
56.4
36.2
35.5
31.4
N
O
O
O
O
O
Br
200
190
180
170
160
150
140
130
120
110
100
90
80
70
60
50
40
30
20
10
0
ppm

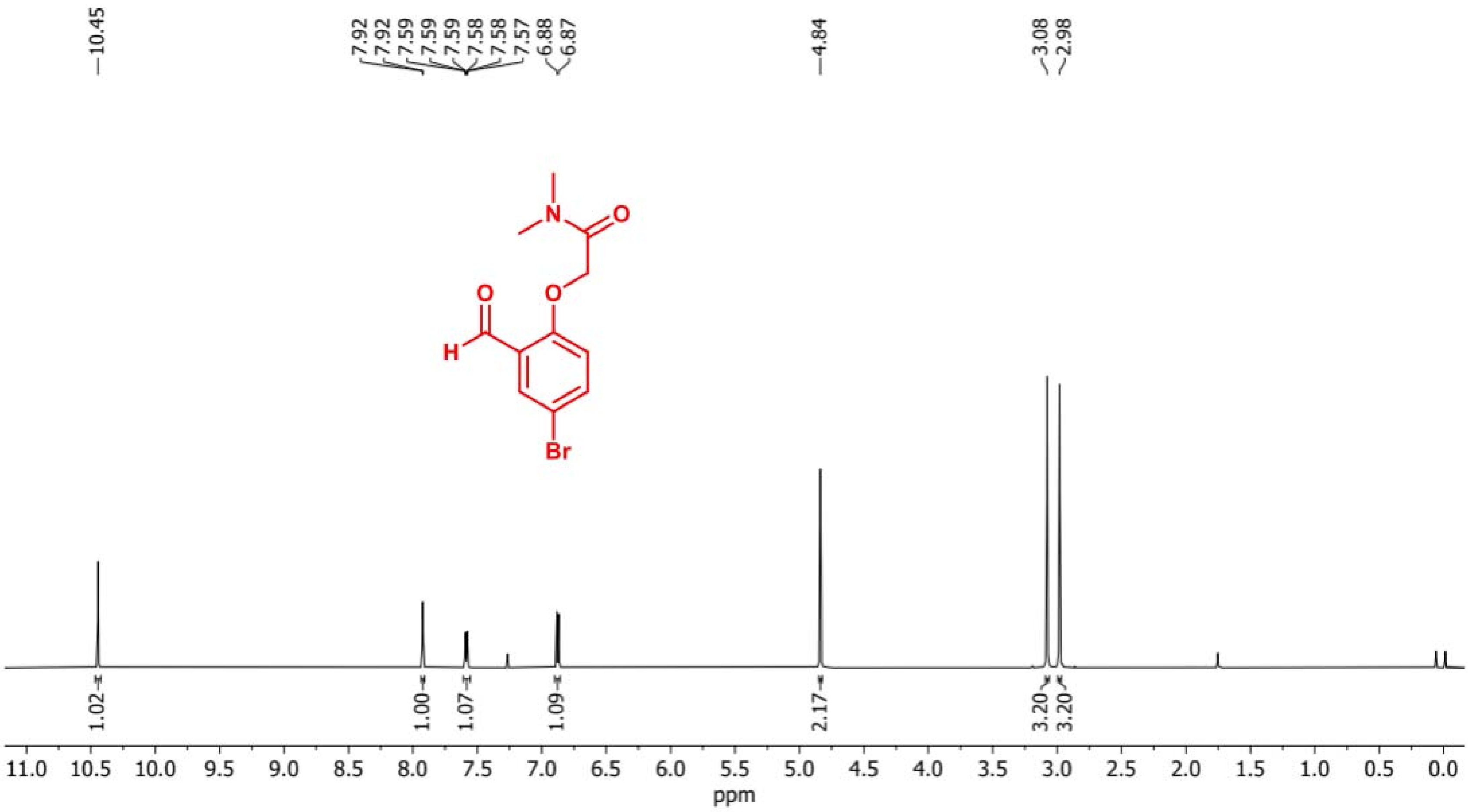
10.45
7.92
7.92
7.59
7.59
7.59
7.58
7.58
7.57
6.88
6.87
4.84
3.08
2.98
N
O
O
O
H
Br
1.02
1.00
1.07
1.09
2.17
3.20
3.20
11.0
10.5
10.0
9.5
9.0
8.5
8.0
7.5
7.0
6.5
6.0
5.5
5.0
4.5
4.0
3.5
3.0
2.5
2.0
1.5
1.0
0.5
0.0
ppm

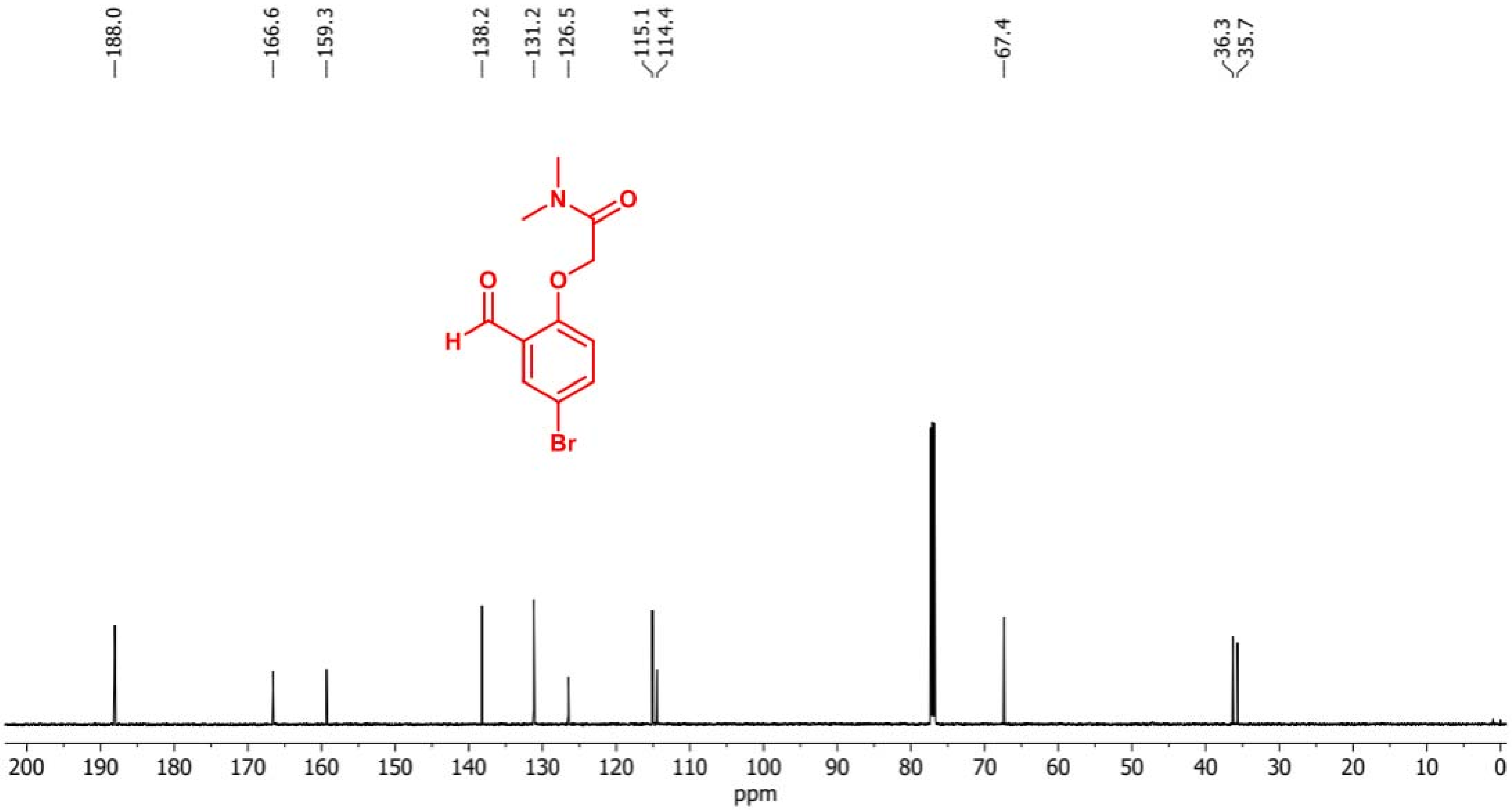

188.0
166.6
159.3
138.2
131.2
126.5
115.1
114.4
67.4
36.3
35.7
O
N
O
O
H
Br
200 190 180 170 160 150 140 130 120 110 100 90 80 70 60 50 40 30 20 10 0
ppm

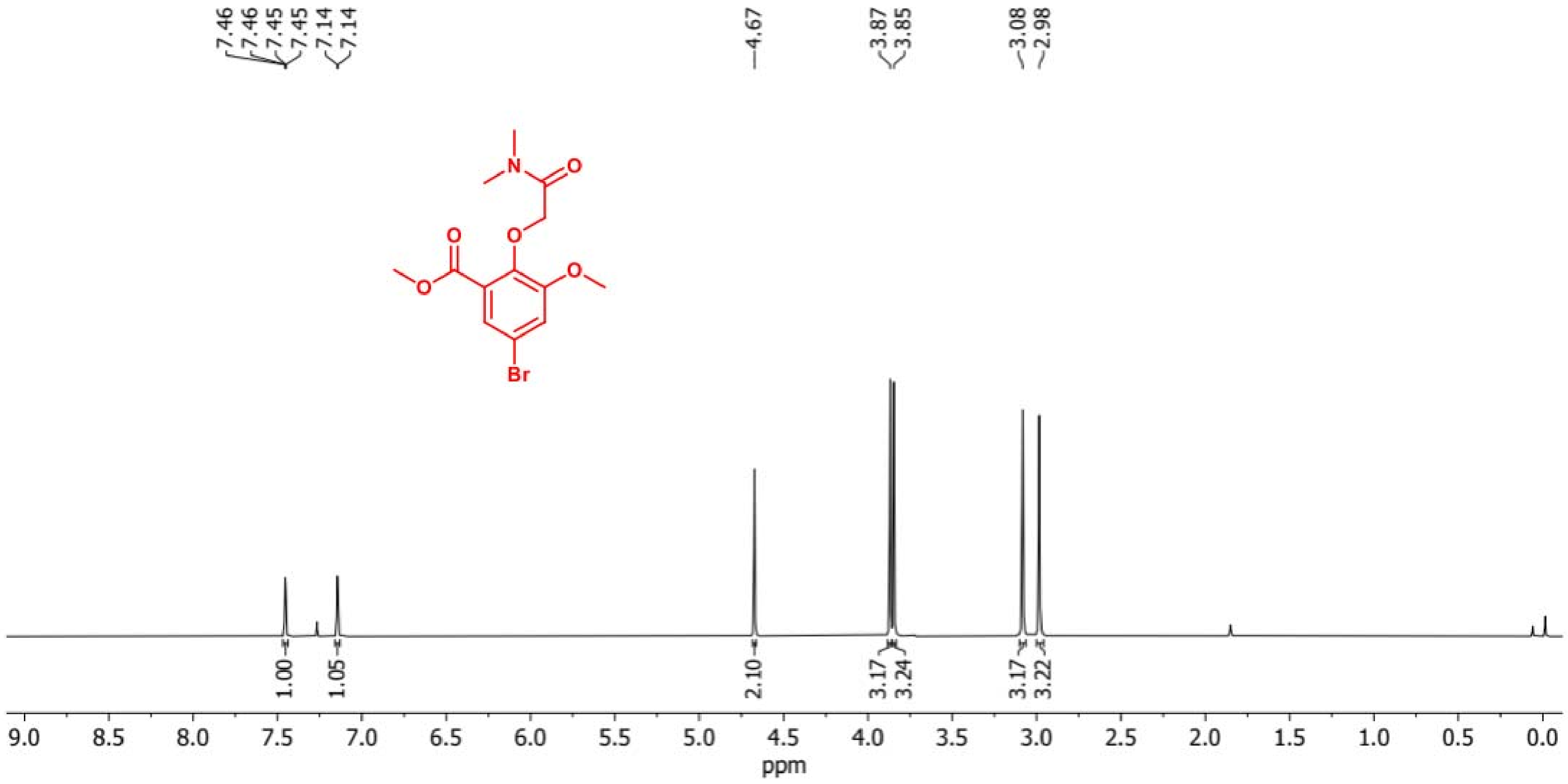
7.46
7.46
7.45
7.45
7.14
7.14
4.67
3.87
3.85
3.08
2.98
N
O
O
O
O
O
Br
1.00
1.05
2.10
3.17
3.24
3.17
3.22
9.0
8.5
8.0
7.5
7.0
6.5
6.0
5.5
5.0
4.5
4.0
3.5
3.0
2.5
2.0
1.5
1.0
0.5
0.0
ppm

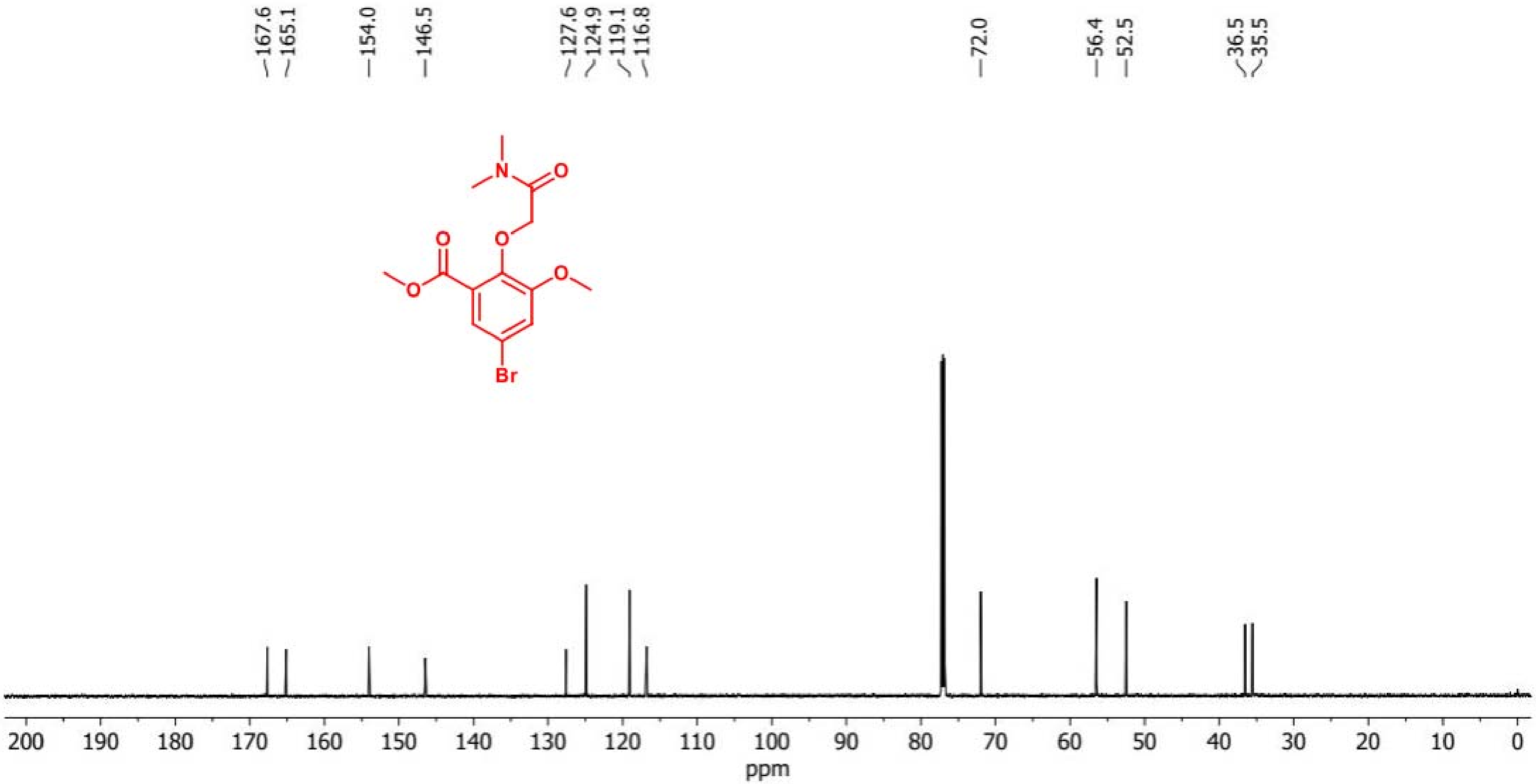
167.6
165.1
154.0
146.5
127.6
124.9
119.1
116.8
72.0
56.4
52.5
36.5
35.5
N
O
O
O
O
O
Br
200
190
180
170
160
150
140
130
120
110
100
90
80
70
60
50
40
30
20
10
0
ppm

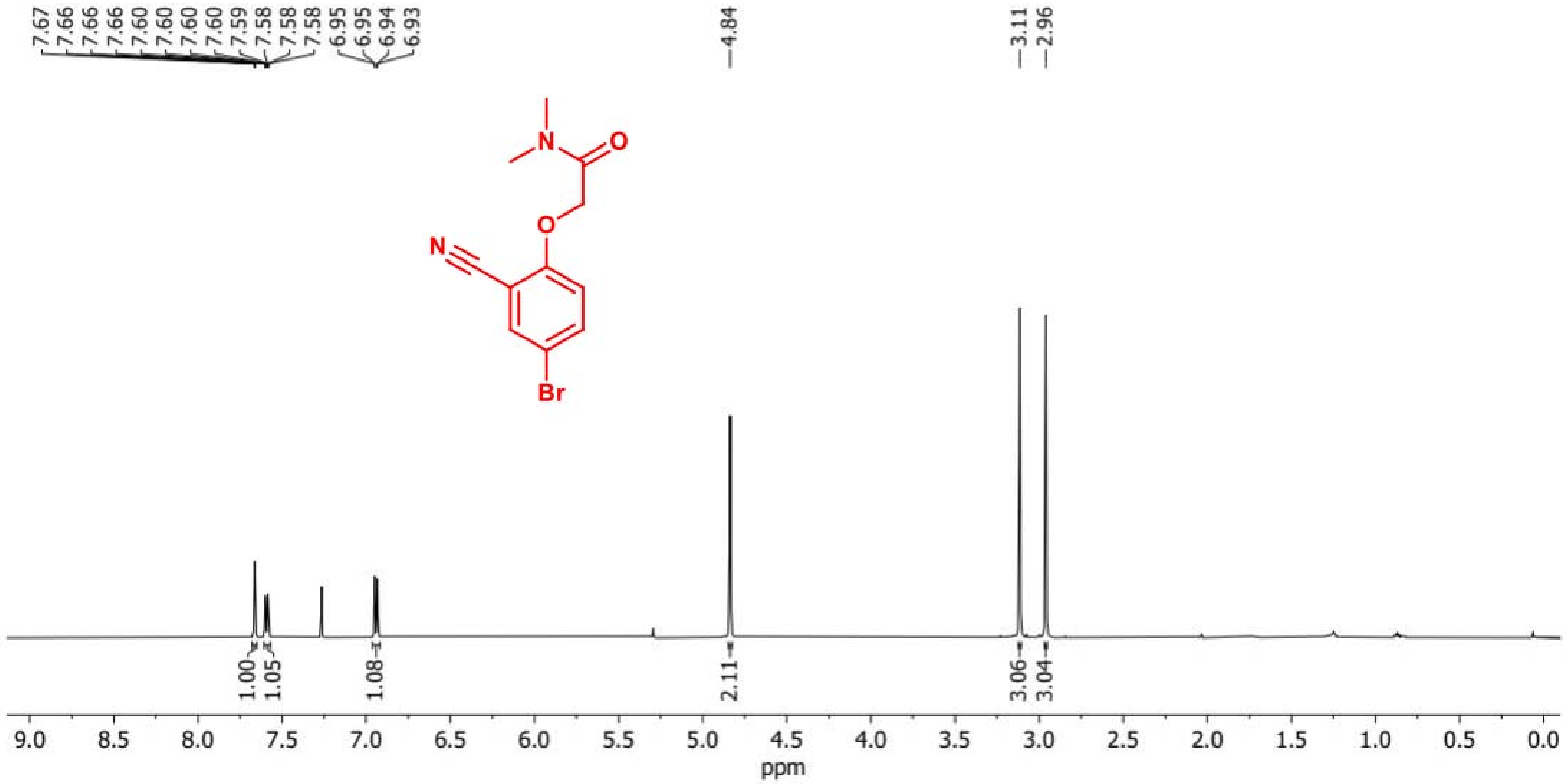

7.67
7.66
7.66
7.66
7.60
7.60
7.60
7.60
7.59
7.58
7.58
7.58
6.95
6.95
6.94
6.93
4.84
3.11
2.96
N
O
O
N
Br
1.00
1.05
1.08
2.11
3.06
3.04
9.0
8.5
8.0
7.5
7.0
6.5
6.0
5.5
5.0
4.5
4.0
3.5
3.0
2.5
2.0
1.5
1.0
0.5
0.0
ppm

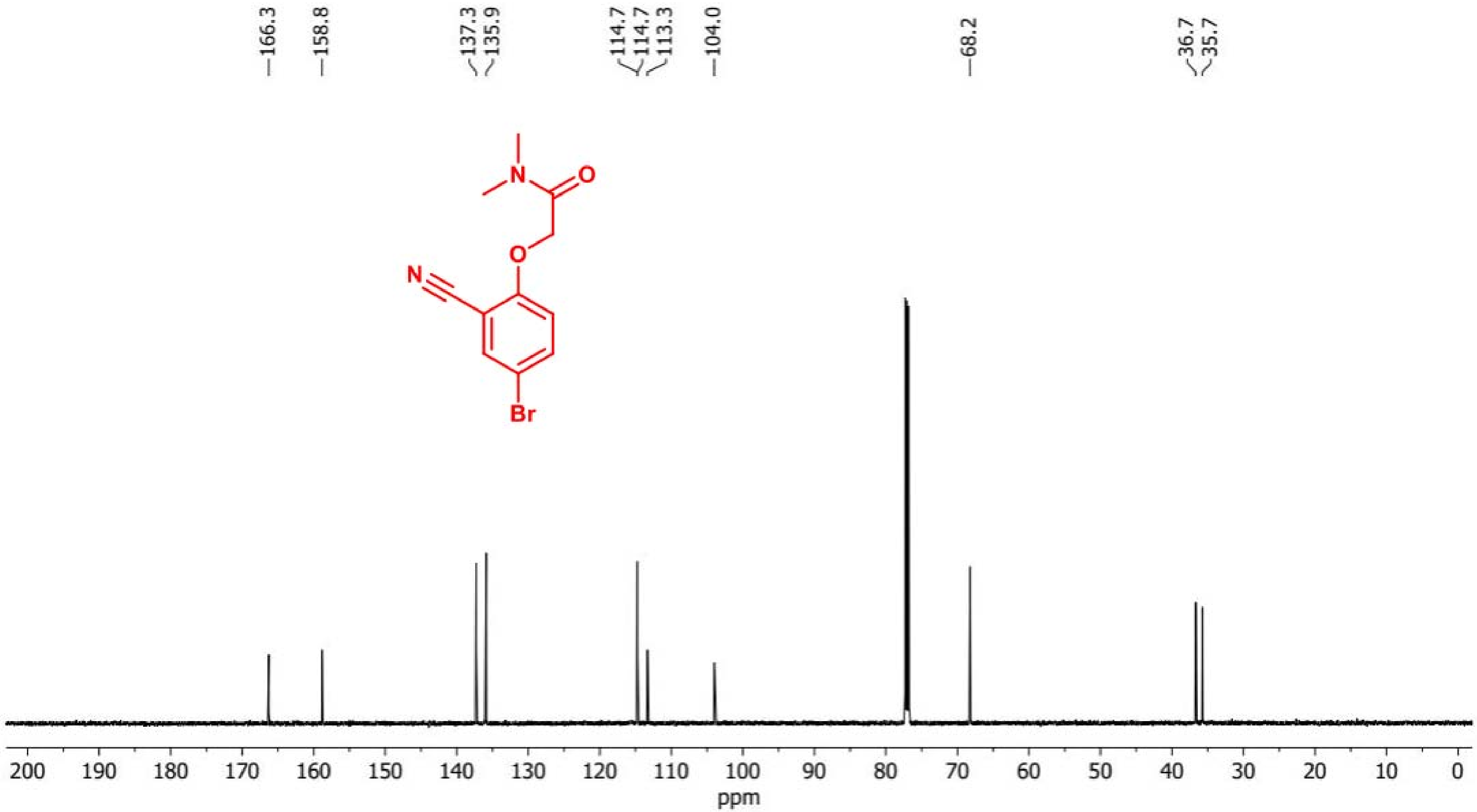

166.3
158.8
137.3
135.9
114.7
114.7
113.3
104.0
68.2
36.7
35.7
N
O
O
N
Br
200
190
180
170
160
150
140
130
120
110
100
90
80
70
60
50
40
30
20
10
0
ppm

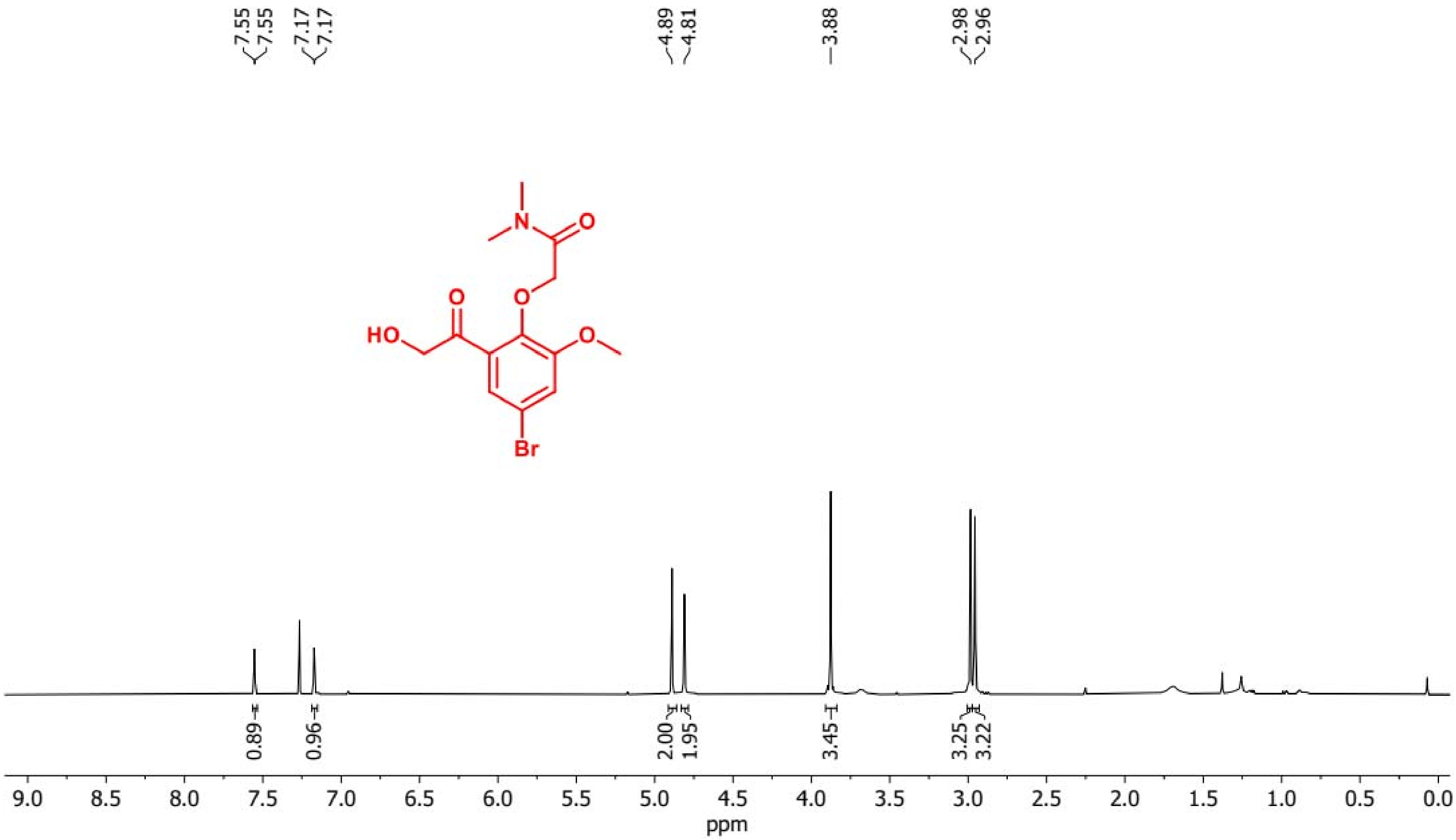
7.55
7.55
7.17
7.17
4.89
4.81
3.88
2.98
2.96
HO
O
O
N
O
O
Br
0.89
0.96
2.00
1.95
3.45
3.25
3.22
9.0 8.5 8.0 7.5 7.0 6.5 6.0 5.5 5.0 4.5 4.0 3.5 3.0 2.5 2.0 1.5 1.0 0.5 0.0
ppm

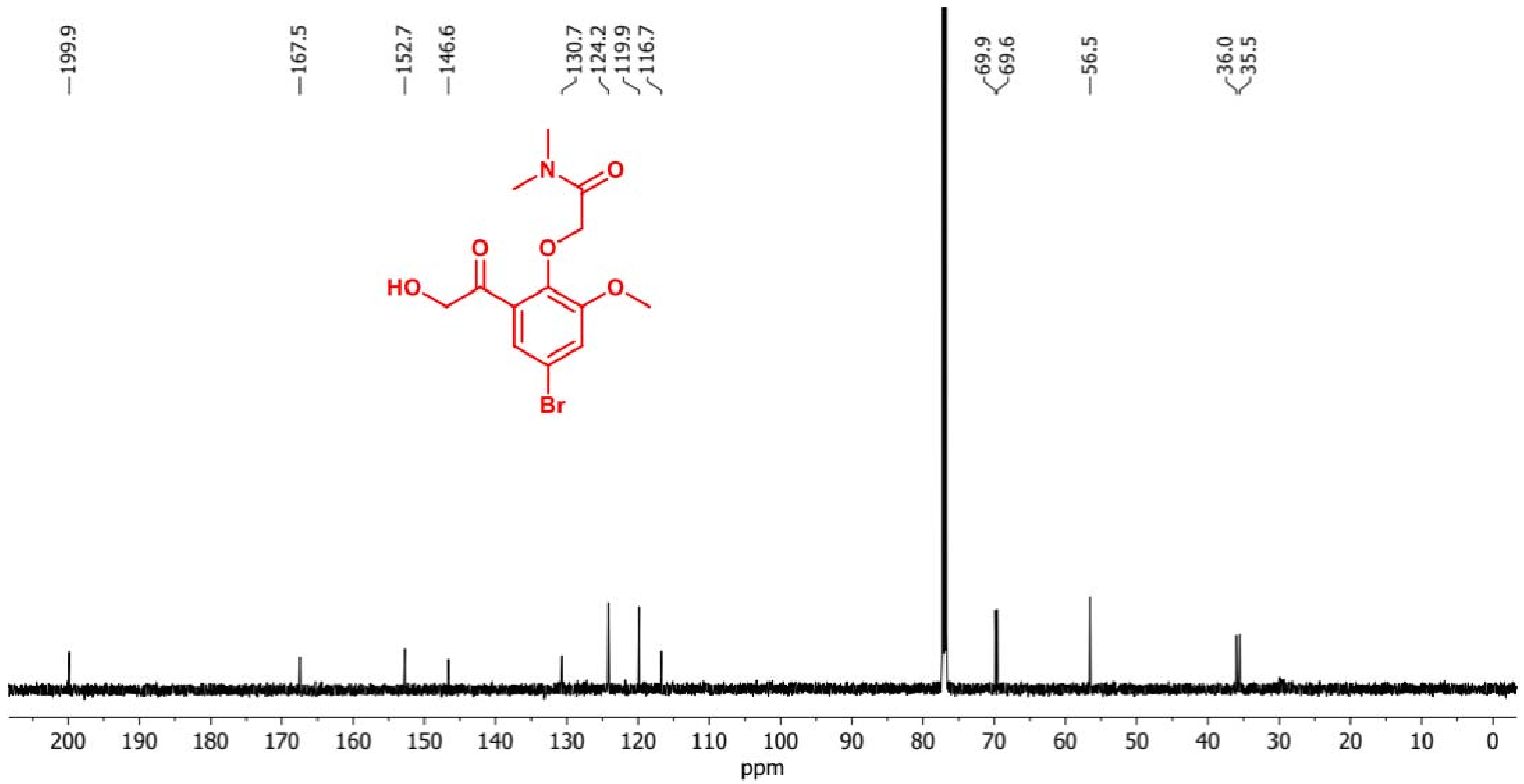

199.9
167.5
152.7
146.6
130.7
124.2
119.9
116.7
69.9
69.6
56.5
36.0
35.5
N
O
O
O
HO
O
Br
200
190
180
170
160
150
140
130
120
110
100
90
80
70
60
50
40
30
20
10
0
ppm

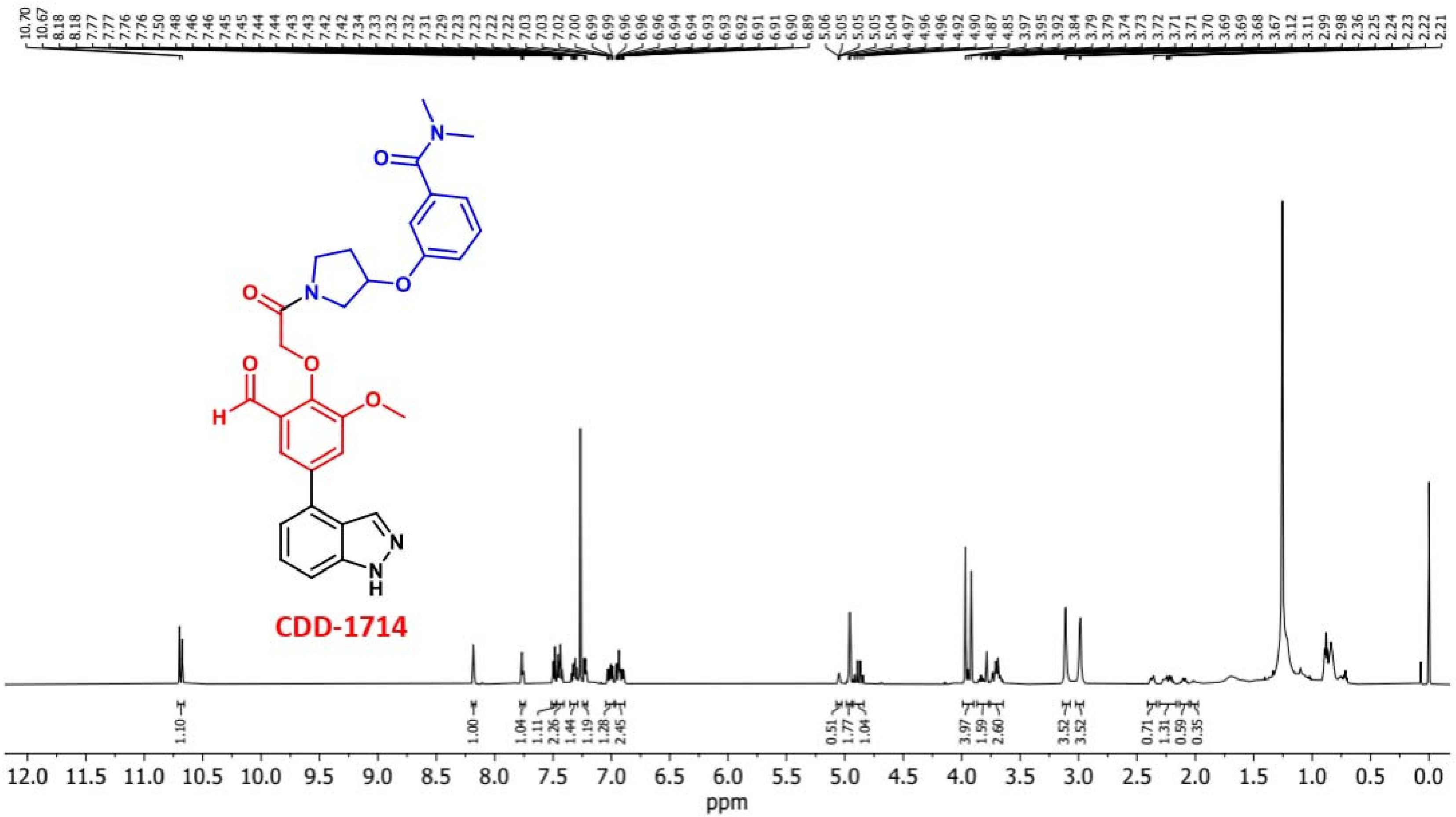
CDD-1714
ppm
12.0 11.5 11.0 10.5 10.0 9.5 9.0 8.5 8.0 7.5 7.0 6.5 6.0 5.5 5.0 4.5 4.0 3.5 3.0 2.5 2.0 1.5 1.0 0.5 0.0

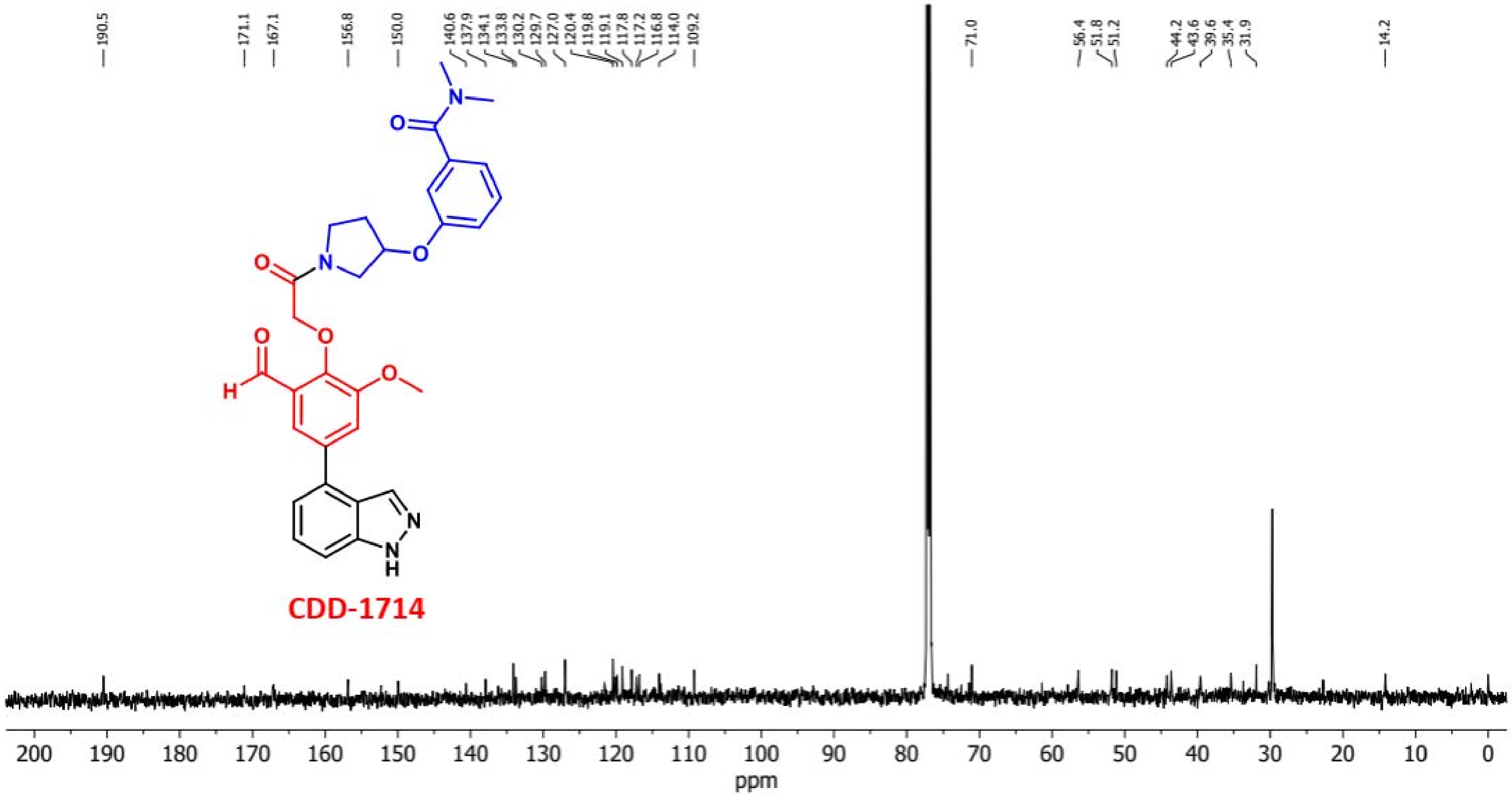
190.5
171.1
167.1
156.8
150.0
140.6
137.9
134.1
133.8
130.2
129.7
127.0
120.4
119.8
119.1
117.8
117.2
116.8
114.0
109.2
71.0
56.4
51.8
51.2
44.2
43.6
39.6
35.4
31.9
14.2
CDD-1714
200 190 180 170 160 150 140 130 120 110 100 90 80 70 60 50 40 30 20 10 0
ppm

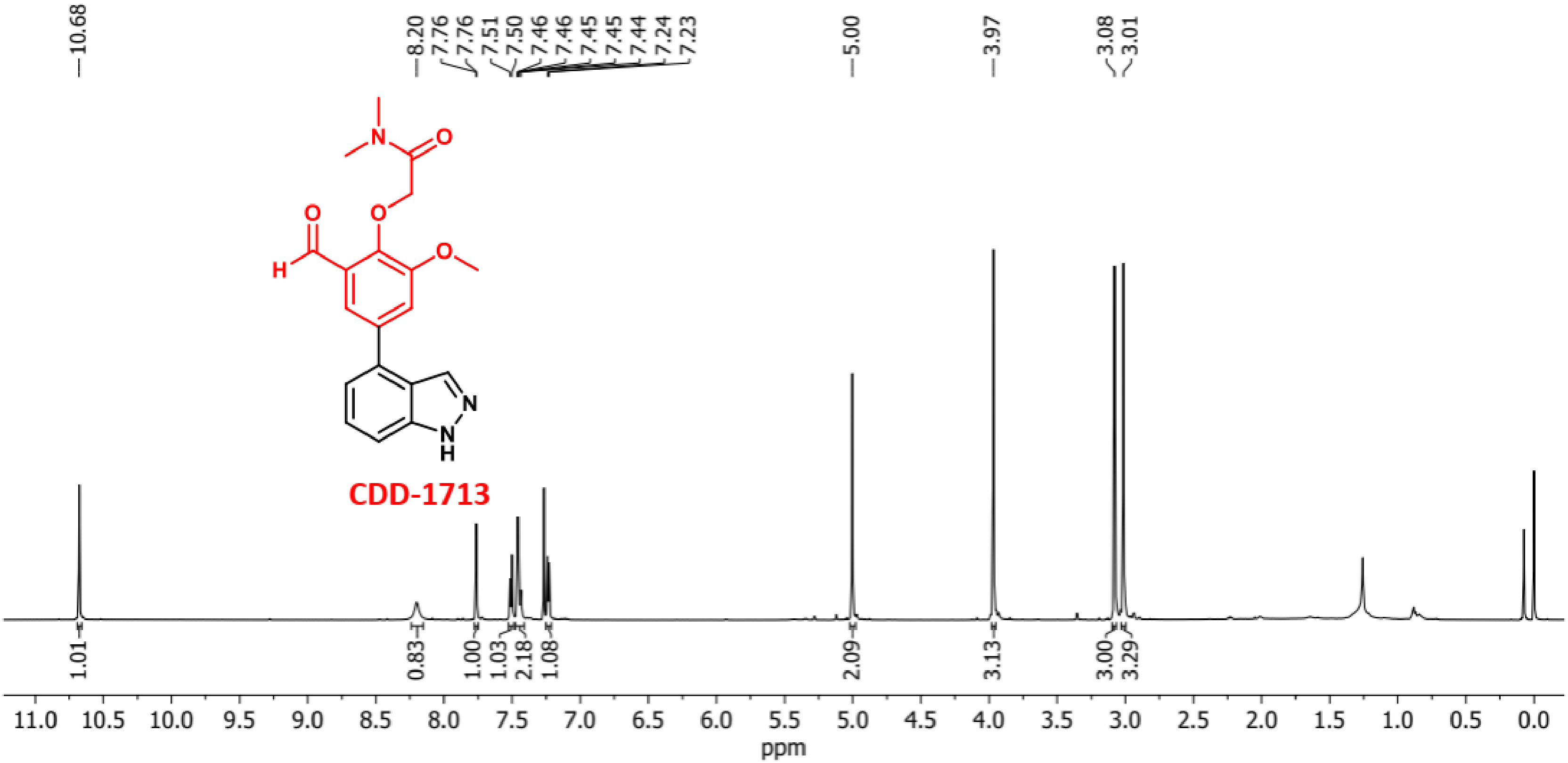
10.68
8.20
7.76
7.76
7.51
7.50
7.46
7.46
7.45
7.45
7.44
7.24
7.23
5.00
3.97
3.08
3.01
CDD-1713
1.01
0.83
1.00
1.03
2.18
1.08
2.09
3.13
3.00
3.29
11.0 10.5 10.0 9.5 9.0 8.5 8.0 7.5 7.0 6.5 6.0 5.5 5.0 4.5 4.0 3.5 3.0 2.5 2.0 1.5 1.0 0.5 0.0
ppm

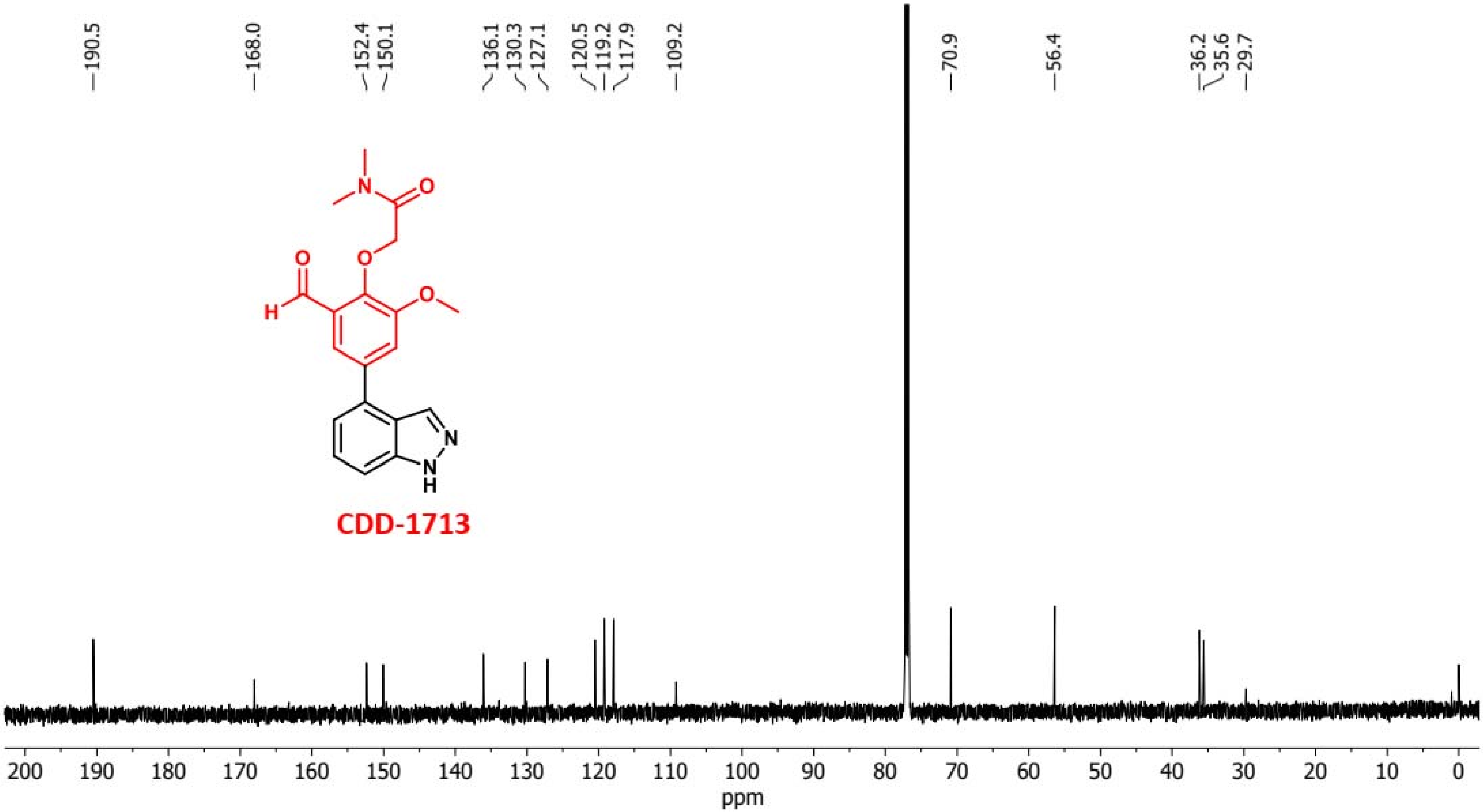
190.5
168.0
152.4
150.1
136.1
130.3
127.1
120.5
119.2
117.9
109.2
70.9
56.4
36.2
35.6
29.7
O
N
O
H
O
O
N
N
H
CDD-1713
200 190 180 170 160 150 140 130 120 110 100 90 80 70 60 50 40 30 20 10 0
ppm

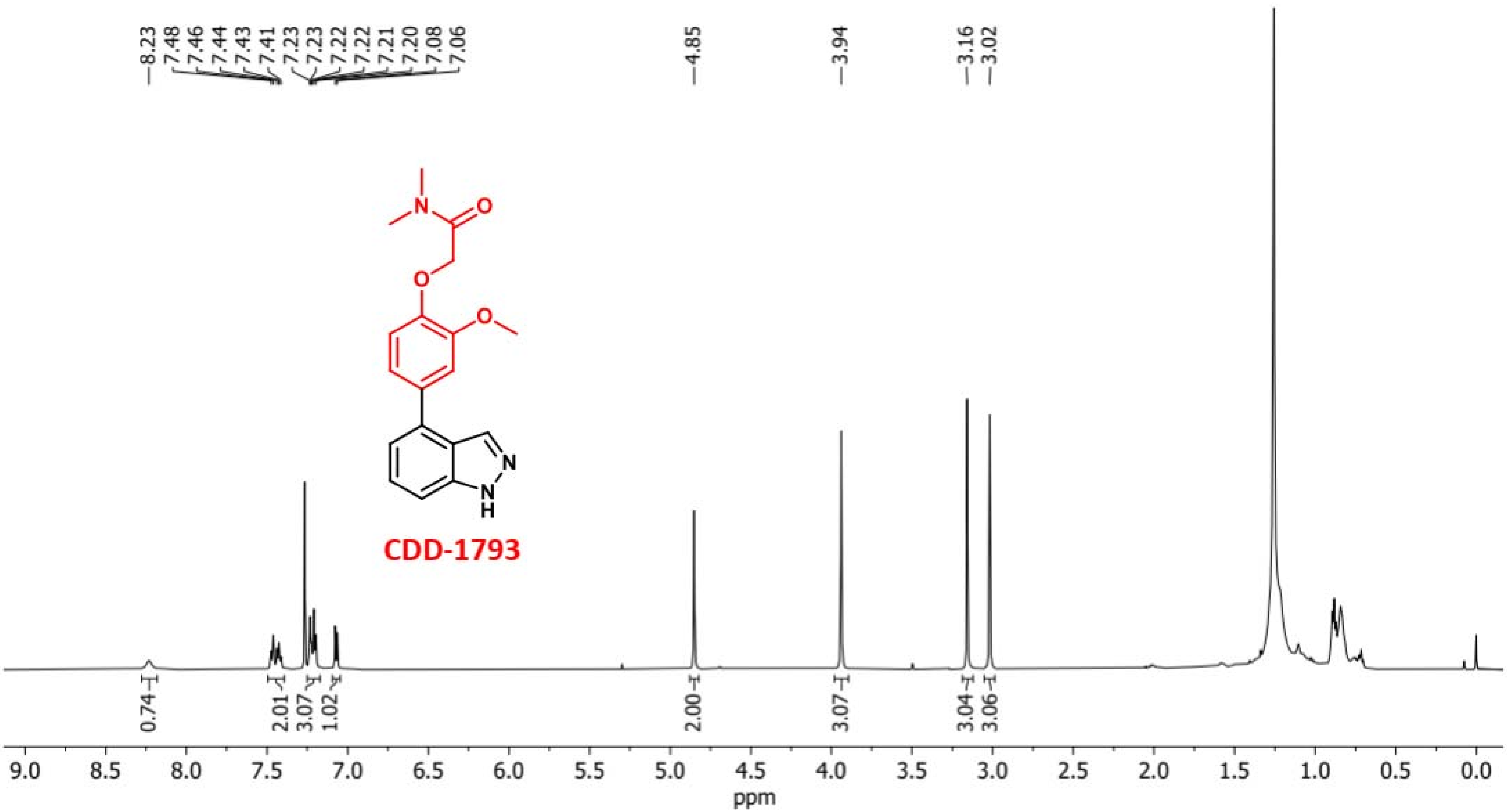

8.23
7.48
7.46
7.44
7.43
7.41
7.23
7.23
7.22
7.22
7.21
7.20
7.08
7.06
4.85
3.94
3.16
3.02
CDD-1793
0.74
2.01
3.07
1.02
2.00
3.07
3.04
3.06
9.0
8.5
8.0
7.5
7.0
6.5
6.0
5.5
5.0
4.5
4.0
3.5
3.0
2.5
2.0
1.5
1.0
0.5
0.0
ppm

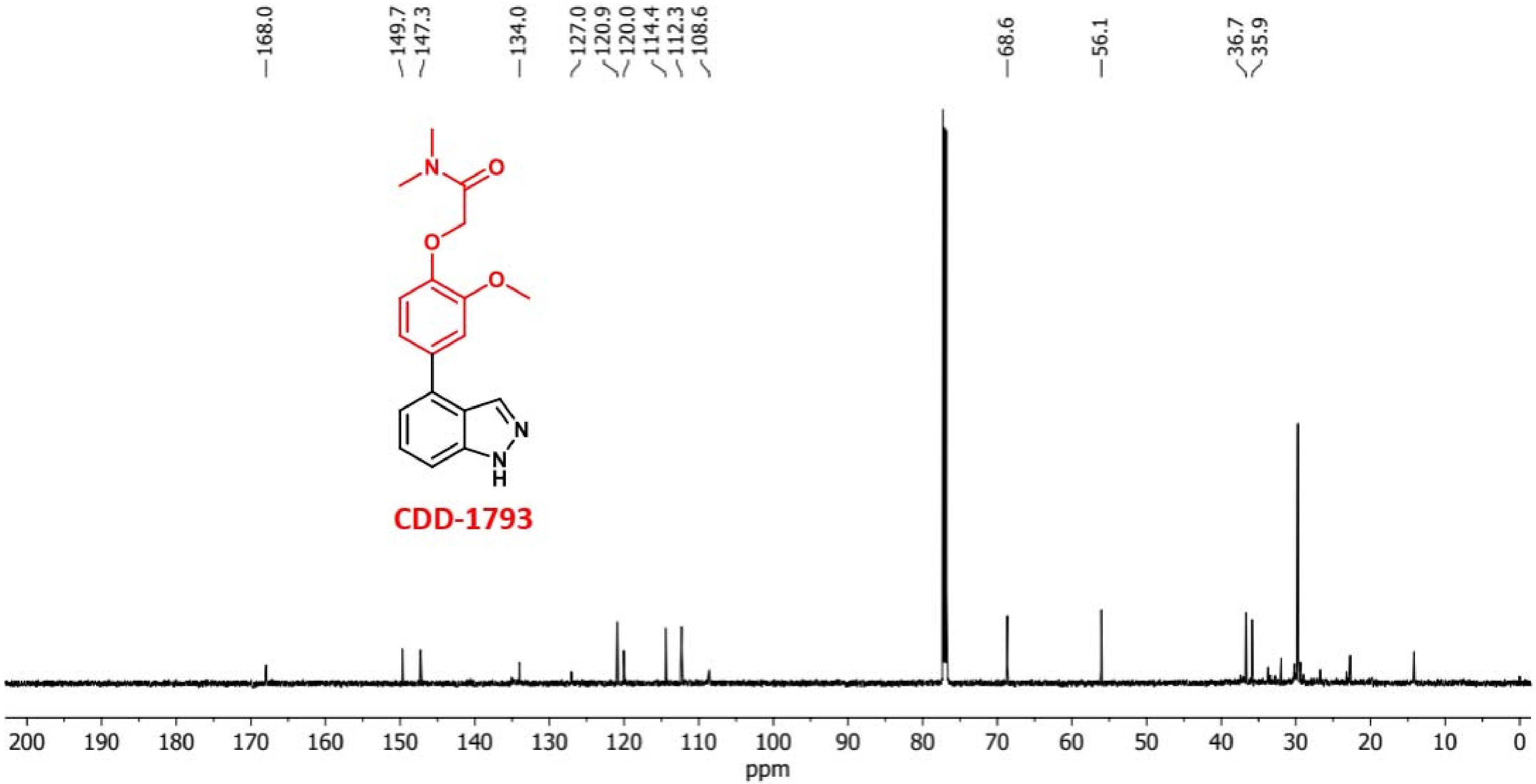

168.0
149.7
147.3
134.0
127.0
120.9
120.0
114.4
112.3
108.6
68.6
56.1
36.7
35.9
O
N
O
O
N
N
H
CDD-1793
200
190
180
170
160
150
140
130
120
110
100
90
80
70
60
50
40
30
20
10
0
ppm

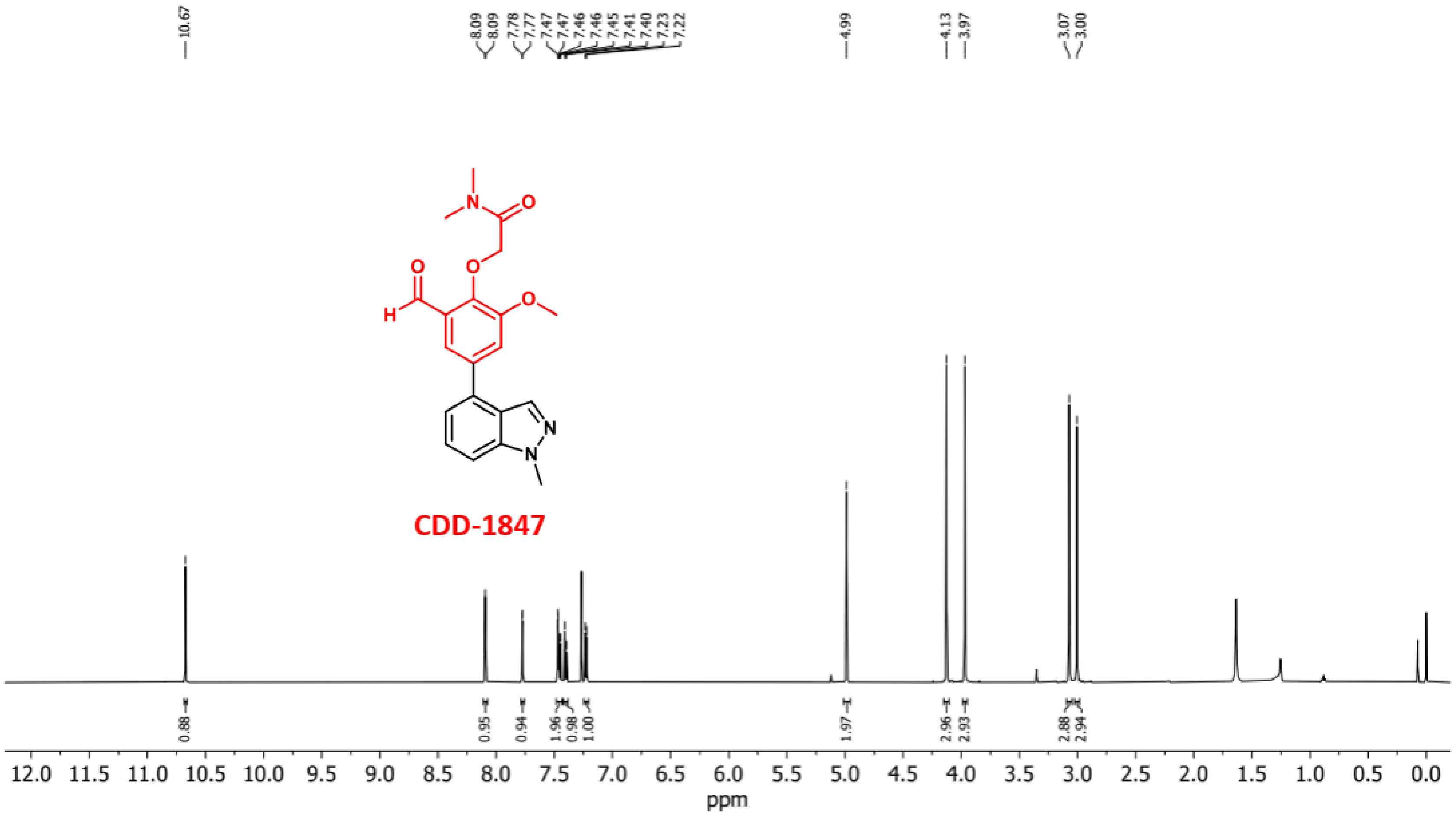

10.67
8.09
8.09
7.78
7.77
7.47
7.47
7.46
7.46
7.45
7.41
7.40
7.23
7.22
4.99
4.13
3.97
3.07
3.00
CDD-1847
0.88
0.95
0.94
1.96
0.98
1.00
1.97
2.96
2.93
2.88
2.94
12.0 11.5 11.0 10.5 10.0 9.5 9.0 8.5 8.0 7.5 7.0 6.5 6.0 5.5 5.0 4.5 4.0 3.5 3.0 2.5 2.0 1.5 1.0 0.5 0.0
ppm

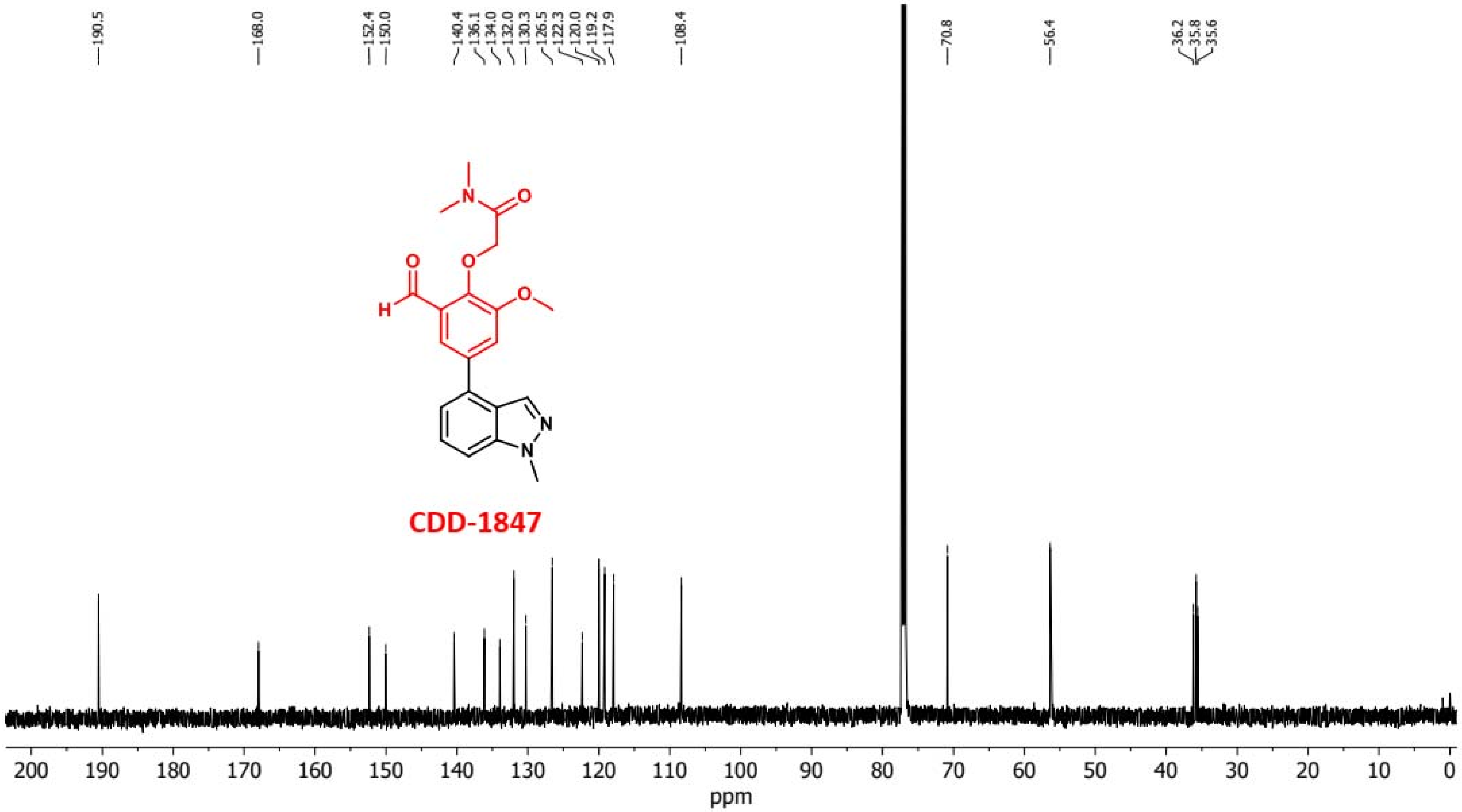

190.5
168.0
152.4
150.0
140.4
136.1
134.0
132.0
130.3
126.5
122.3
120.0
119.2
117.9
108.4
70.8
56.4
36.2
35.8
35.6
CDD-1847
200 190 180 170 160 150 140 130 120 110 100 90 80 70 60 50 40 30 20 10 0
ppm

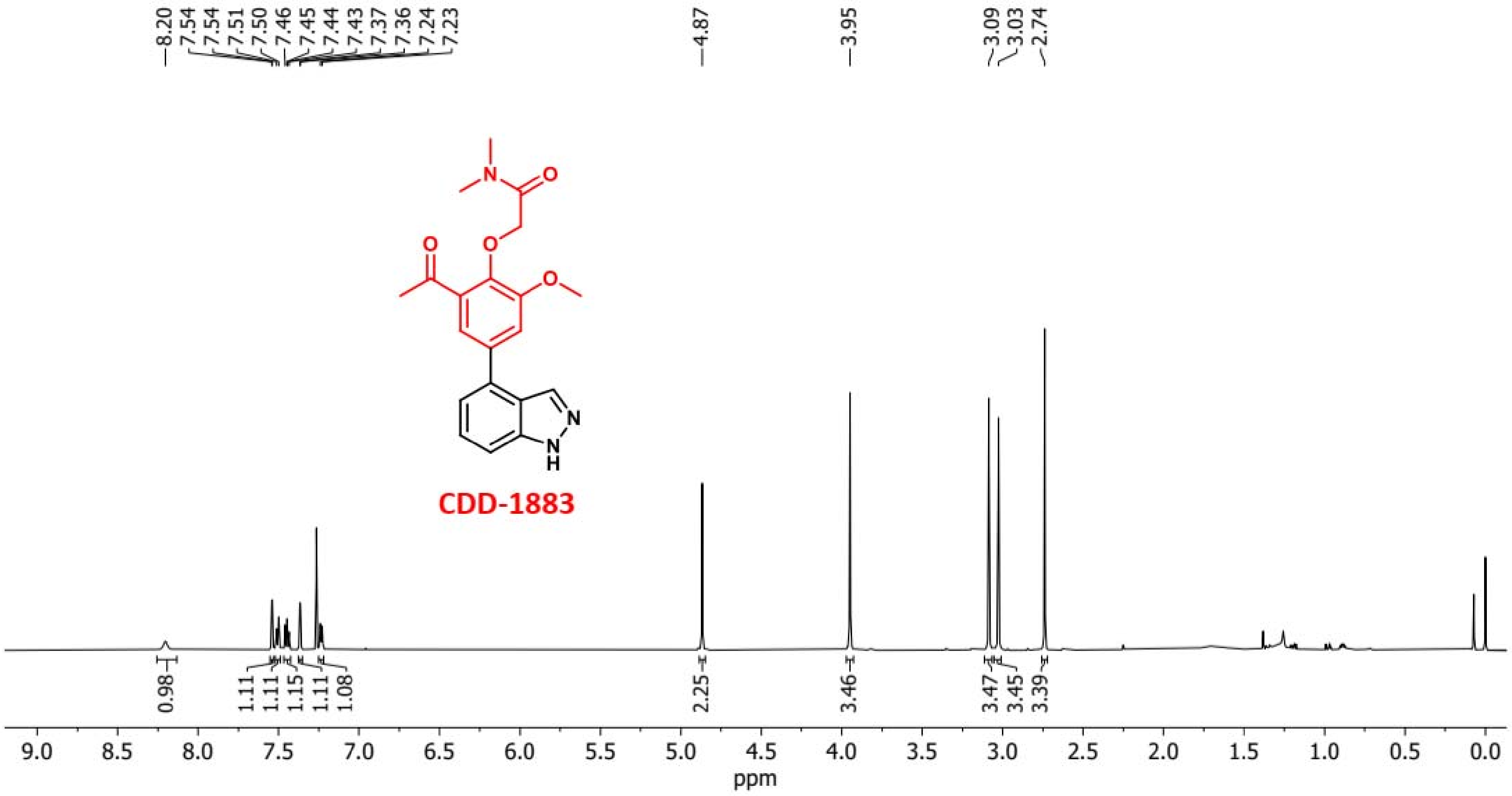
8.20
7.54
7.54
7.51
7.50
7.46
7.45
7.44
7.43
7.37
7.36
7.24
7.23
4.87
3.95
3.09
3.03
2.74
CDD-1883
0.98
1.11
1.11
1.15
1.11
1.08
2.25
3.46
3.47
3.45
3.39
9.0 8.5 8.0 7.5 7.0 6.5 6.0 5.5 5.0 4.5 4.0 3.5 3.0 2.5 2.0 1.5 1.0 0.5 0.0
ppm

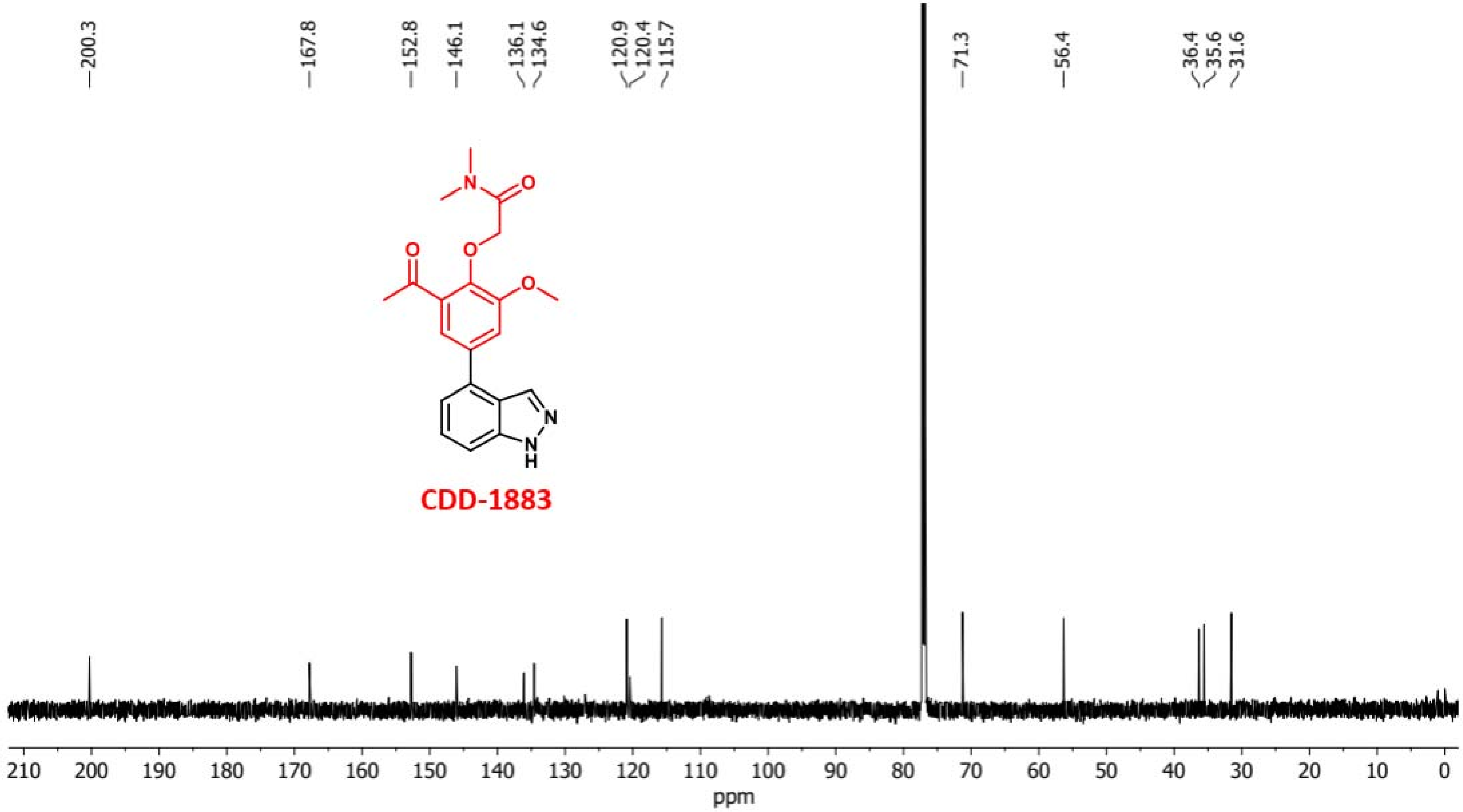

200.3
167.8
152.8
146.1
136.1
134.6
120.9
120.4
115.7
71.3
56.4
36.4
35.6
31.6
O
N
O
O
O
O
N
N
H
CDD-1883
210
200
190
180
170
160
150
140
130
120
110
100
90
80
70
60
50
40
30
20
10
0
ppm

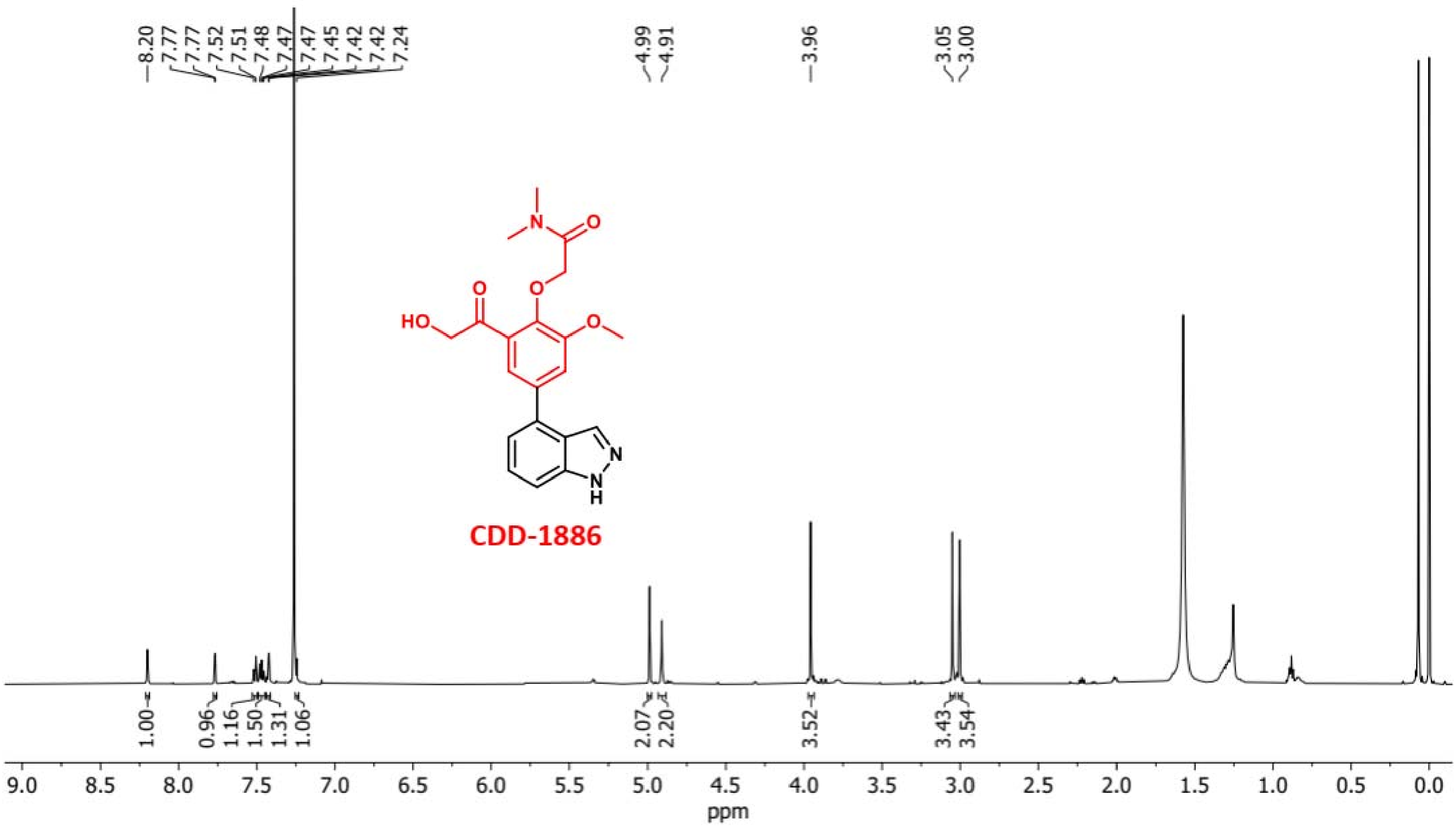
8.20
7.77
7.77
7.52
7.51
7.48
7.47
7.47
7.45
7.42
7.42
7.24
4.99
4.91
3.96
3.05
3.00
HO
O
O
O
N
O
N
N
H
CDD-1886
1.00
0.96
1.16
1.50
1.31
1.06
2.07
2.20
3.52
3.43
3.54
9.0
8.5
8.0
7.5
7.0
6.5
6.0
5.5
5.0
4.5
4.0
3.5
3.0
2.5
2.0
1.5
1.0
0.5
0.0
ppm

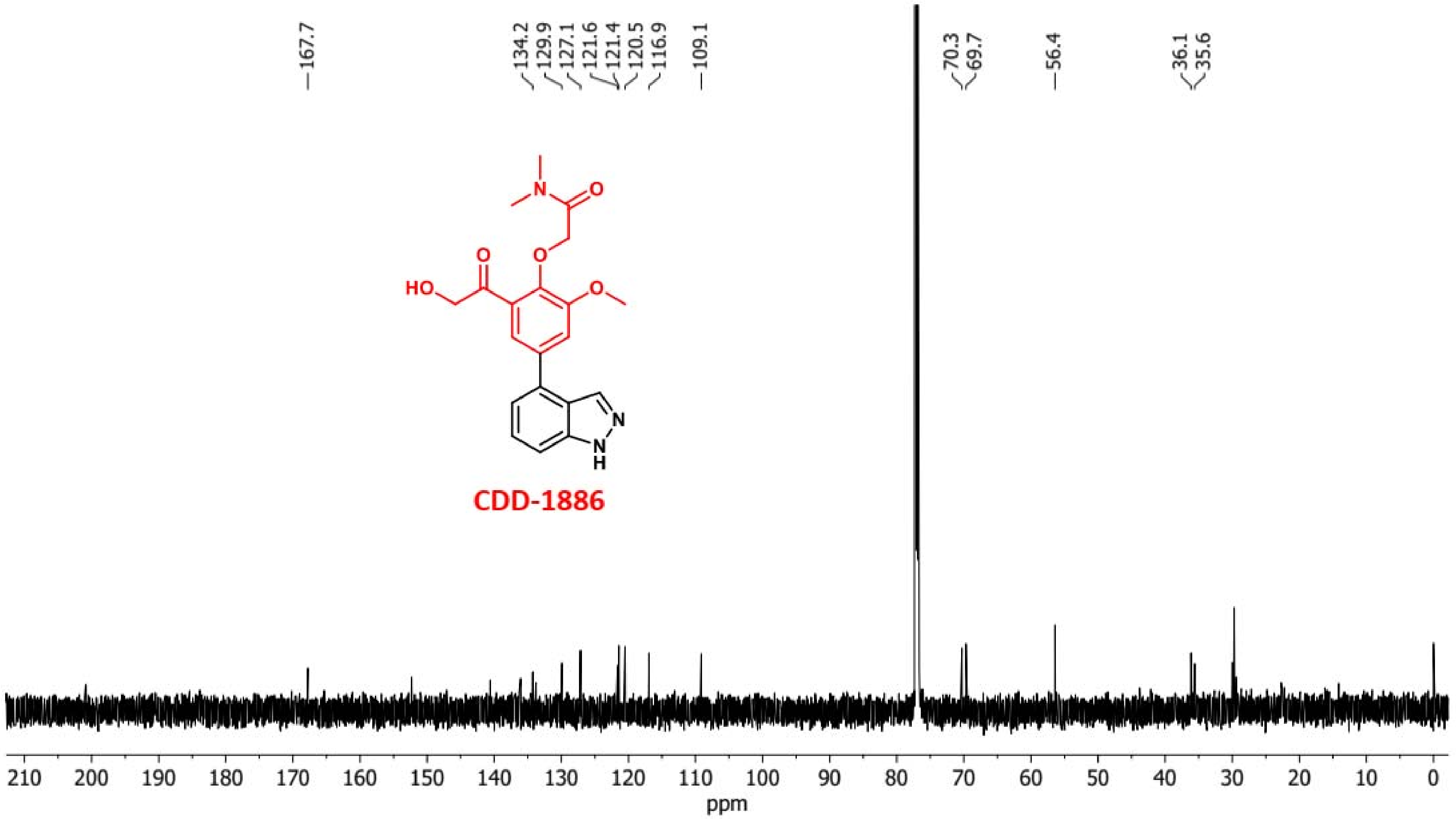
167.7
134.2
129.9
127.1
121.6
121.4
120.5
116.9
109.1
70.3
69.7
56.4
36.1
35.6
HO
O
O
N
O
O
N
N
H
CDD-1886
210 200 190 180 170 160 150 140 130 120 110 100 90 80 70 60 50 40 30 20 10 0
ppm

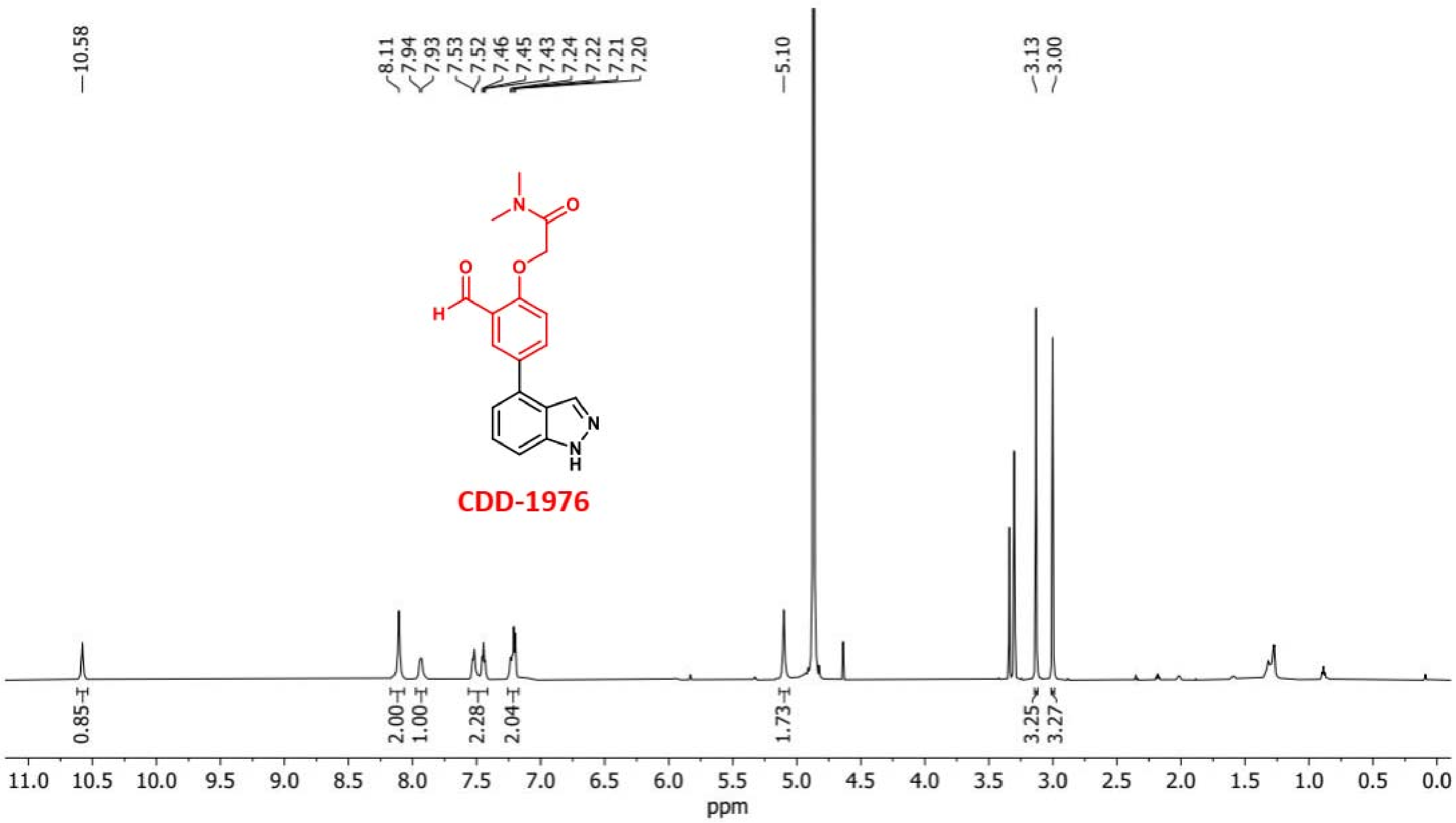
10.58
8.11
7.94
7.93
7.53
7.52
7.46
7.45
7.43
7.24
7.22
7.21
7.20
5.10
3.13
3.00
CDD-1976
0.85
2.00
1.00
2.28
2.04
1.73
3.25
3.27
11.0 10.5 10.0 9.5 9.0 8.5 8.0 7.5 7.0 6.5 6.0 5.5 5.0 4.5 4.0 3.5 3.0 2.5 2.0 1.5 1.0 0.5 0.0
ppm

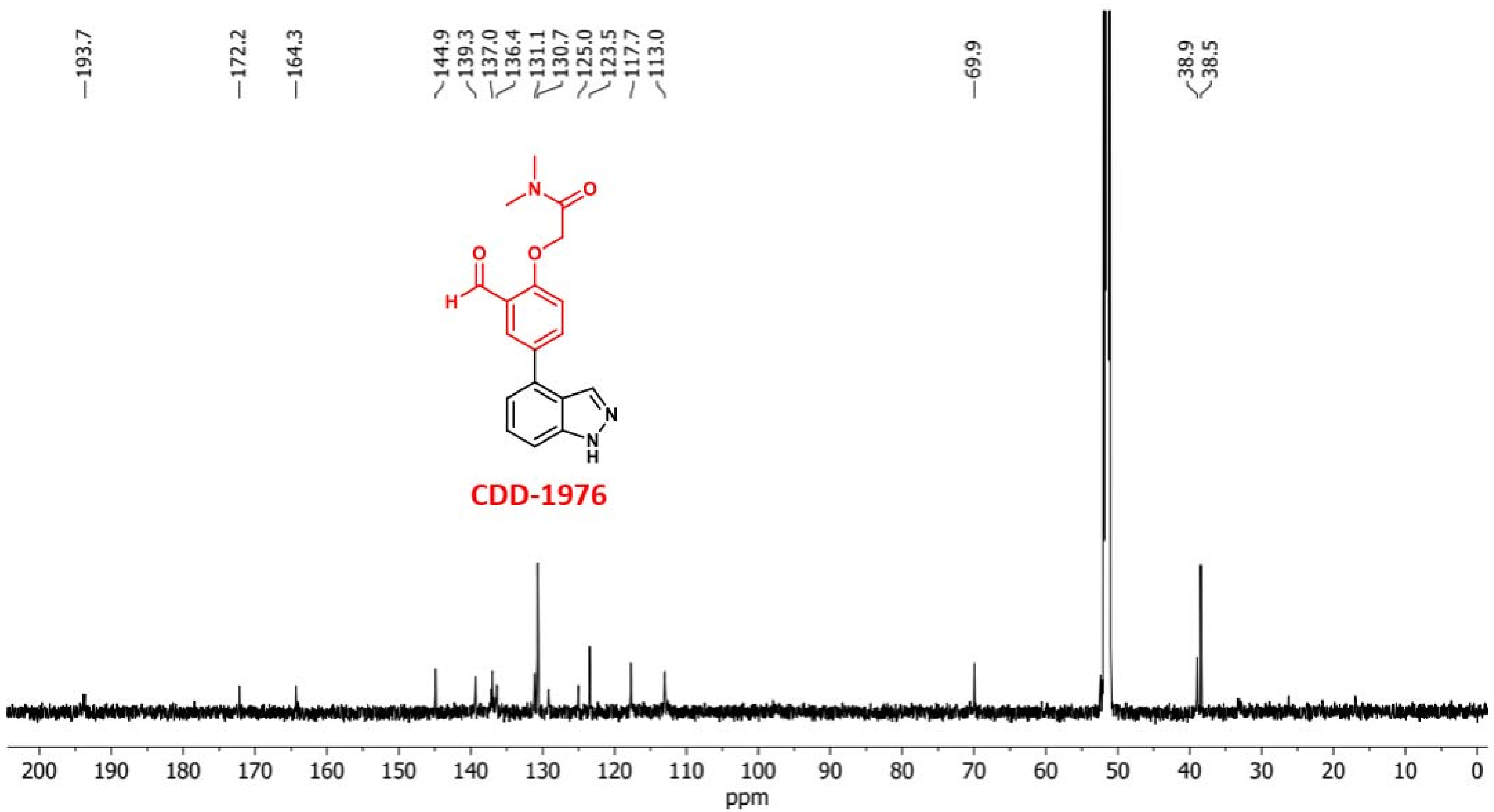
193.7
172.2
164.3
144.9
139.3
137.0
136.4
131.1
130.7
125.0
123.5
117.7
113.0
69.9
38.9
38.5
CDD-1976
200 190 180 170 160 150 140 130 120 110 100 90 80 70 60 50 40 30 20 10 0
ppm

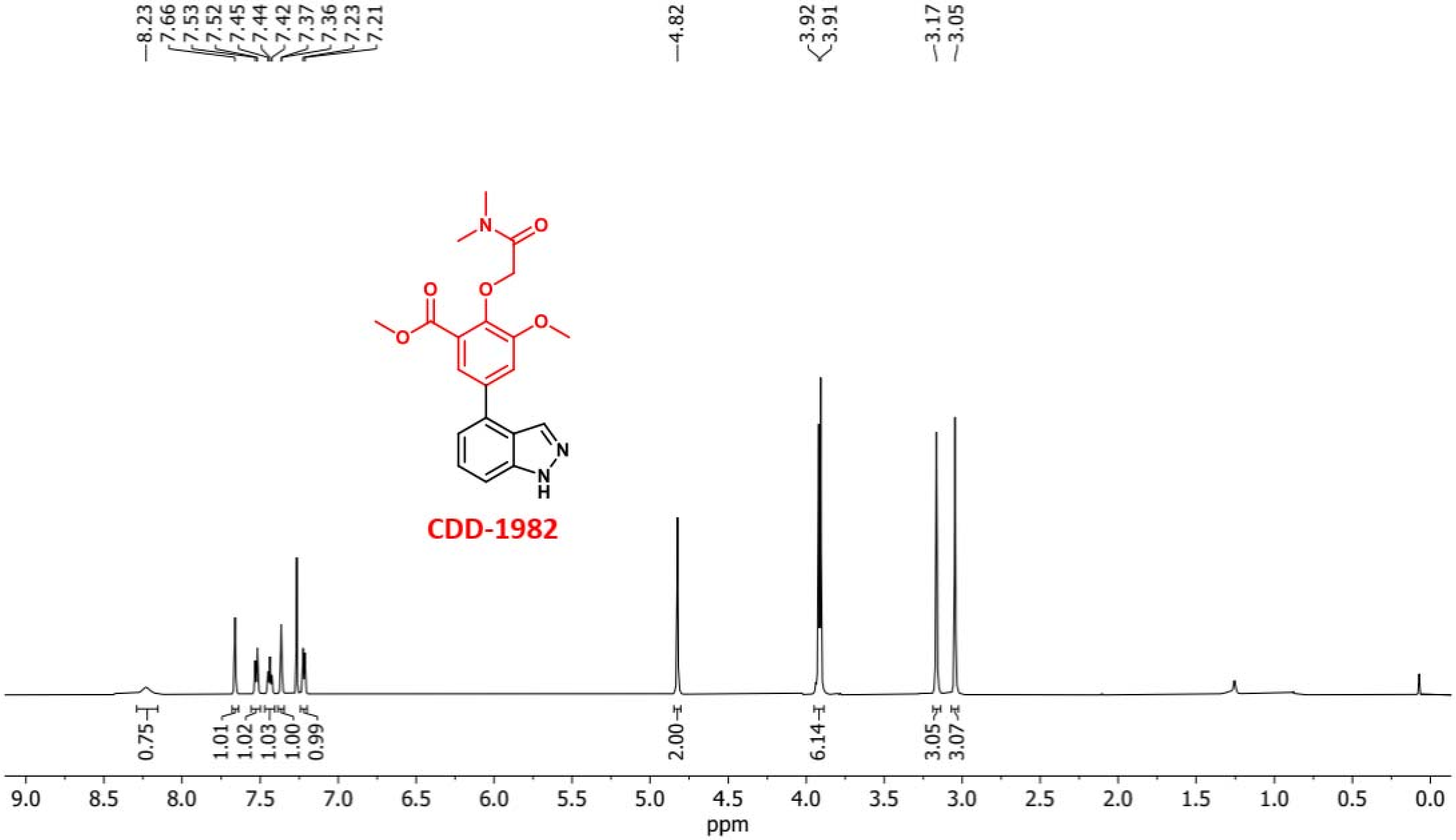

8.23
7.66
7.53
7.52
7.45
7.44
7.42
7.37
7.36
7.23
7.21
4.82
3.92
3.91
3.17
3.05
O
N
O
O
O
O
O
N
N
H
CDD-1982
0.75
1.01
1.02
1.03
1.00
0.99
2.00
6.14
3.05
3.07
9.0
8.5
8.0
7.5
7.0
6.5
6.0
5.5
5.0
4.5
4.0
3.5
3.0
2.5
2.0
1.5
1.0
0.5
0.0
ppm

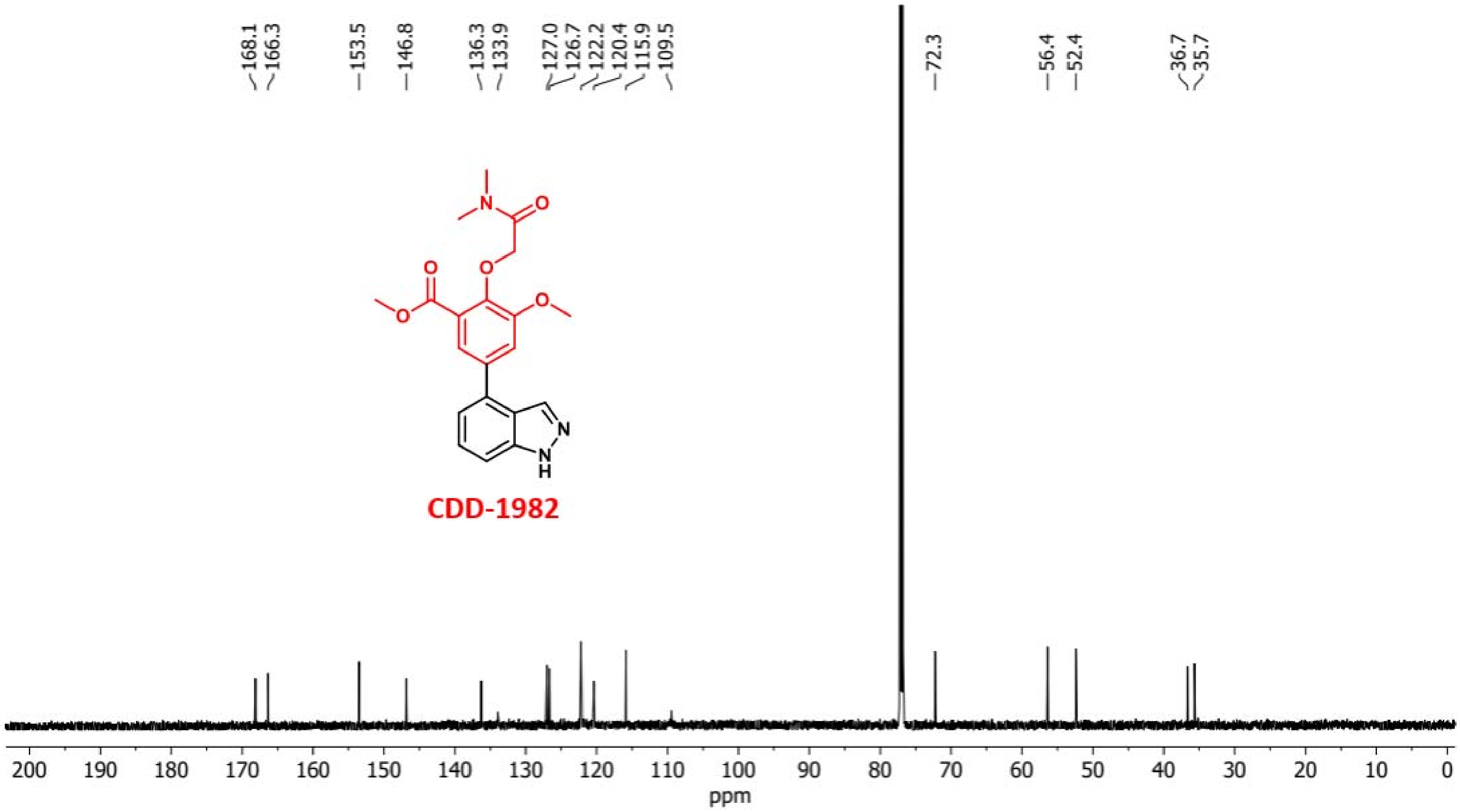
168.1
166.3
153.5
146.8
136.3
133.9
127.0
126.7
122.2
120.4
115.9
109.5
72.3
56.4
52.4
36.7
35.7
CDD-1982
200 190 180 170 160 150 140 130 120 110 100 90 80 70 60 50 40 30 20 10 0
ppm

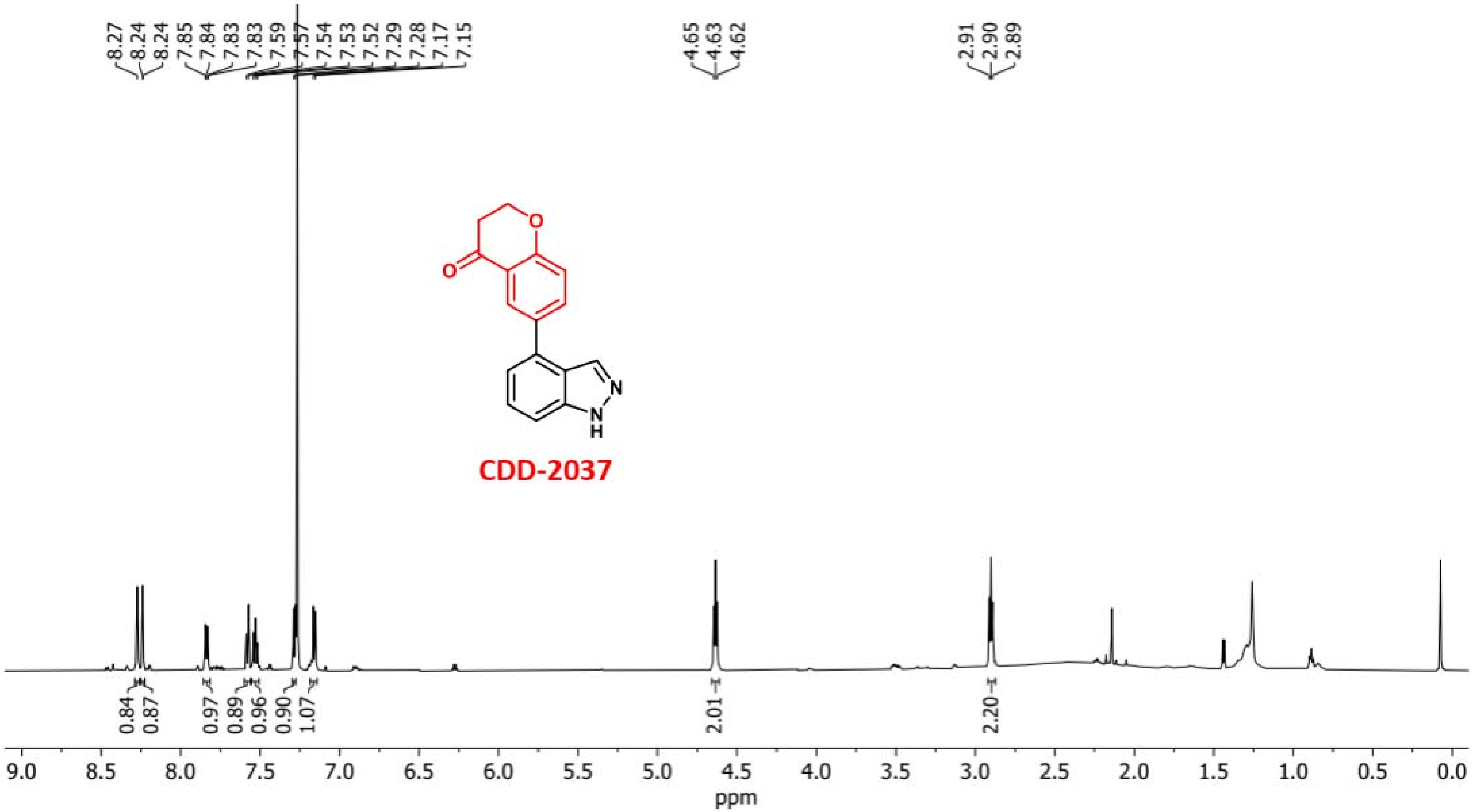

8.27
8.24
8.24
7.85
7.84
7.83
7.83
7.59
7.57
7.54
7.53
7.52
7.29
7.28
7.17
7.15
4.65
4.63
4.62
2.91
2.90
2.89
O
O
N
N
H
CDD-2037
0.84
0.87
0.97
0.89
0.96
0.90
1.07
2.01
2.20
9.0
8.5
8.0
7.5
7.0
6.5
6.0
5.5
5.0
4.5
4.0
3.5
3.0
2.5
2.0
1.5
1.0
0.5
0.0
ppm

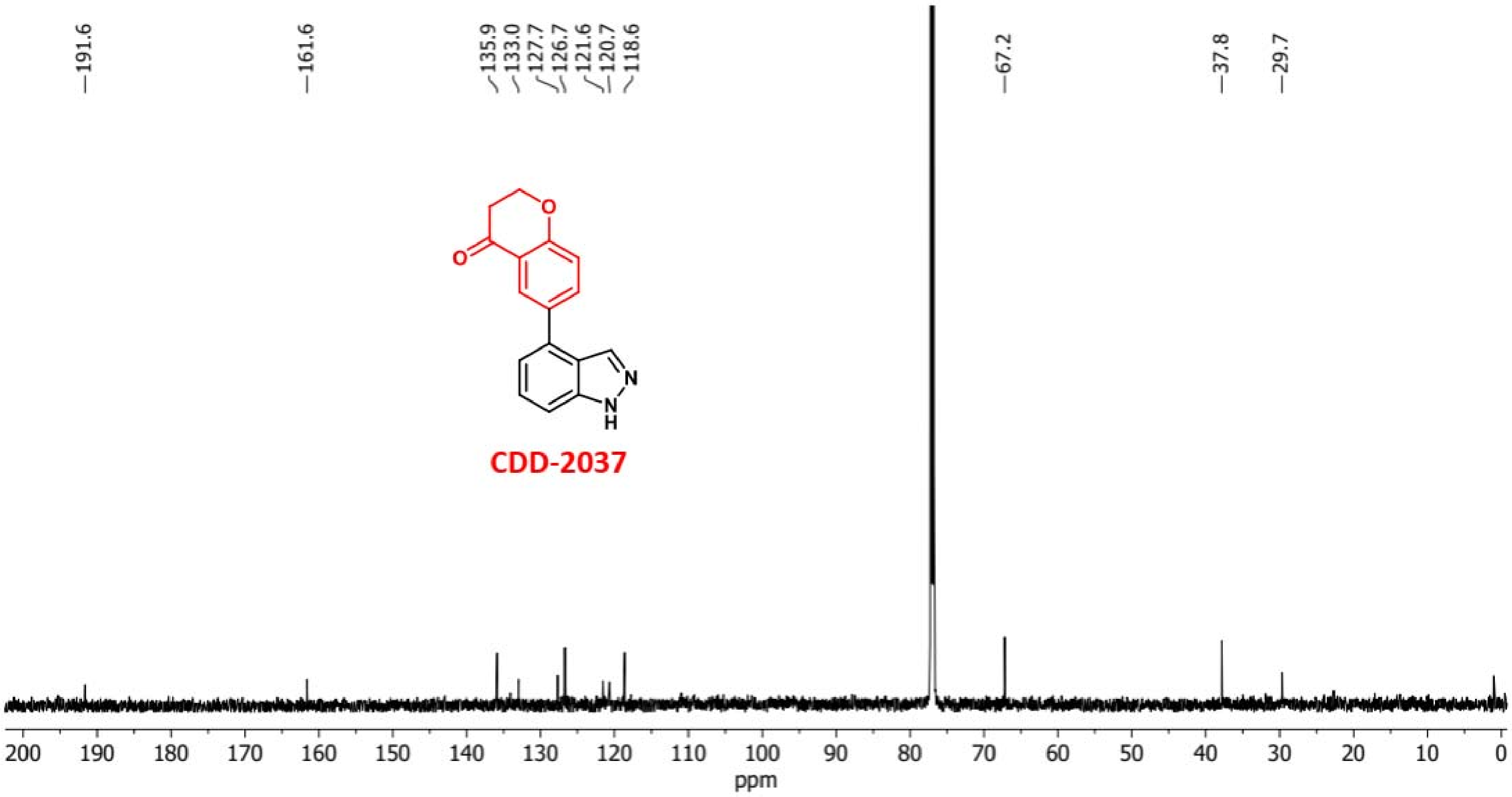

191.6
161.6
135.9
133.0
127.7
126.7
121.6
120.7
118.6
67.2
37.8
29.7
O
O
N
N
H
CDD-2037
200
190
180
170
160
150
140
130
120
110
100
90
80
70
60
50
40
30
20
10
0
ppm

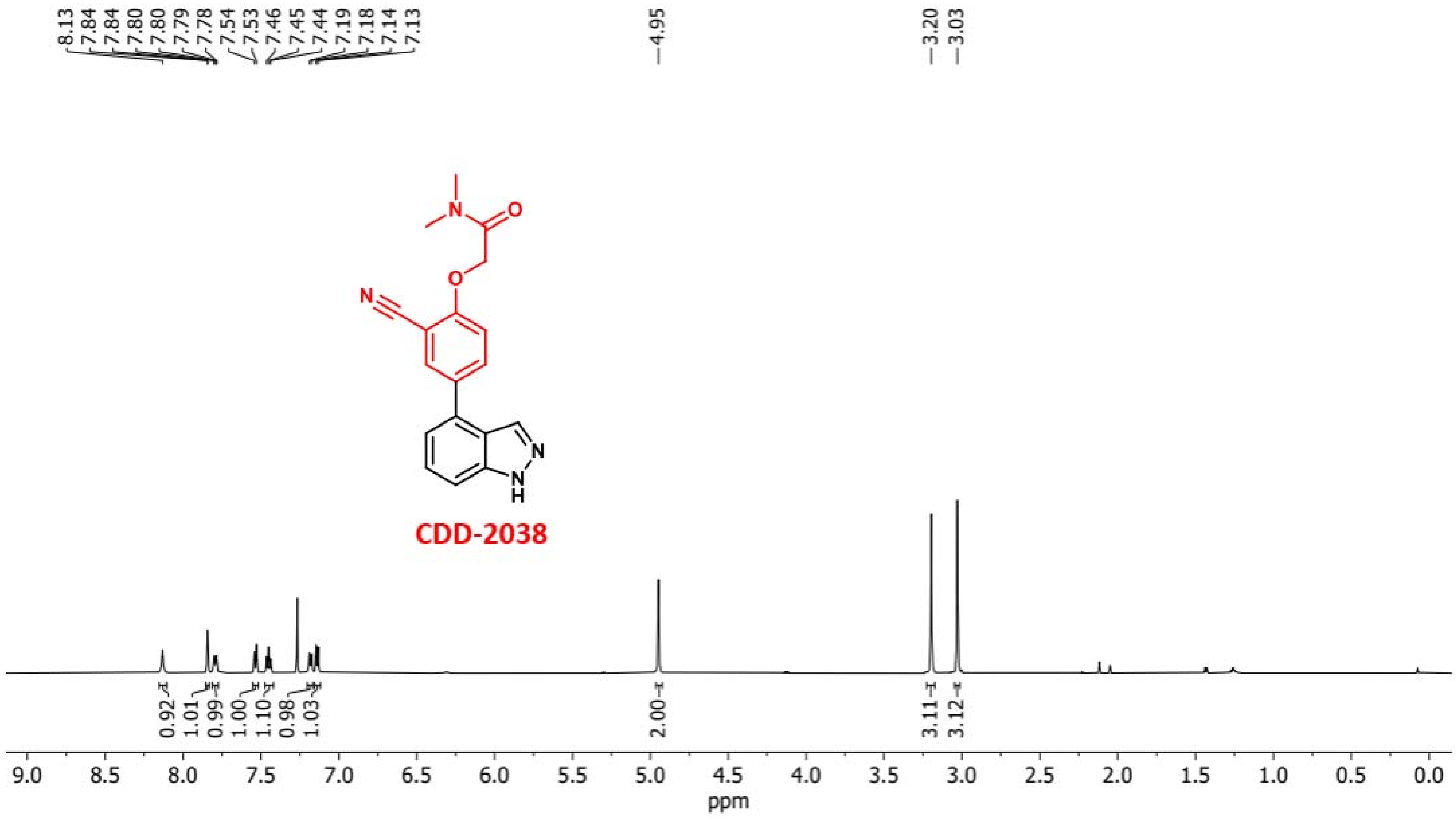

8.13
7.84
7.84
7.80
7.80
7.79
7.78
7.54
7.53
7.46
7.45
7.44
7.19
7.18
7.14
7.13
4.95
3.20
3.03
N
O
O
N
N
N
H
CDD-2038
0.92
1.01
0.99
1.00
1.10
0.98
1.03
2.00
3.11
3.12
9.0
8.5
8.0
7.5
7.0
6.5
6.0
5.5
5.0
4.5
4.0
3.5
3.0
2.5
2.0
1.5
1.0
0.5
0.0
ppm

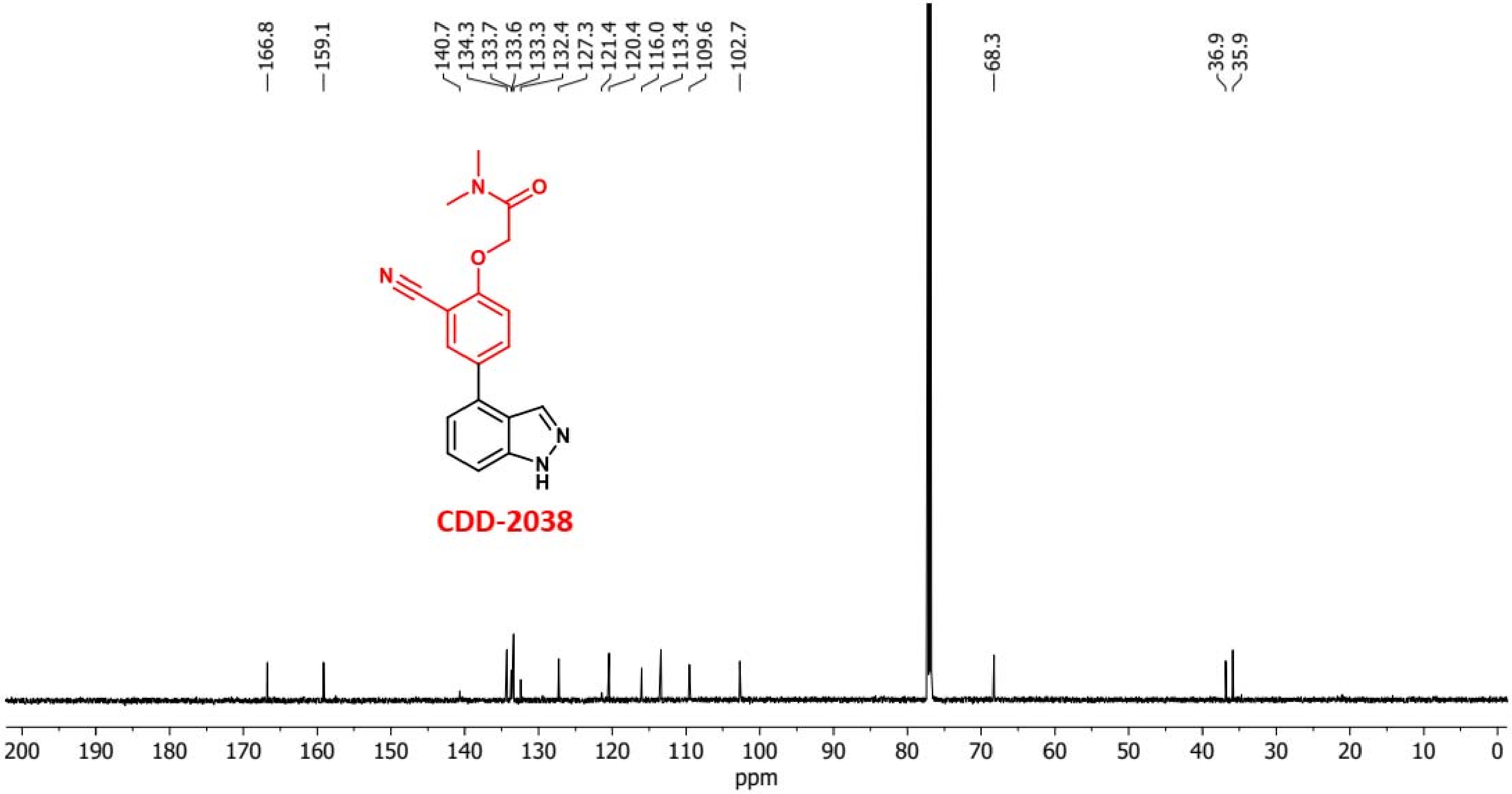
166.8
159.1
140.7
134.3
133.7
133.6
133.3
132.4
127.3
121.4
120.4
116.0
113.4
109.6
102.7
68.3
36.9
35.9
CDD-2038
200 190 180 170 160 150 140 130 120 110 100 90 80 70 60 50 40 30 20 10 0
ppm

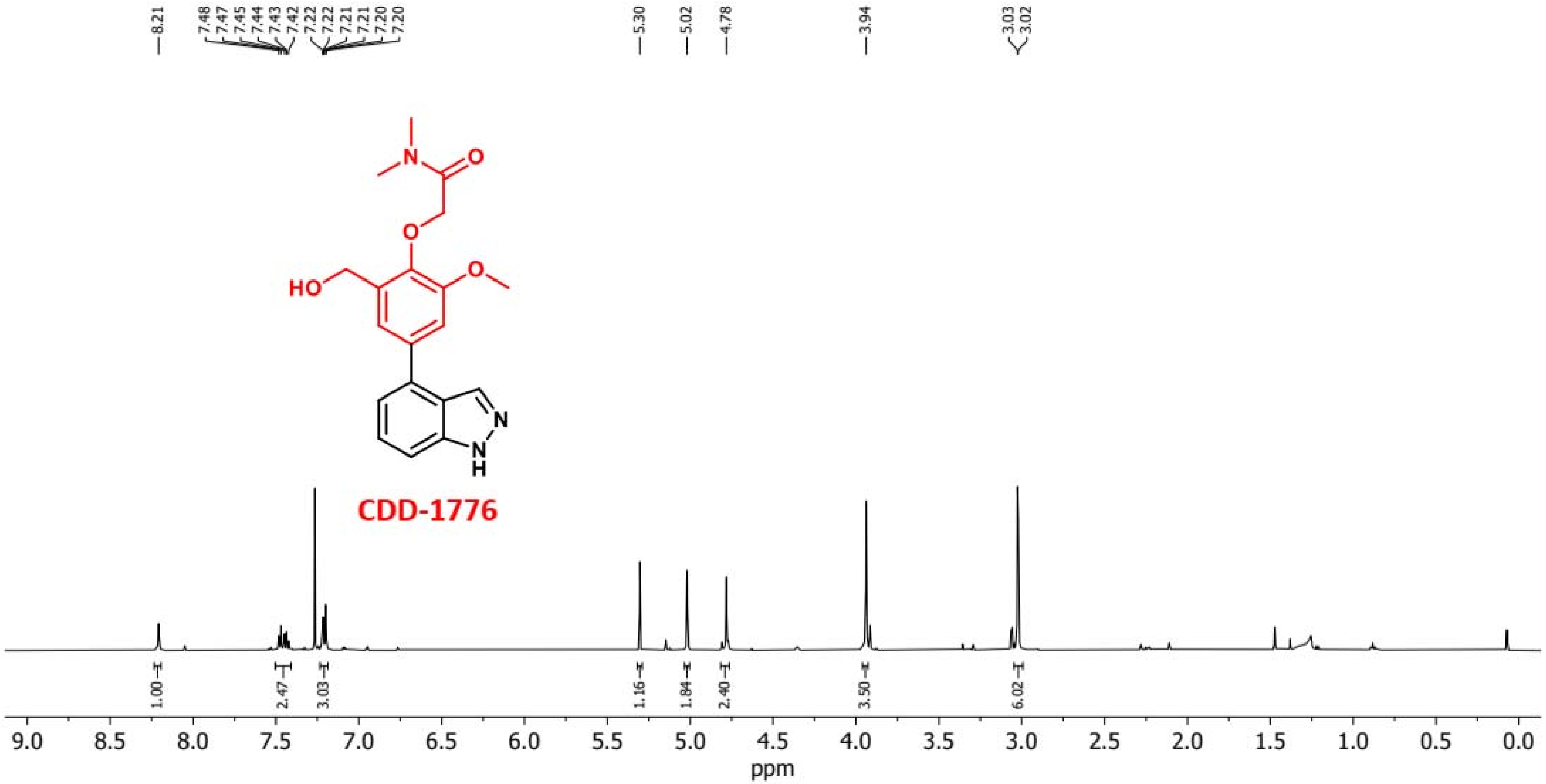
8.21
7.48
7.47
7.45
7.44
7.43
7.42
7.22
7.22
7.22
7.21
7.21
7.20
7.20
5.30
5.02
4.78
3.94
3.03
3.02
HO
O
N
H
1.00
2.47
3.03
1.16
1.84
2.40
3.50
6.02
9.0
8.5
8.0
7.5
7.0
6.5
6.0
5.5
5.0
4.5
4.0
3.5
3.0
2.5
2.0
1.5
1.0
0.5
0.0
ppm

CDD-1776

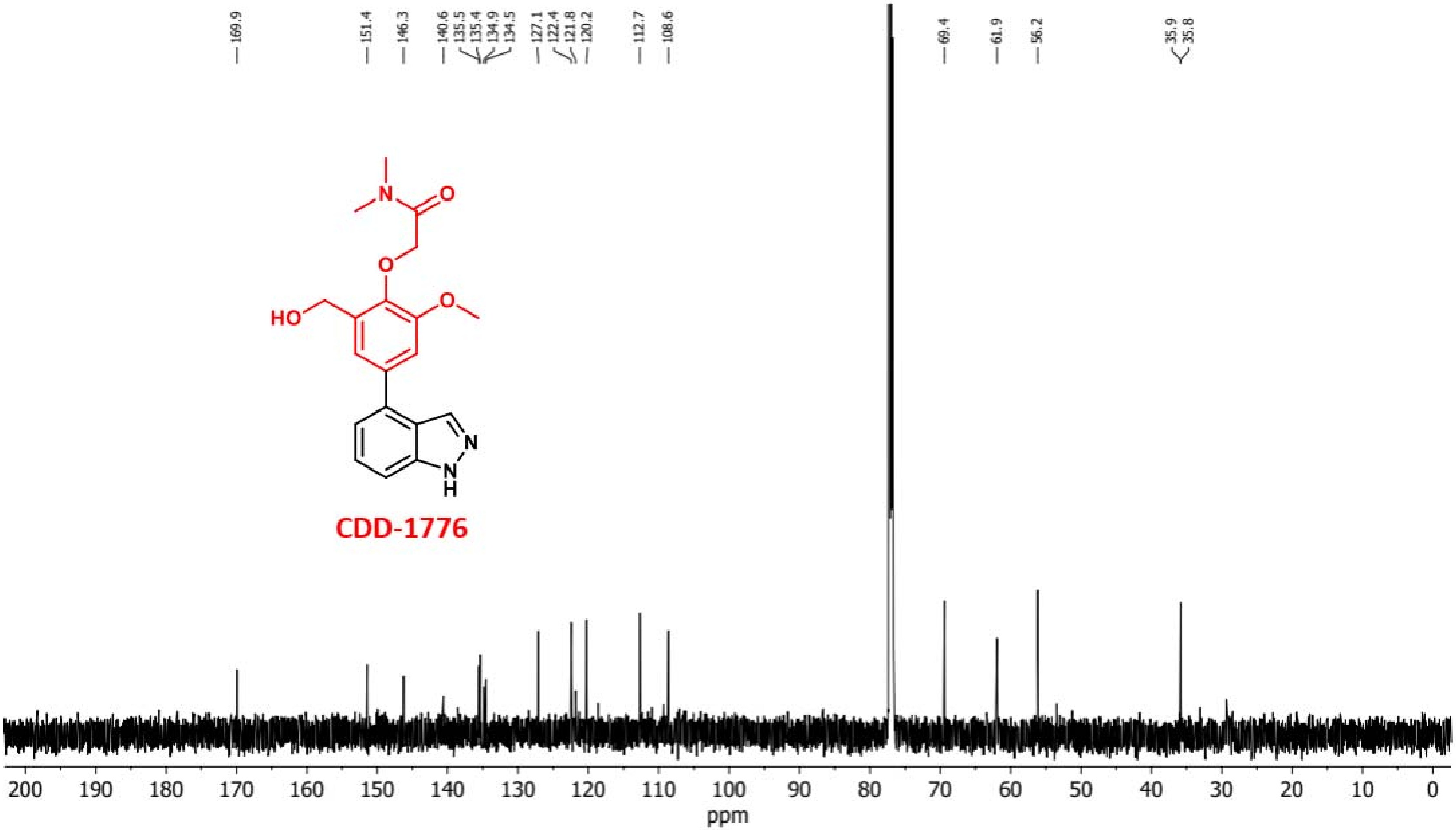
169.9
151.4
146.3
140.6
135.5
135.4
134.9
134.5
127.1
122.4
121.8
120.2
112.7
108.6
69.4
61.9
56.2
35.9
35.8
HO
O
N
H
CDD-1776
200
190
180
170
160
150
140
130
120
110
100
90
80
70
60
50
40
30
20
10
0
ppm

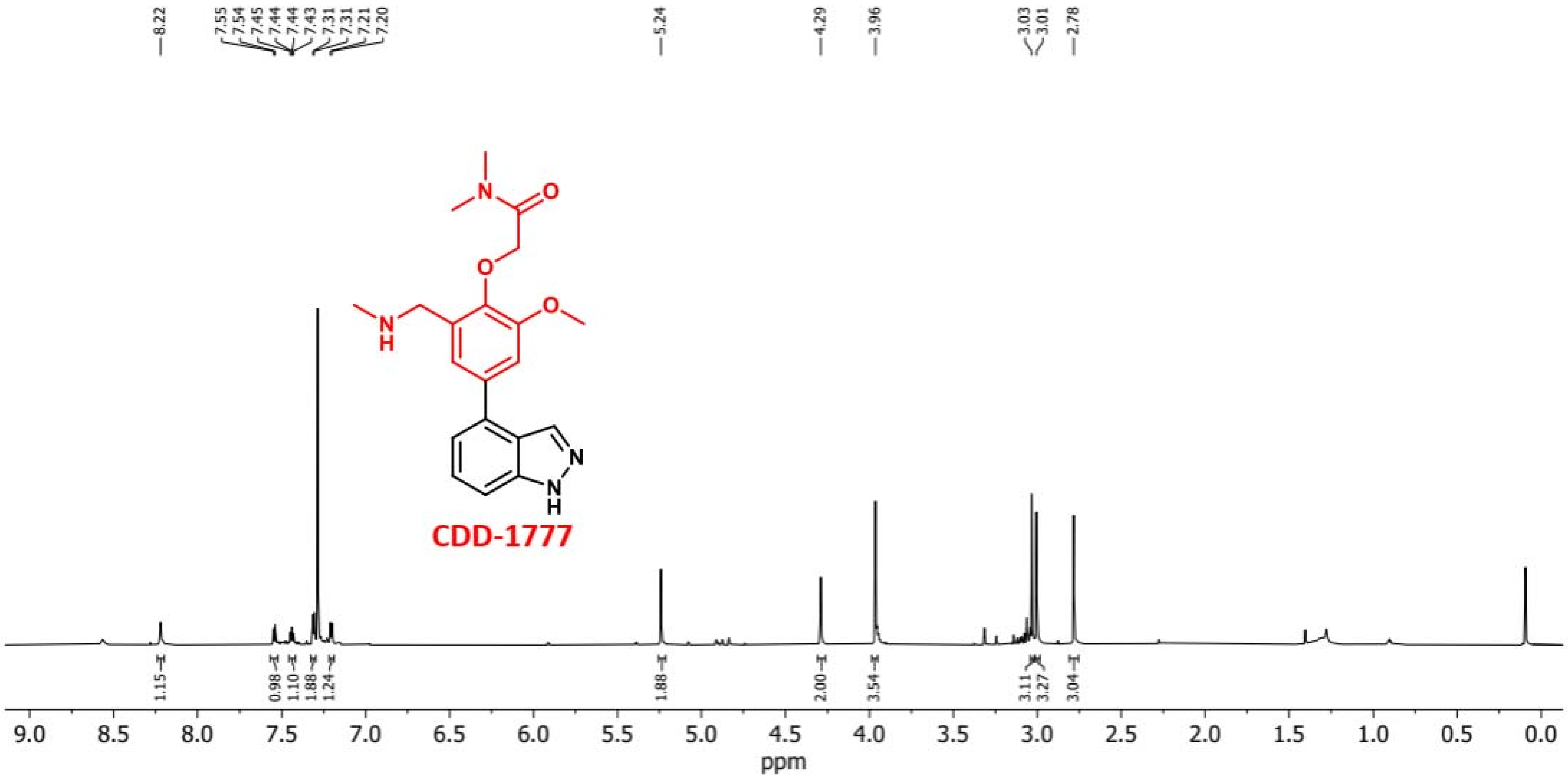
8.22
7.55
7.54
7.45
7.44
7.44
7.43
7.31
7.31
7.21
7.20
5.24
4.29
3.96
3.03
3.01
2.78
CDD-1777
1.15
0.98
1.10
1.88
1.24
1.88
2.00
3.54
3.11
3.27
3.04
9.0 8.5 8.0 7.5 7.0 6.5 6.0 5.5 5.0 4.5 4.0 3.5 3.0 2.5 2.0 1.5 1.0 0.5 0.0
ppm

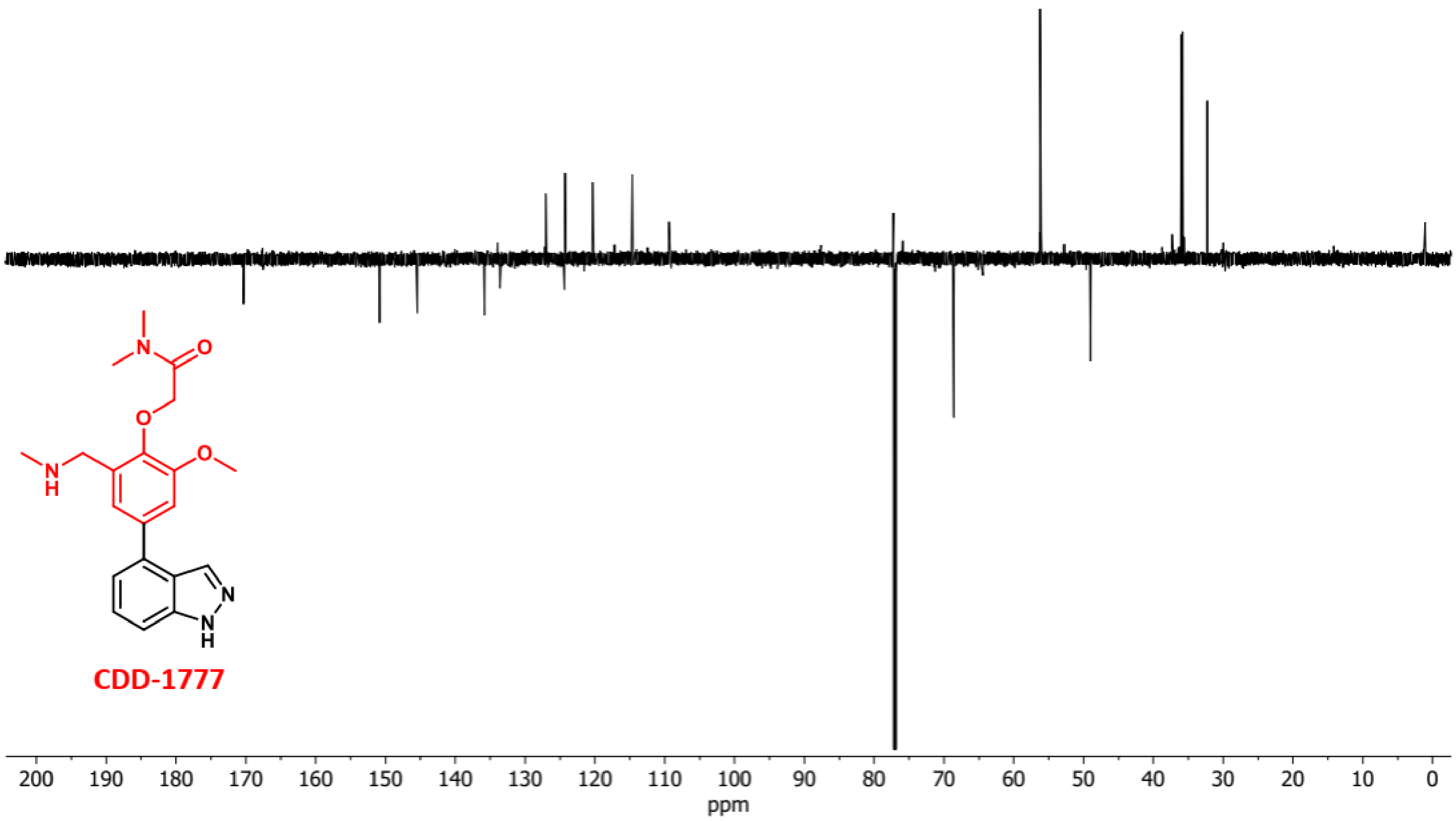

N
O
O
O
N
H
N
N
H
CDD-1777
200 190 180 170 160 150 140 130 120 110 100 90 80 70 60 50 40 30 20 10 0
ppm

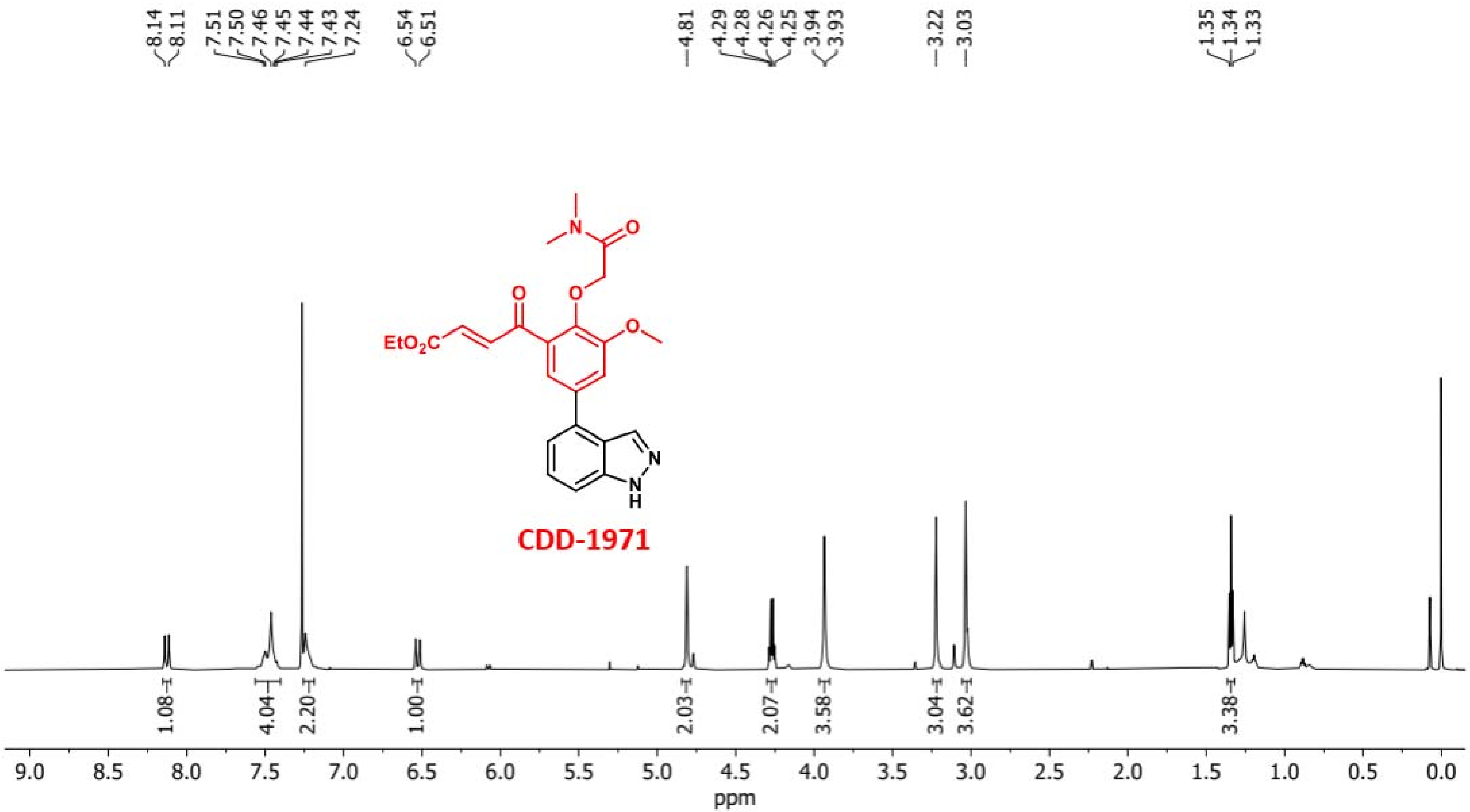

8.14
8.11
7.51
7.50
7.46
7.45
7.44
7.43
7.24
6.54
6.51
4.81
4.29
4.28
4.26
4.25
3.94
3.93
3.22
3.03
1.35
1.34
1.33
EtO2C
O
N
H
CDD-1971
1.08
4.04
2.20
1.00
2.03
2.07
3.58
3.04
3.62
3.38
9.0
8.5
8.0
7.5
7.0
6.5
6.0
5.5
5.0
4.5
4.0
3.5
3.0
2.5
2.0
1.5
1.0
0.5
0.0
ppm

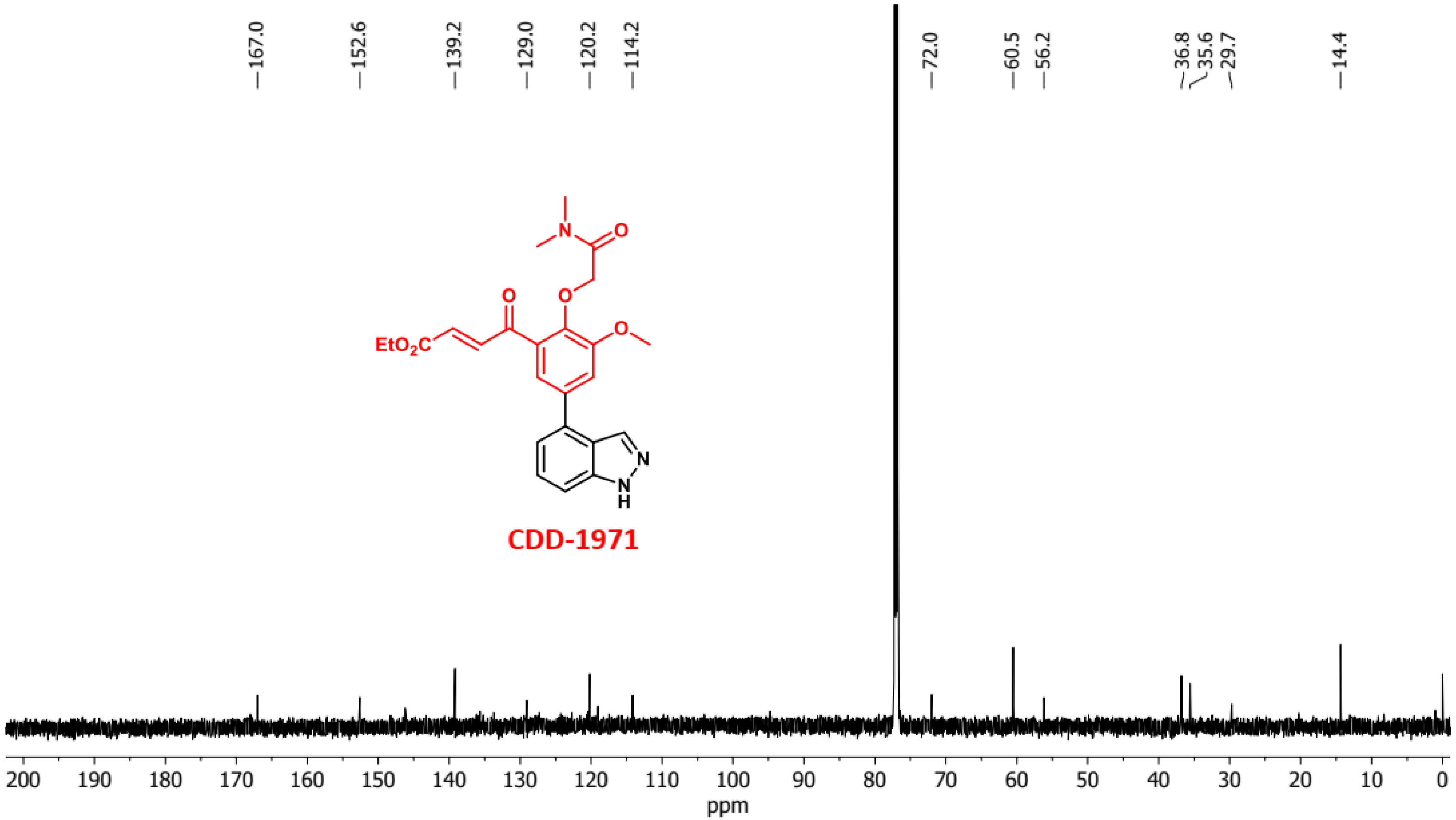
167.0
152.6
139.2
129.0
120.2
114.2
72.0
60.5
56.2
36.8
35.6
29.7
14.4
EtO2C
O
N
H
CDD-1971
200 190 180 170 160 150 140 130 120 110 100 90 80 70 60 50 40 30 20 10 0
ppm

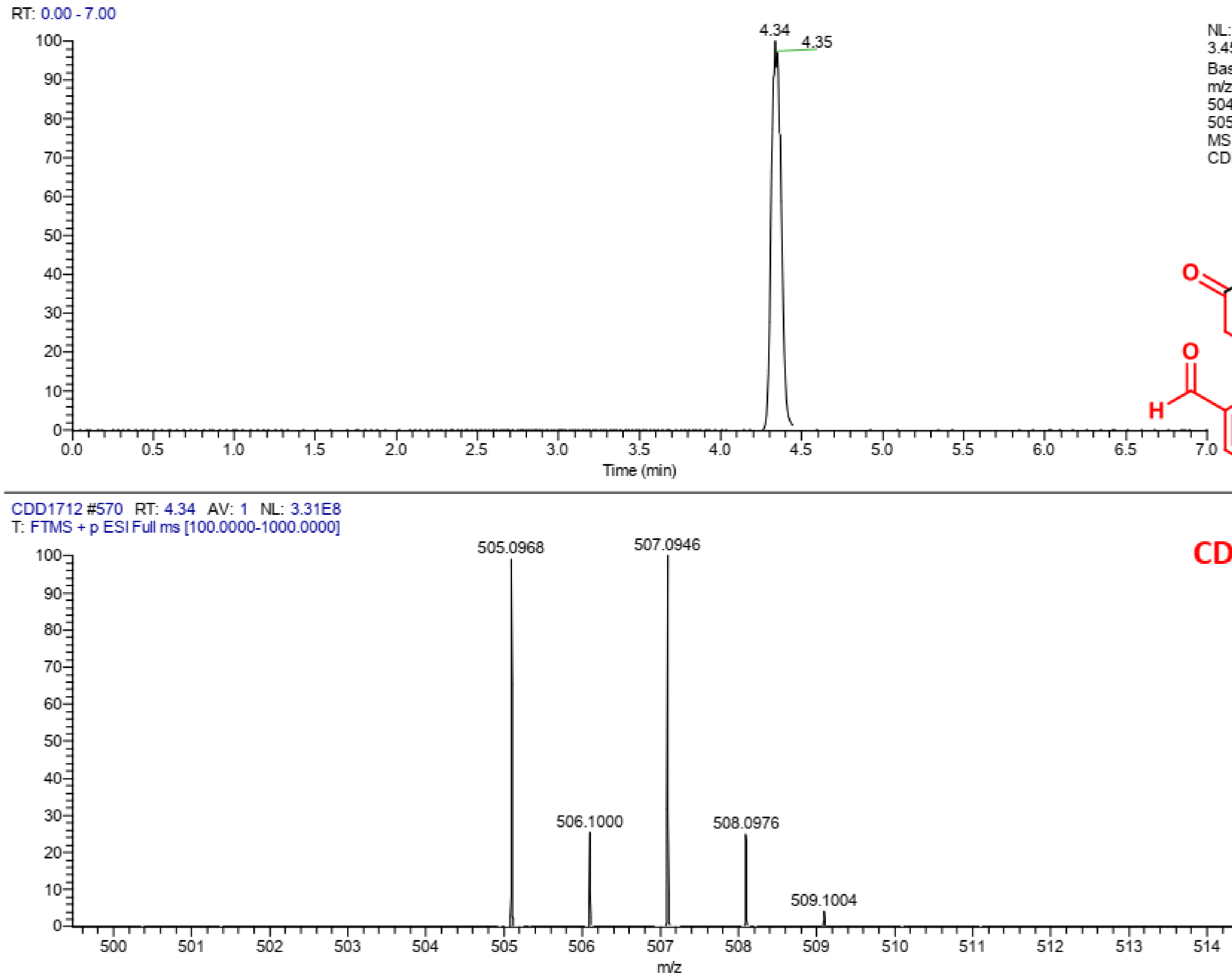
RT: 0.00 - 7.00
4.34
4.35
NL:
3.45E8
Base Peak
m/z=
504.5969-
505.5969
MS
CDD1712
Time (min)
CDD1712 #570 RT: 4.34 AV: 1 NL: 3.31E8
T: FTMS + p ESI Full ms [100.0000-1000.0000]
505.0968
507.0946
506.1000
508.0976
509.1004
m/z

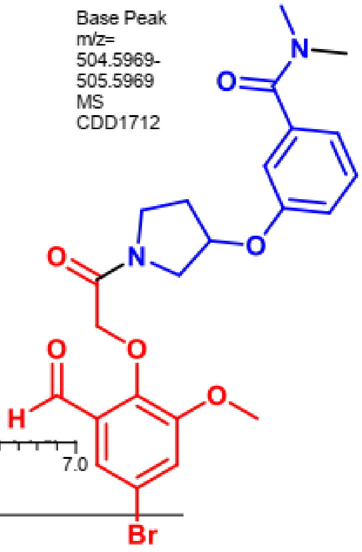
N
O
O
N
O
O
O
H
O
Br

CDD-1712

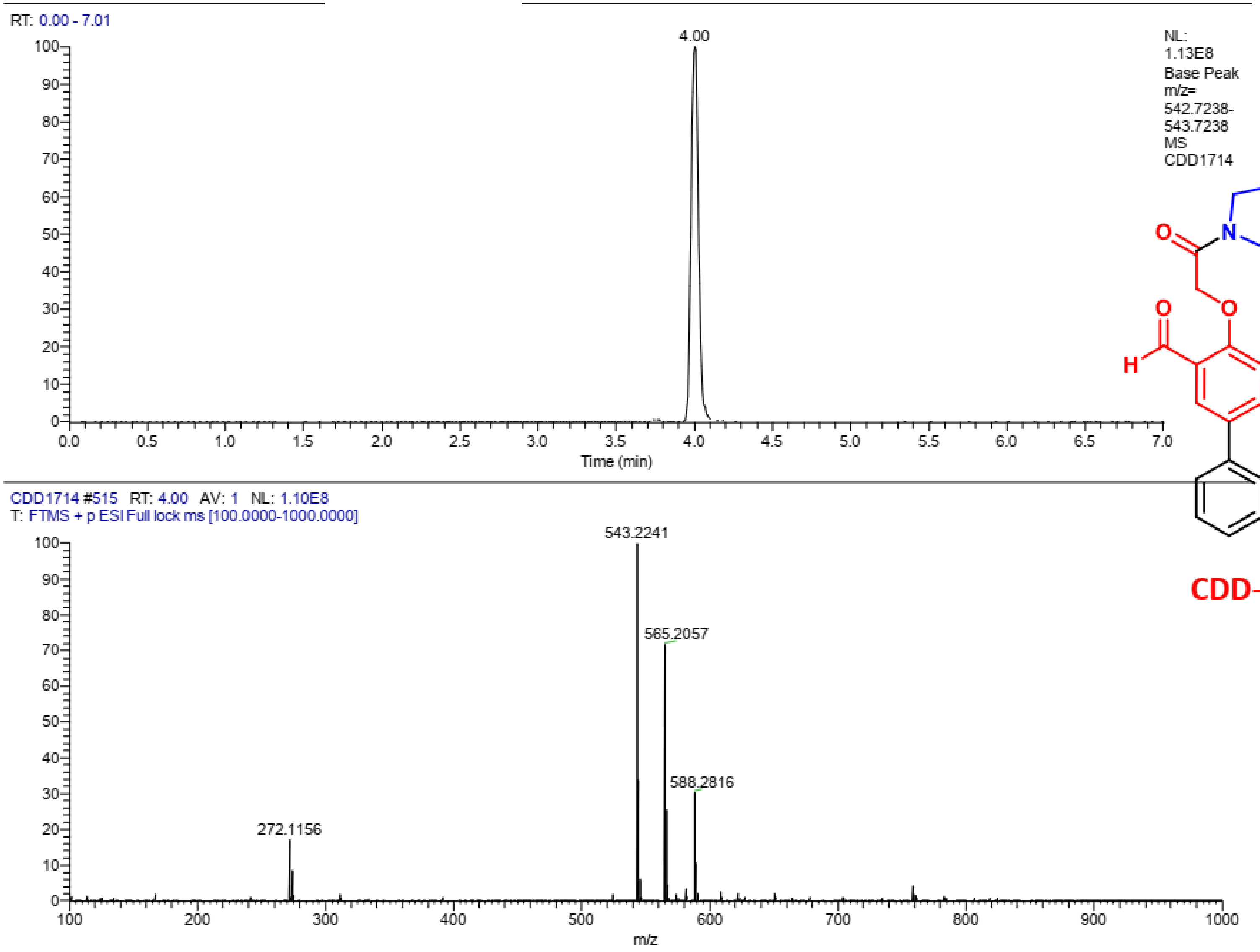
RT: 0.00 - 7.01
4.00
NL:
1.13E8
Base Peak
m/z=
542.7238-
543.7238
MS
CDD1714
Time (min)
CDD1714 #515 RT: 4.00 AV: 1 NL: 1.10E8
T: FTMS + p ESI Full lock ms [100.0000-1000.0000]
543.2241
565.2057
588.2816
272.1156
m/z

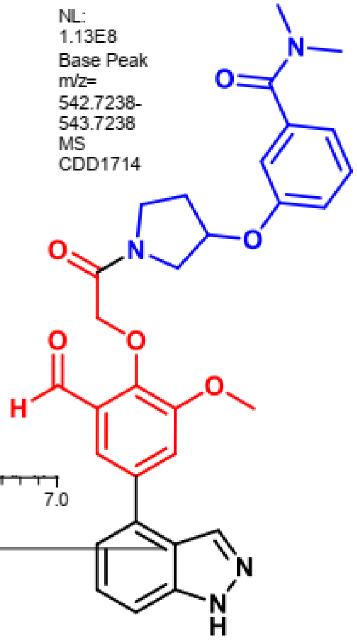

CDD-1714

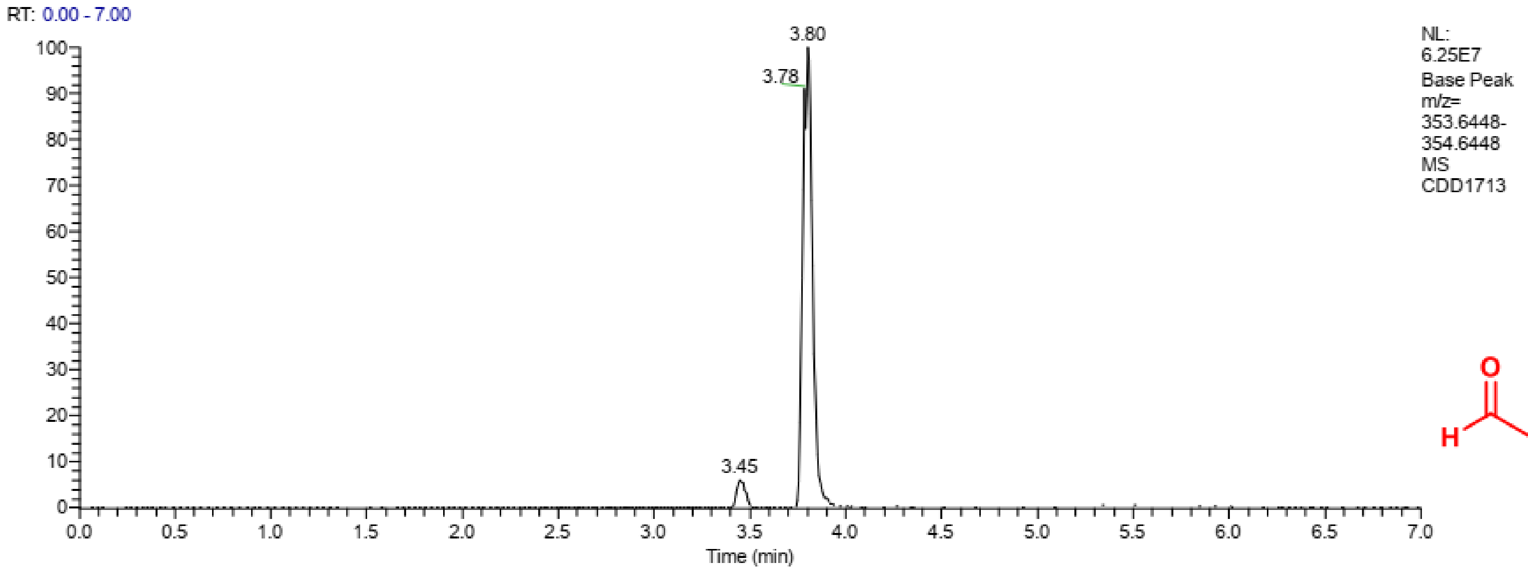
RT: 0.00 - 7.00
3.80
3.78
3.45
NL:
6.25E7
Base Peak
m/z=
353.6448-
354.6448
MS
CDD1713
Time (min)

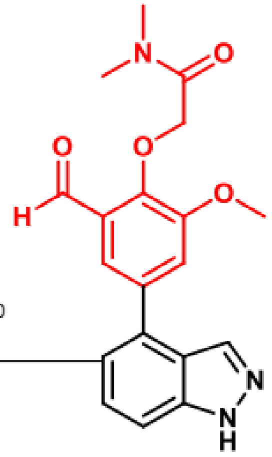
O
N
O
O
O
H
N
N
H

CDD-1713

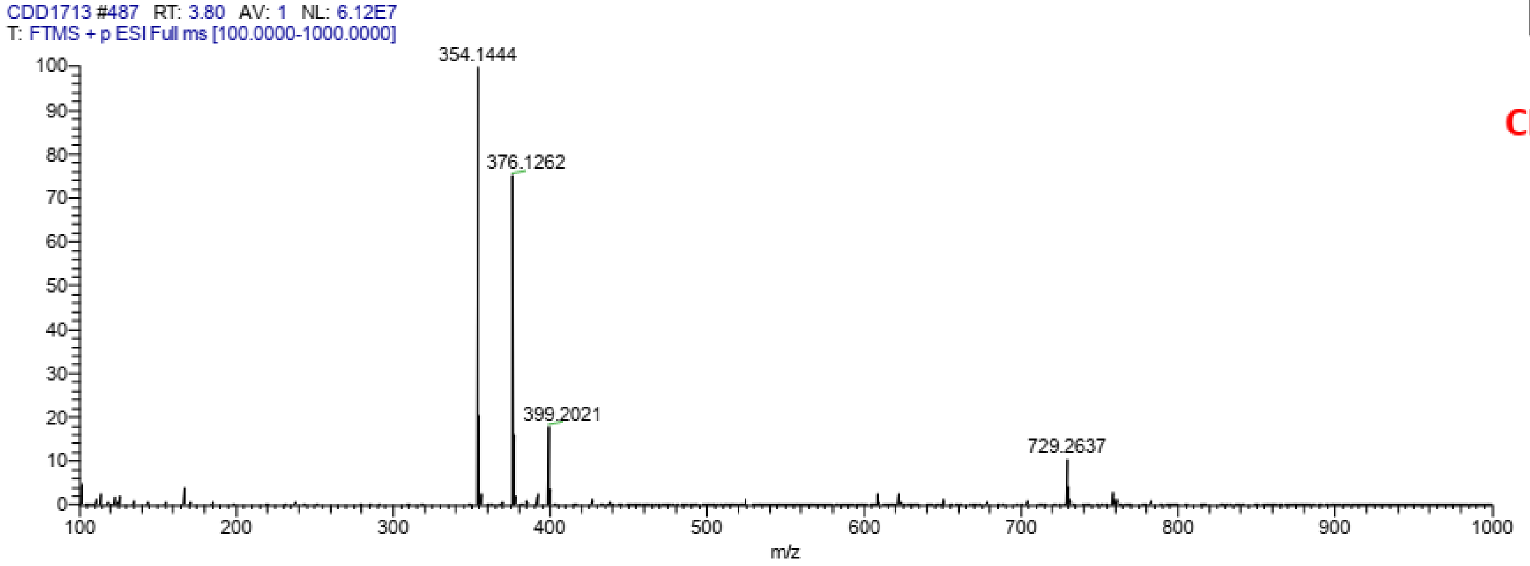
CDD1713 #487 RT: 3.80 AV: 1 NL: 6.12E7
T: FTMS + p ESI Full ms [100.0000-1000.0000]
354.1444
376.1262
399.2021
729.2637
m/z

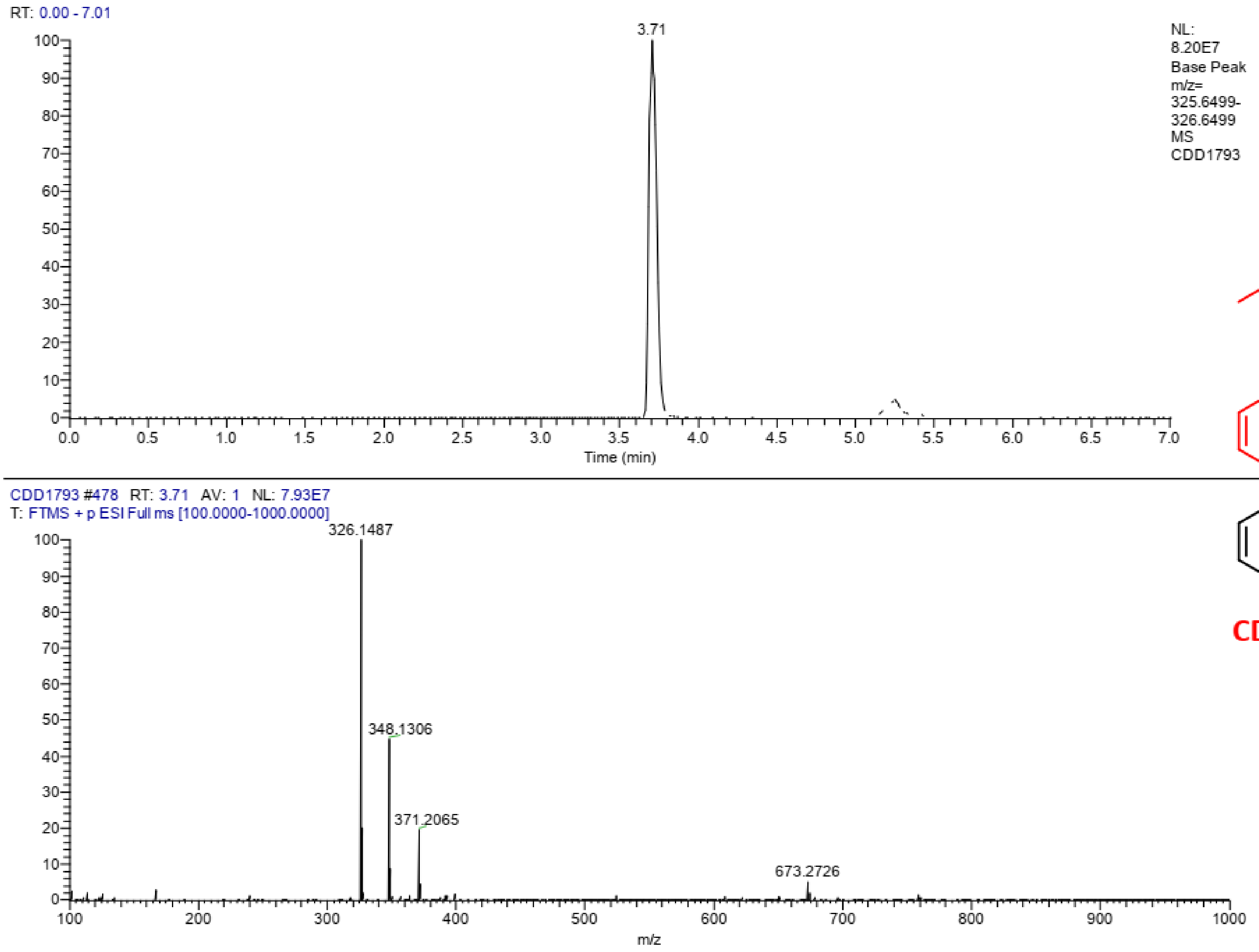
RT: 0.00 - 7.01
3.71
NL:
8.20E7
Base Peak
m/z=
325.6499-
326.6499
MS
CDD1793
Time (min)
CDD1793 #478 RT: 3.71 AV: 1 NL: 7.93E7
T: FTMS + p ESI Full ms [100.0000-1000.0000]
326.1487
348.1306
371.2065
673.2726
m/z

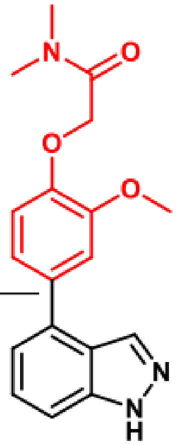

CDD-1793

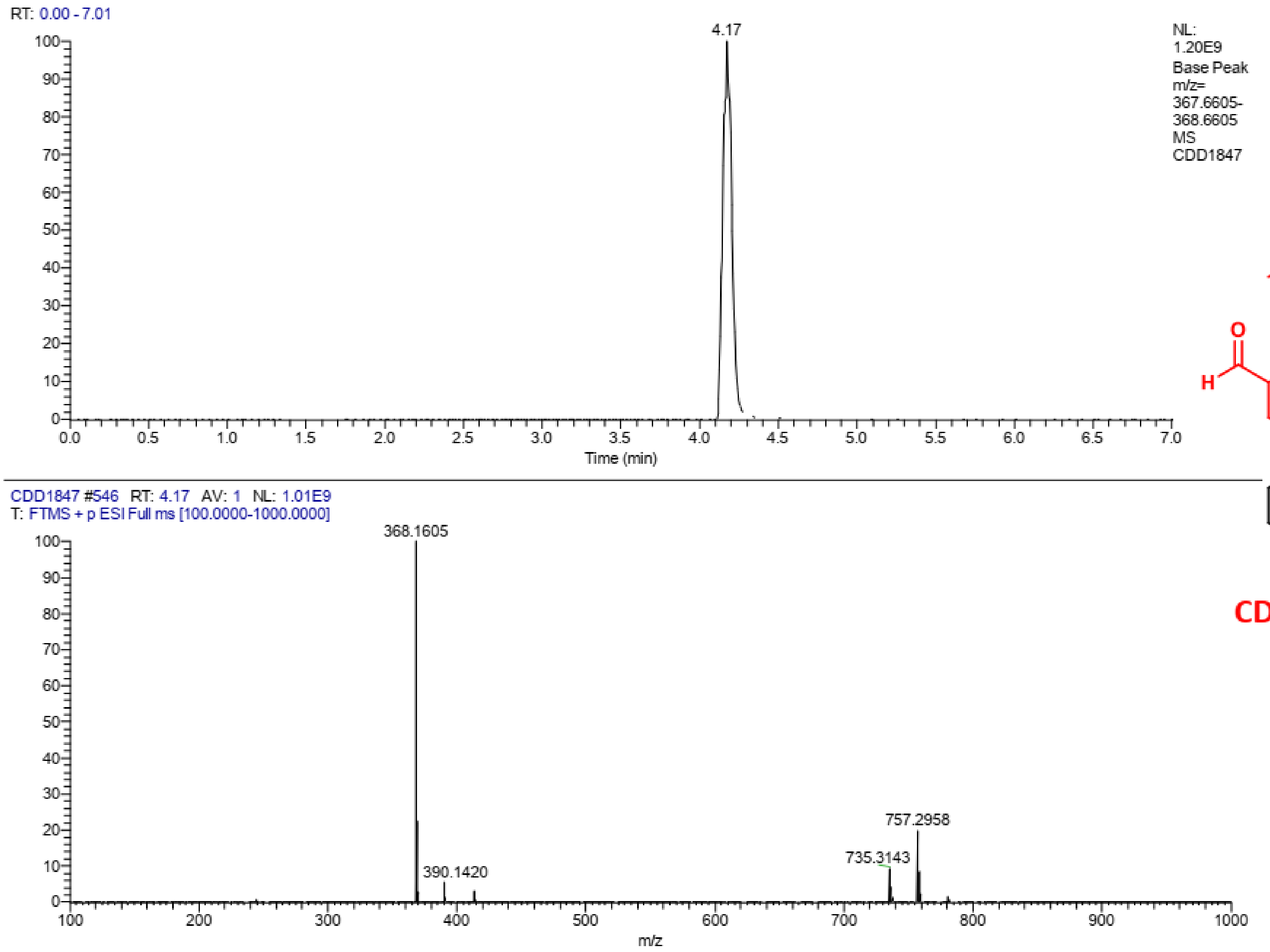
RT: 0.00 - 7.01
4.17
NL:
1.20E9
Base Peak
m/z=
367.6605-
368.6605
MS
CDD1847
Time (min)
CDD1847 #546 RT: 4.17 AV: 1 NL: 1.01E9
T: FTMS + p ESI Full ms [100.0000-1000.0000]
368.1605
390.1420
735.3143
757.2958
m/z

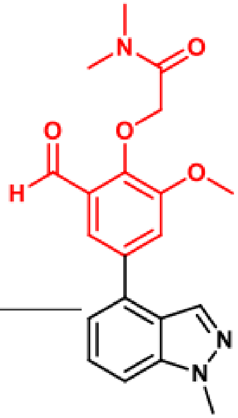

CDD-1847

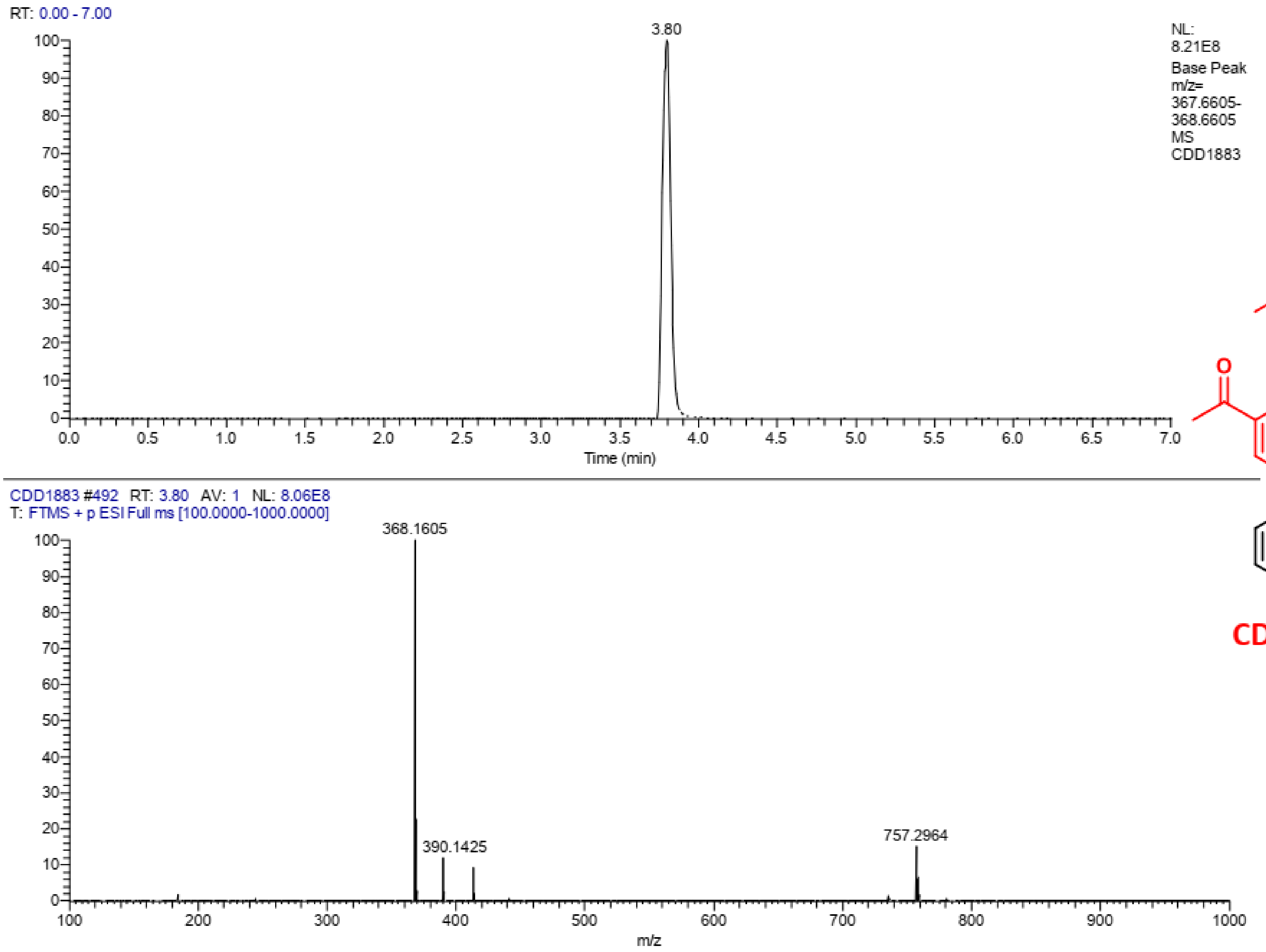
RT: 0.00 - 7.00
3.80
NL:
8.21E8
Base Peak
m/z=
367.6605-
368.6605
MS
CDD1883
Time (min)
CDD1883 #492 RT: 3.80 AV: 1 NL: 8.06E8
T: FTMS + p ESI Full ms [100.0000-1000.0000]
368.1605
390.1425
757.2964
m/z

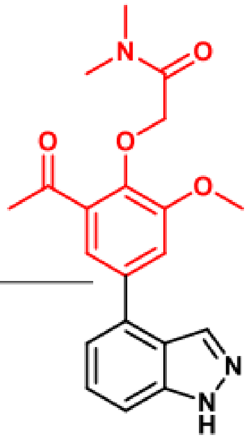
N
O
O
O
O
N
N
H

CDD-1883

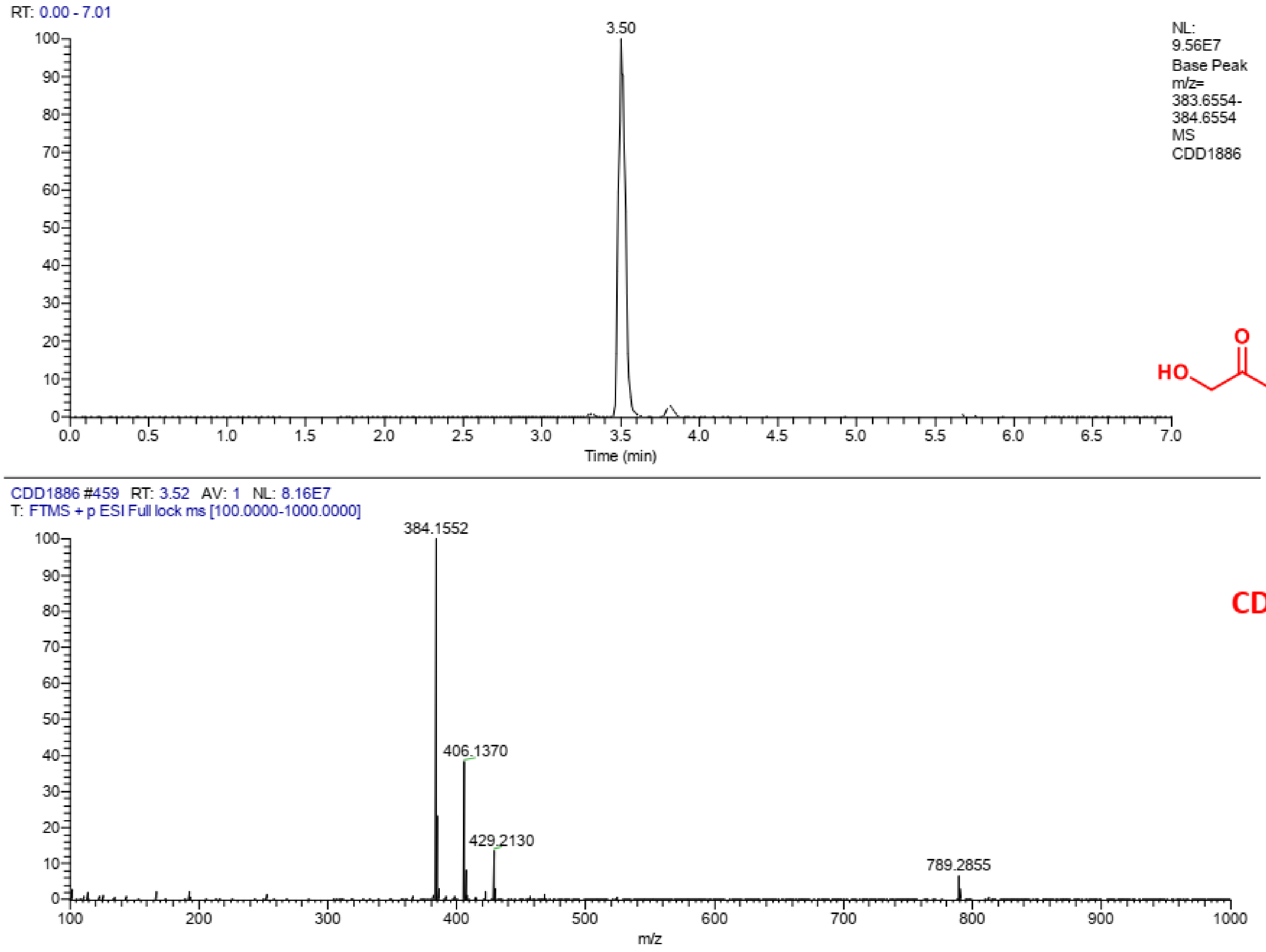
RT: 0.00 - 7.01
3.50
NL:
9.56E7
Base Peak
m/z=
383.6554-
384.6554
MS
CDD1886
Time (min)
CDD1886 #459 RT: 3.52 AV: 1 NL: 8.16E7
T: FTMS + p ESI Full lock ms [100.0000-1000.0000]
384.1552
406.1370
429.2130
789.2855
m/z

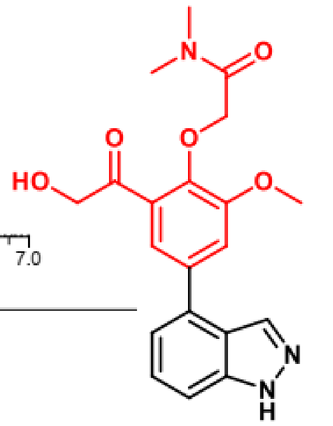
HO
O
N
H

CDD-1886

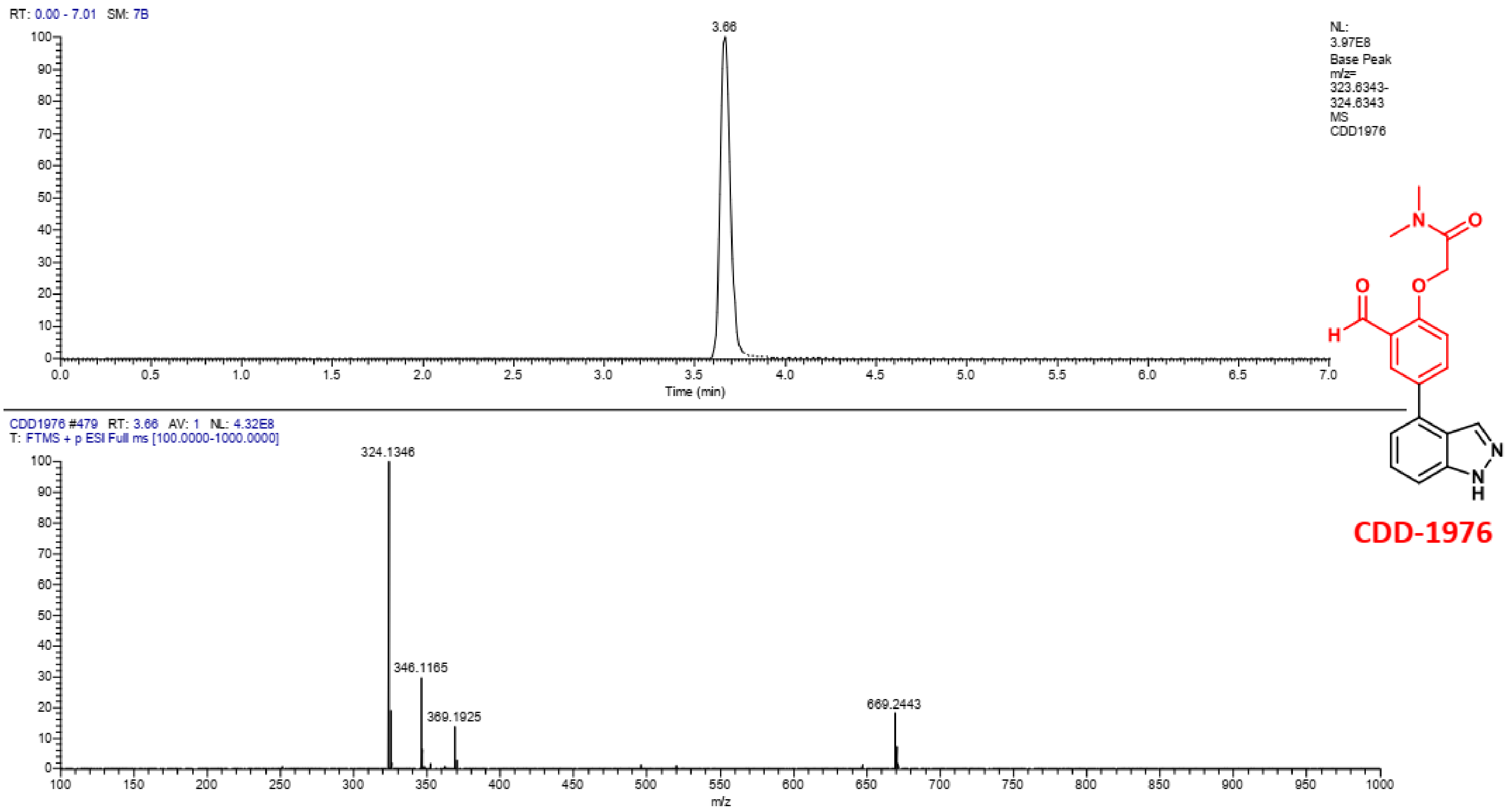
RT: 0.00 - 7.01 SM: 7B
3.66
NL:
3.97E8
Base Peak
m/z=
323.6343-
324.6343
MS
CDD1976
Time (min)
CDD1976 #479 RT: 3.66 AV: 1 NL: 4.32E8
T: FTMS + p ESI Full ms [100.0000-1000.0000]
324.1346
346.1165
369.1925
669.2443
m/z
O
N
H
N
H
CDD-1976

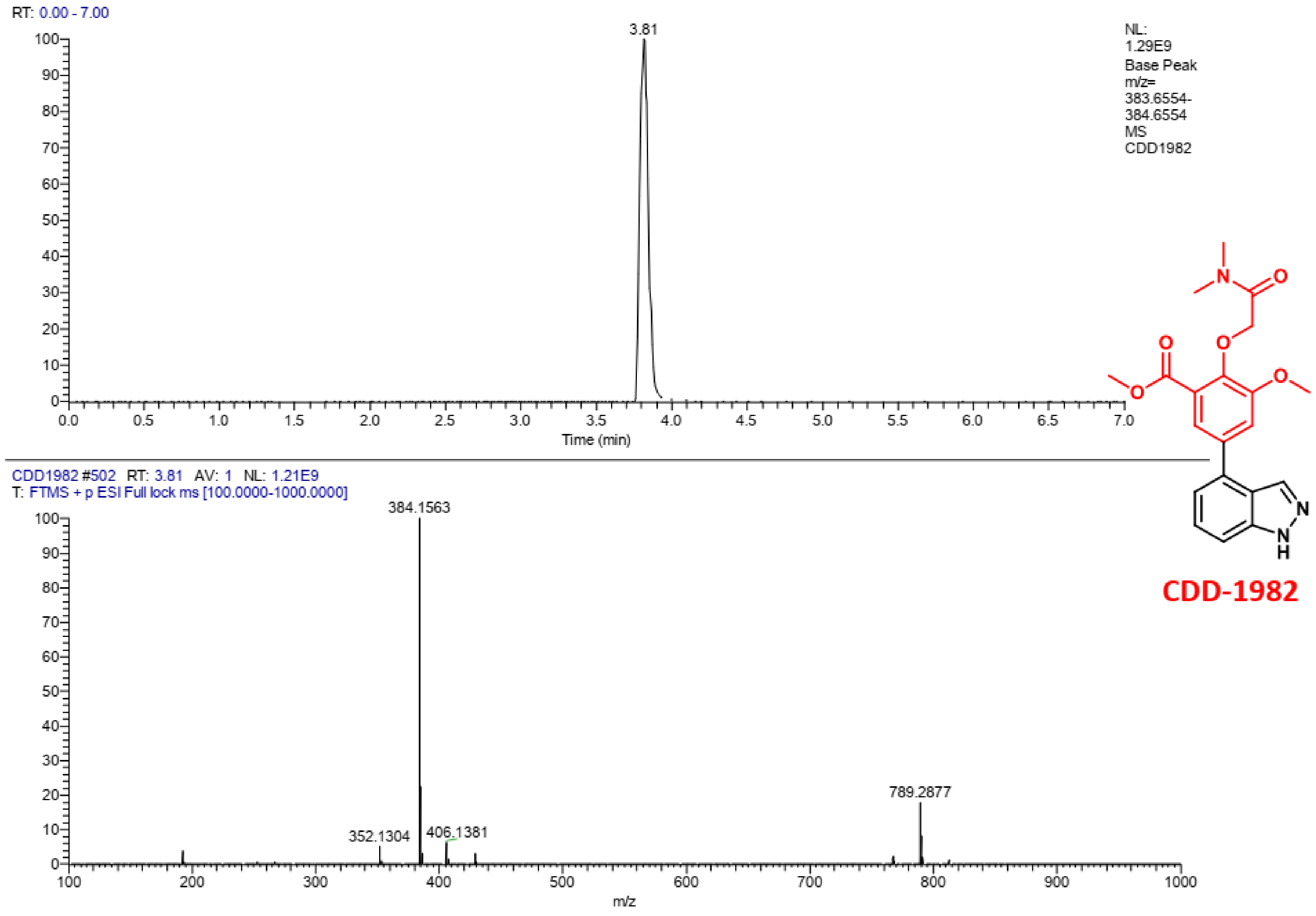
RT: 0.00 - 7.00
3.81
NL: 1.29E9 Base Peak m/z= 383.6554-384.6554 MS CDD1982
Time (min)
CDD1982 #502 RT: 3.81 AV: 1 NL: 1.21E9
T: FTMS + p ESI Full lock ms [100.0000-1000.0000]
384.1563
352.1304
406.1381
789.2877
m/z
O
N
H
CDD-1982

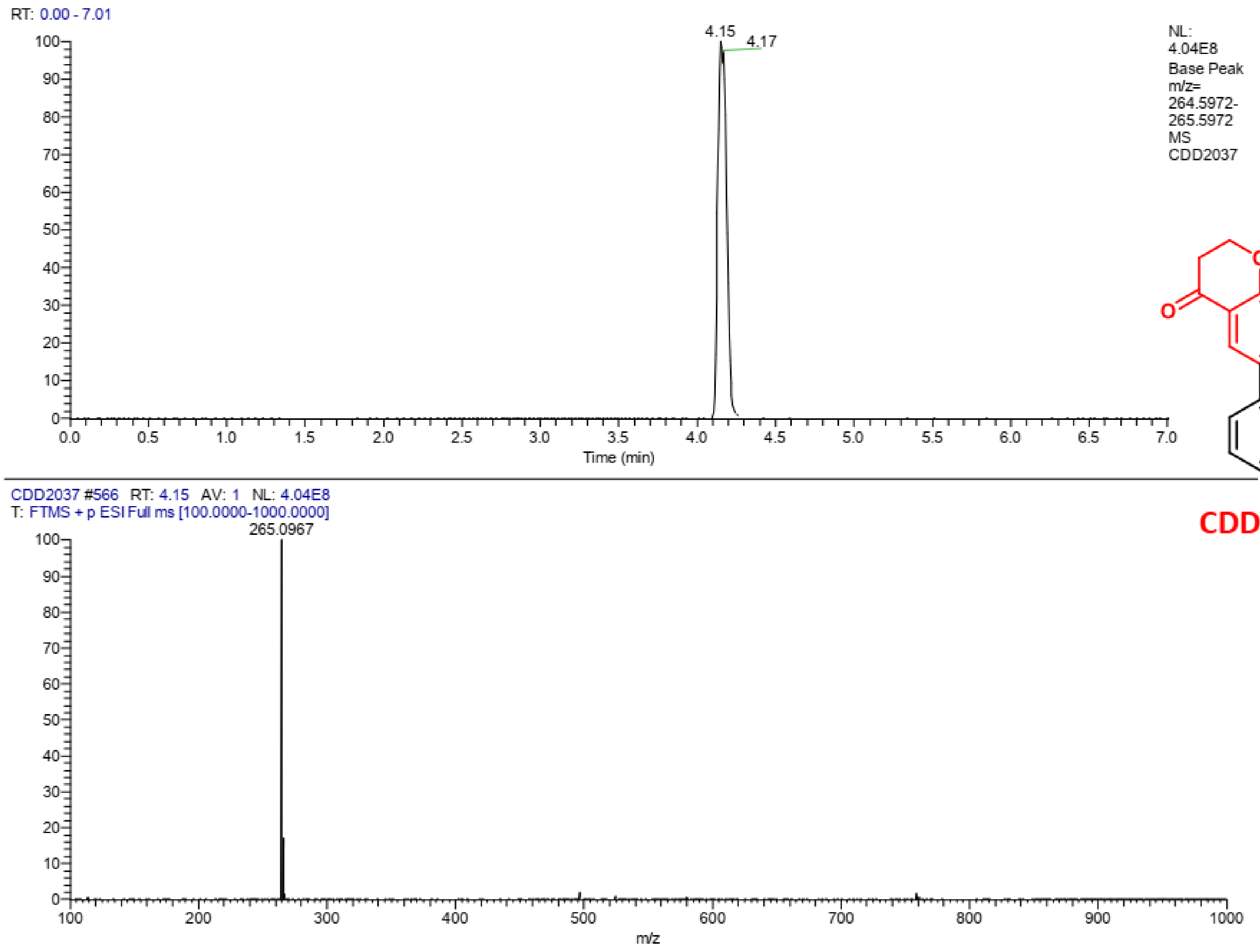
RT: 0.00 - 7.01
4.15
4.17
NL:
4.04E8
Base Peak
m/z=
264.5972-
265.5972
MS
CDD2037
Time (min)
CDD2037 #566 RT: 4.15 AV: 1 NL: 4.04E8
T: FTMS + p ESI Full ms [100.0000-1000.0000]
265.0967
m/z

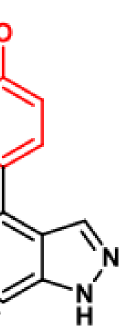

CDD-2037

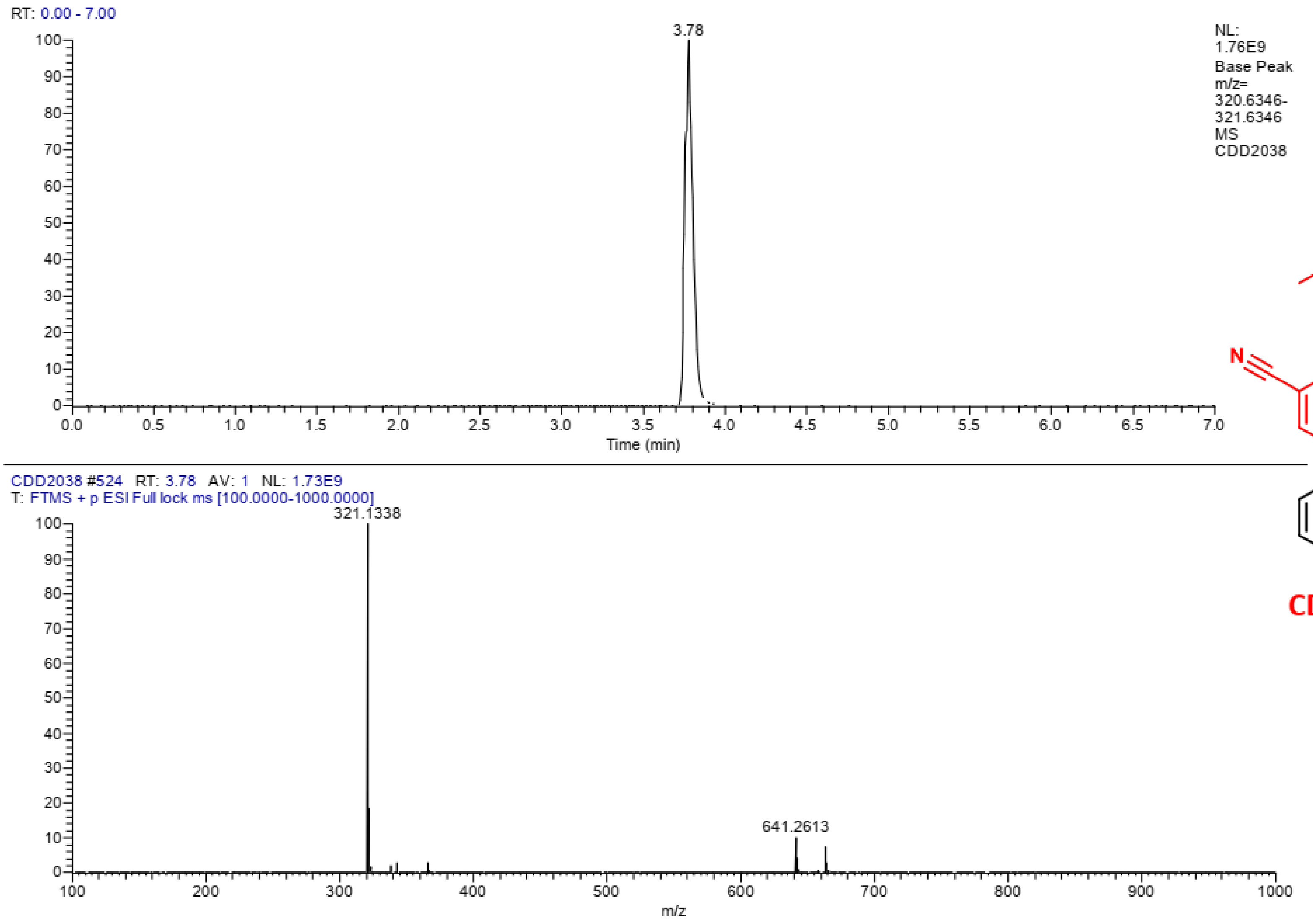
RT: 0.00 - 7.00
3.78
NL:
1.76E9
Base Peak
m/z=
320.6346-
321.6346
MS
CDD2038
Time (min)
CDD2038 #524 RT: 3.78 AV: 1 NL: 1.73E9
T: FTMS + p ESI Full lock ms [100.0000-1000.0000]
321.1338
641.2613
m/z

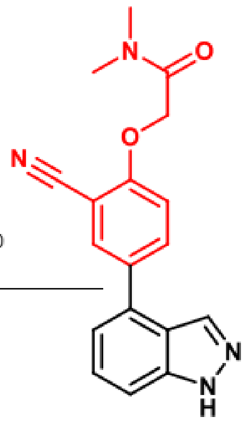

CDD-2038

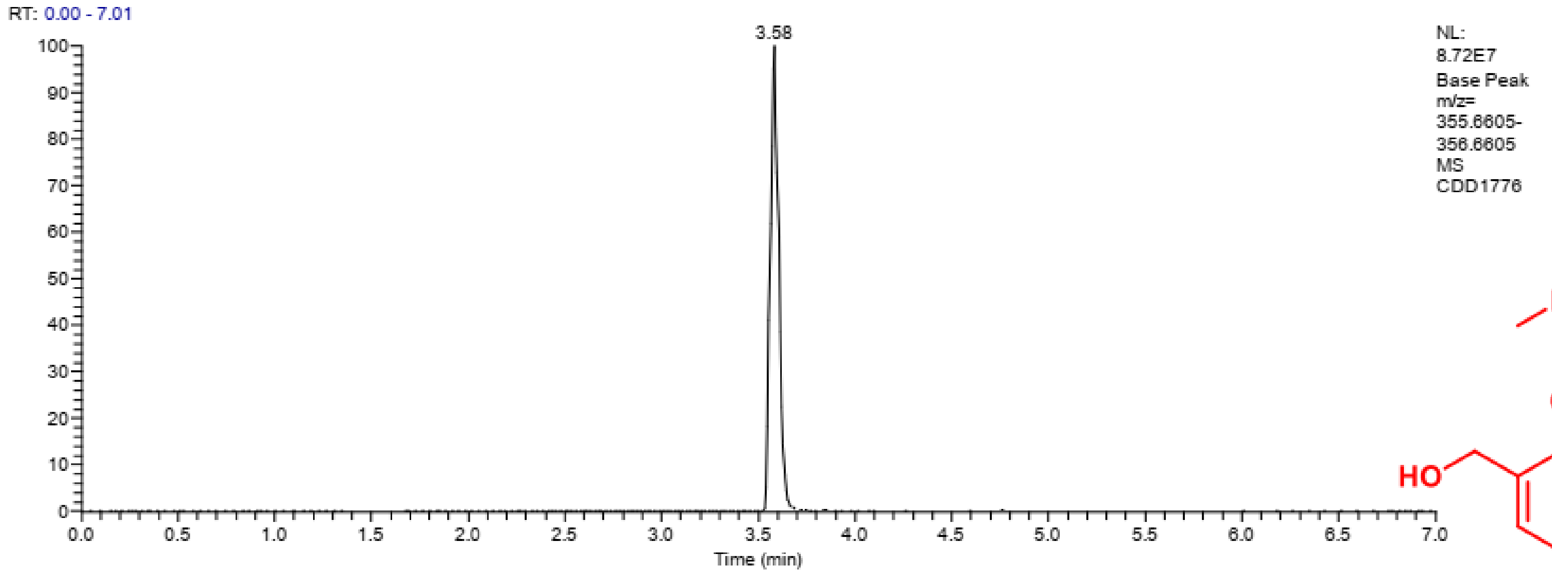
RT: 0.00 - 7.01
3.58
NL:
8.72E7
Base Peak
m/z=
355.6605-
356.6605
MS
CDD1776
Time (min)

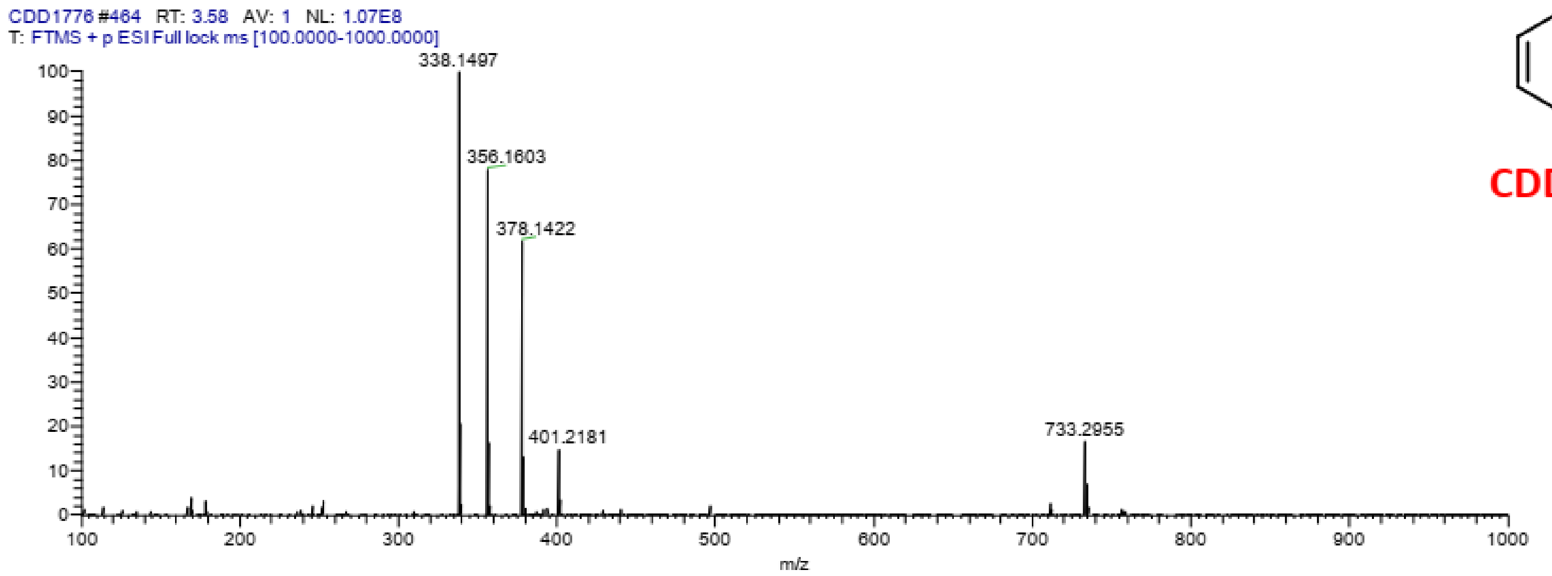
CDD1776 #464 RT: 3.58 AV: 1 NL: 1.07E8
T: FTMS + p ESI Full lock ms [100.0000-1000.0000]
338.1497
356.1603
378.1422
401.2181
733.2955
m/z

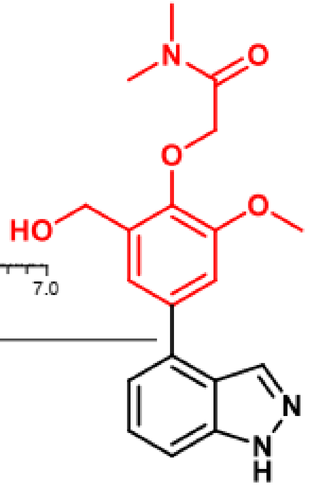
HO
O
O
N
O
N
N
H

CDD-1776

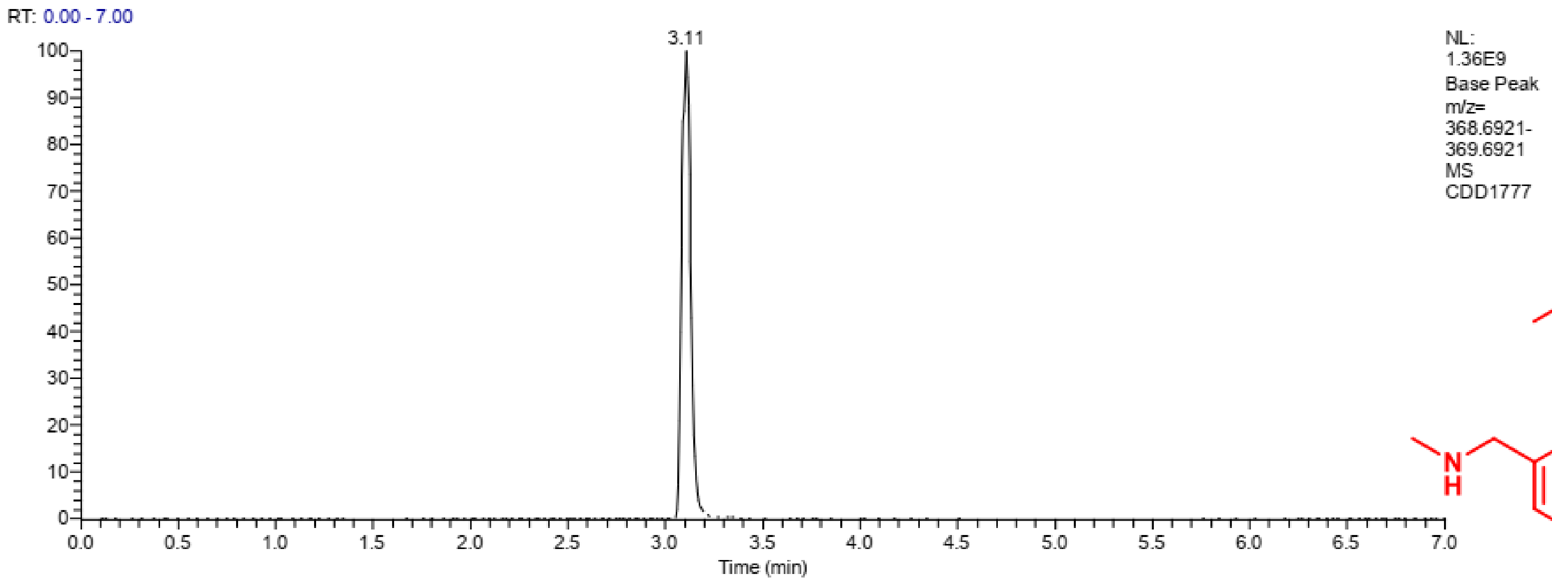
RT: 0.00 - 7.00
3.11
NL:
1.36E9
Base Peak
m/z=
368.6921-
369.6921
MS
CDD1777
Time (min)

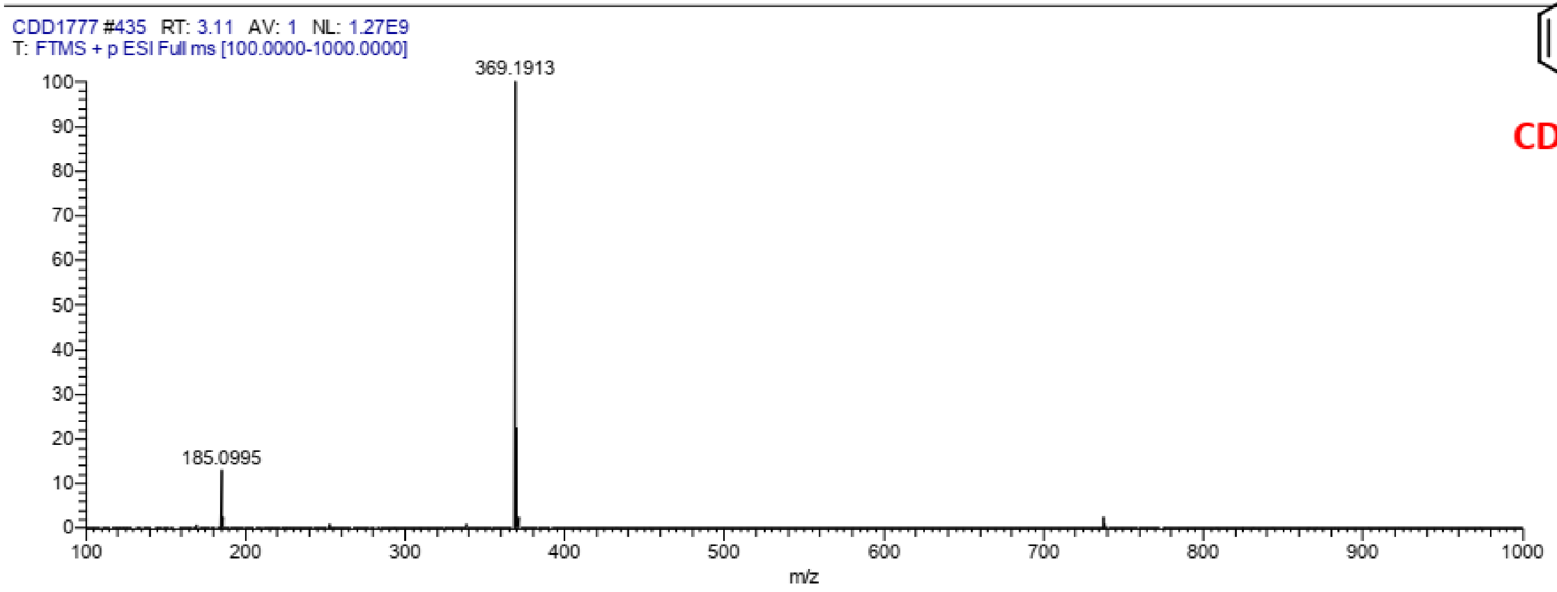
CDD1777 #435 RT: 3.11 AV: 1 NL: 1.27E9
T: FTMS + p ESI Full ms [100.0000-1000.0000]
369.1913
185.0995
m/z

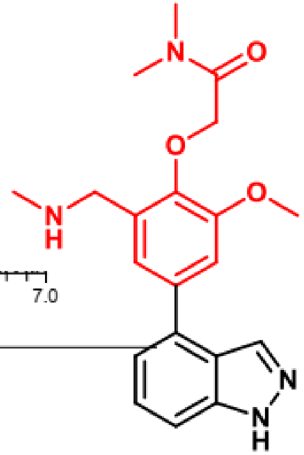

CDD-1777

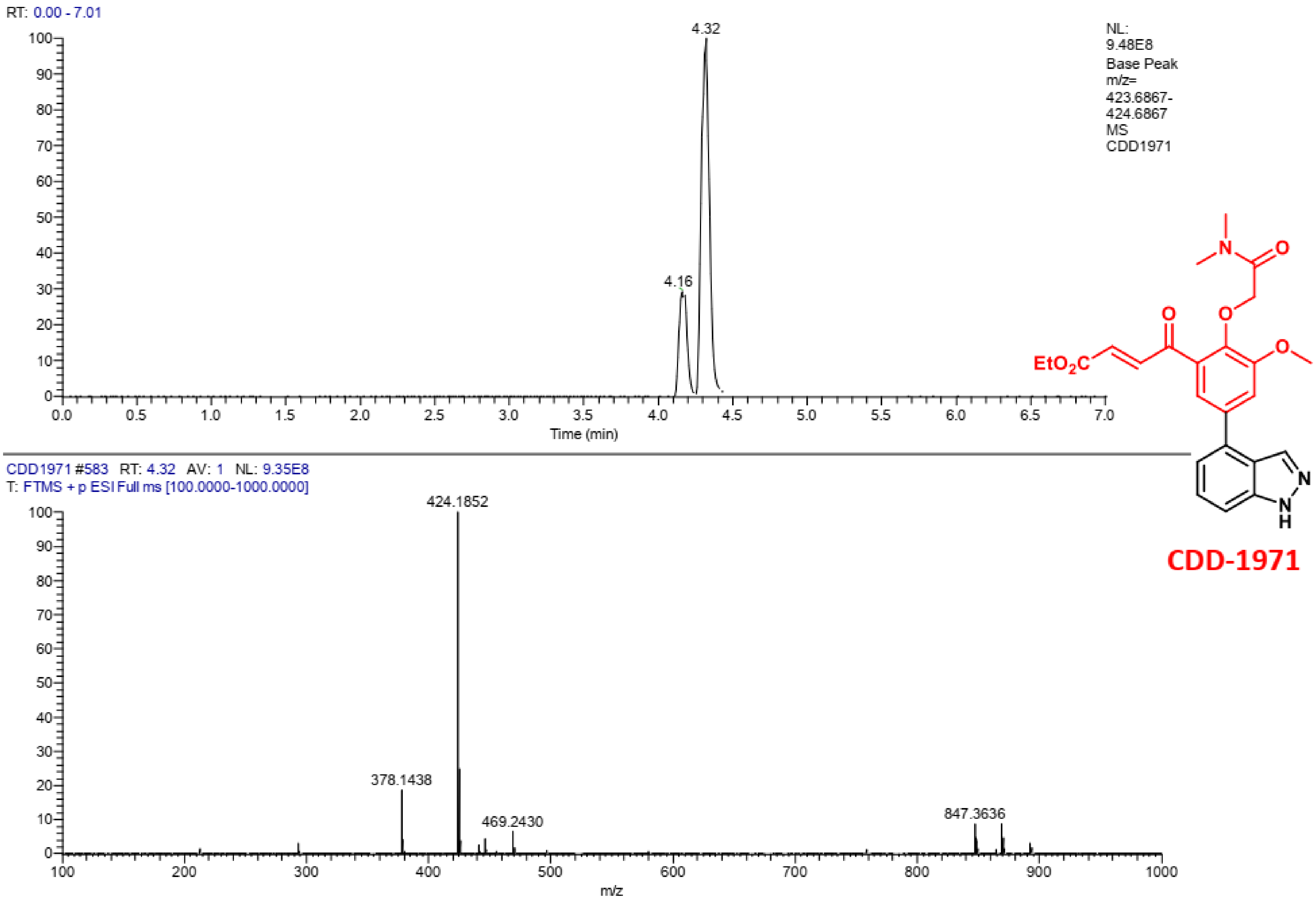
RT: 0.00 - 7.01
4.32
4.16
NL:
9.48E8
Base Peak
m/z=
423.6867-
424.6867
MS
CDD1971
Time (min)
CDD1971 #583 RT: 4.32 AV: 1 NL: 9.35E8
T: FTMS + p ESI Full ms [100.0000-1000.0000]
424.1852
378.1438
469.2430
847.3636
m/z
EtO2C

CDD-1971
